# Iron in a Cage: Fixation of a Fe(II)tpy_2_ Complex by Fourfold Interlinking

**DOI:** 10.1002/anie.202006340

**Published:** 2020-06-29

**Authors:** Thomas Brandl, Sven Johannsen, Daniel Häussinger, Nithin Suryadevara, Alessandro Prescimone, Stefan Bernhard, Manuel Gruber, Mario Ruben, Richard Berndt, Marcel Mayor

**Affiliations:** ^1^ Department of Chemistry University of Basel St. Johanns-Ring 19 4056 Basel Switzerland; ^2^ Institut für Experimentelle und Angewandte Physik Christian-Albrechts-Universität zu Kiel Leibnizstr. 19 24098 Kiel Germany; ^3^ Department of Chemistry Carnegie Mellon University Pittsburgh PA 15213 USA; ^4^ Institute of Nanotechnology Karlsruhe Institute of Technology (KIT) Hermann-von-Helmholtz-Platz 1 76344 Eggenstein-Leopoldshafen Germany; ^5^ Institut de Physique et Chimie des Matériaux de Strasbourg (IPCMS) CNRS-Université de Strasbourg 23, rue de Loess, BP 43 67034 Strasbourg cedex 2 France; ^6^ Lehn Institute of Functional Materials School of Chemistry Sun Yat-Sen University Guangzhou 510275 China

**Keywords:** cage compounds, Fe terpyridine, hexadentate ligand, macrocyclic ligands, Wittig reactions

## Abstract

The coordination sphere of the Fe(II) terpyridine complex **1** is rigidified by fourfold interlinking of both terpyridine ligands. Profiting from an octa‐aldehyde precursor complex, the ideal dimensions of the interlinking structures are determined by reversible Schiff‐base formation, before irreversible Wittig olefination provided the rigidified complex. Reversed‐phase HPLC enables the isolation of the all‐*trans* isomer of the Fe(II) terpyridine complex **1**, which is fully characterized. While temperature independent low‐spin states were recorded with superconducting quantum interference device (SQUID) measurements for both, the open precursor **8** and the interlinked complex **1**, evidence of the increased rigidity of the ligand sphere in **1** was provided by proton T_2_ relaxation NMR experiments. The ligand sphere fixation in the macrocyclized complex **1** even reaches a level resisting substantial deformation upon deposition on an Au(111) surface, as demonstrated by its pristine form in a low temperature ultra‐high vacuum scanning tunneling microscope experiment.

## Graphical abstract

**Caging an Fe(II) ion**: Like a cube the fourfold interlinked bisterpyridine ligand surrounds the Fe(II) ion. The post‐coordinative macrocyclization rigidifies the ligand sphere and guaranties the preservation of the properties of the complex.

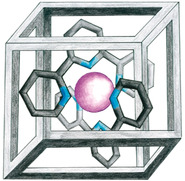

Switches are essential components in electronic devices and driven by the ongoing miniaturization, molecular switches move into the focus of interest.^1^, ^2^, ^3^ While various structures triggered by light, heat, or (electro) chemical potential have been reported,^4^, ^5^, ^6^ molecular switches responding on the applied potential and/or mechanical pressure are less frequent, in spite of their better miniaturization potential.

Iron bis‐terpyridine (Fe(II)tpy_2_) complexes are particularly appealing as potential switching motives due to their coordination geometry sensitive spin crossover (SCO) properties. And indeed, single molecule junctions with tailor‐made Fe(II)tpy_2_ complexes investigated at low temperature displayed bistability features pointing at SCO events triggered by the distortion of the ligand sphere. Examples are the mechanical spin‐state manipulation of a homoleptic Fe(II)tpy_2_ complex in a mechanically controlled break junction (MCBJ),^7^ or electric field (*E*‐field) sensing junctions with heteroleptic Fe(II)tpy_2_ complexes with push–pull systems integrated in one tpy‐ligand.^8^ In the latter case, the reliability of the *E*‐field triggered switching was recently improved by separating the active Fe(II) complex from the electrodes.^9^

While these MCBJ based single molecule experiments showing the potential of Fe(II)tpy_2_ complexes as switchable subunits were encouraging, scanning tunneling microscopy (STM) may provide insights into the arrangement of the molecule at the electrode surface and its electron transport. In a first attempt, the arrangement of both tpy‐ligands around the central Fe^2+^ ion was stabilized by macrocyclization, providing chiral Fe(II)tpy_2_ complexes.^10^ Upon deposition on Au(111) in an ultra‐high vacuum (UHV) STM set‐up however, the complex disintegrated as clearly documented by the macrocycle spread on the metal surface (Supporting Information, Figure SI22).^11^

Herein, we report the next generation of Fe(II)tpy_2_ complexes with rigidified coordination sphere. Fourfold interlinking of both tpy‐ligands freezes their spatial arrangement further improving the stability of the central Fe(II)tpy_2_ complex in **1** (Figure 1).

**Figure 1:**
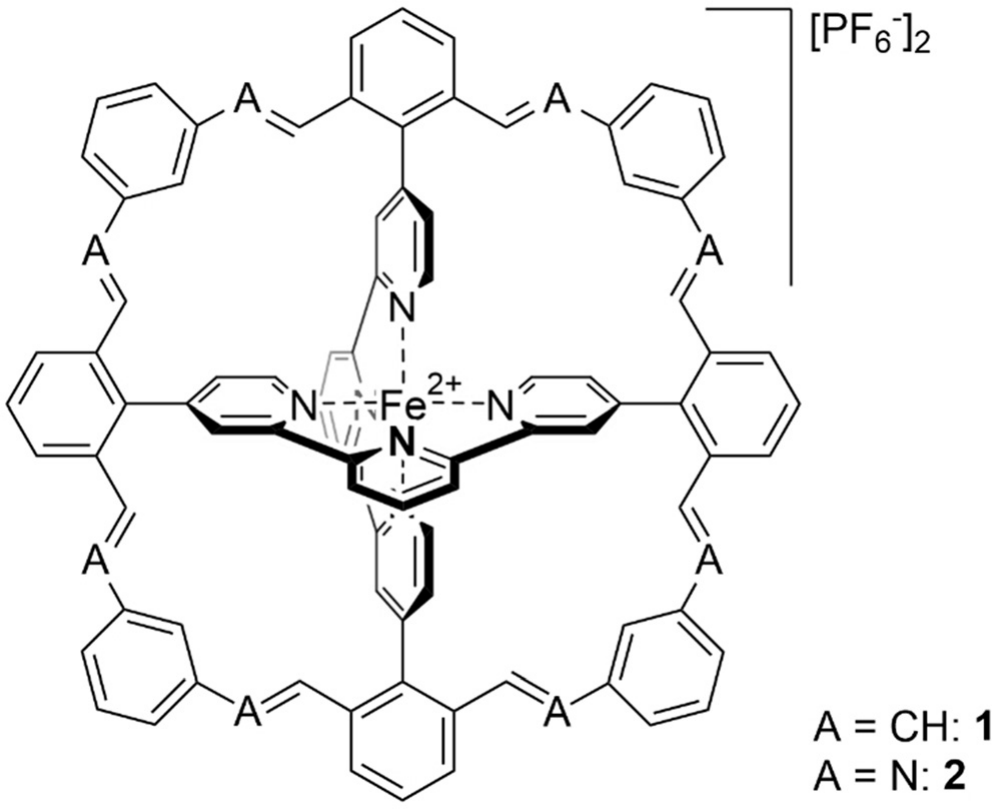
Fourfold interlinked Fe(II) terpyridine complexes **1** and **2**. While **1** was isolated and characterized, **2** was observed by mass spectrometry and served to identify linkers with dimensions suitable to bridge the two aldehydes.

Indeed, the coordination sphere of the isolated target complex **1** was considerably reinforced as displayed by *Carr‐Purcell‐Meiboom‐Gill*‐NMR experiments. The improved structural integrity of **1** even enabled its investigation by UHV STM experiments.

To explore a fourfold interlinking strategy, the octa‐aldehyde complex **8** (Scheme 1) moved into the focus of interest. The symmetry of the compound facilitates its synthesis and the exposed aldehyde functions enable a variety of potential interlinking reactions, like for example, the McMurry reaction,^12^ carbonyl–olefin metathesis,^13^ Schiff base condensation^14^ or *Wittig* olefination.^15^

**Scheme 1:**
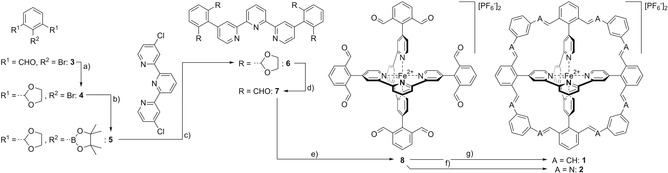
Synthesis of the Fe(II)tpy_2_ cage complexes **2** and **1**. Reagents and conditions: a) Ethylene glycol, *p*TsOH⋅H_2_O, toluene, reflux, 12 h, 100 %. b) (Bpin)_2_, KOAc, Pd(PPh_3_)_2_Cl_2_, 1,4‐dioxane, 100 °C, 18 h, 87 %. c) K_2_CO_3_, Pd(amphos)Cl_2_, toluene/water (5:1), 116 °C, 18 h, 75 %. d) PPTS, acetone/water (5:1), microwave (MW), 80 °C, 3 h, 85 %. e) 1.) FeCl_2_, DCM/MeOH (1:1), RT, 1 h, 2.) NH_4_PF_6_, water, 12 h, 94 %. f) *m*‐phenylenediamine, *p*TsOH⋅H_2_O, DCM to DCE, reflux, 10 d, n.d. g) (1,3‐phenylenebis(methylene))bis(triphenylphosphonium) dibromide, DBU, DMF, −20 °C, 12 h, 4 %.

Indeed, as displayed in Scheme 1, the octa‐aldehyde Fe(II)tpy_2_ complex **8** was obtained in five steps. First the aldehyde groups of commercially available 2‐bromoisophthalaldehyde **3** were protected by refluxing **3** in toluene in the presence of ethylene glycol and catalytic amounts of *p*‐toluenesulfonic acid. By removing the water with a Dean–Stark apparatus the reaction was driven to completion and the bis‐dioxolan species **4** was isolated in quantitative yield. The boronic acid pinacol ester **5** was obtained in 87 % yield by exposing **4** to standard Miyaura borylation conditions. Thus **4** was kept for 18 h at 100 °C in 1,4‐dioxane with bis(pinacolato)diboron (Bpin)_2_ as borane source, bis(triphenyl‐phosphine)palladium(II) dichloride (Pd(PPh_3_)_2_Cl_2_) as catalyst, and potassium acetate (KOAc) as base. The required 4,4′′‐dichloro‐2,2′:6′,2′′‐terpyridine building block was assembled following our already published protocol.^16^ The catalytic system reported by Guram and co‐workers^17^ enabled the twofold Suzuki–Miyaura cross‐coupling reaction with **5**. Using potassium carbonate (K_2_CO_3_) as base and bis(di‐tert‐butyl(4‐dimethylaminopehnyl)phosphine) dichloropalladium (II) (Pd(amphos)Cl_2_) as catalyst with a toluene/water solvent mixture in a sealed tube at 116 °C, provided the desired tpy‐ligand **6** in 75 % isolated yield after column chromatography (CC). To liberate the aldehydes prior to Fe^2+^ coordination, two reported protocols were combined.^18^, ^19^ The tetra‐aldehyde tpy ligand **7** was obtained in 85 % yield by treating **6** with pyridinium *p*‐toluenesulfonate as proton source in a mixture of acetone and water at 100 °C in a microwave reactor. The homoleptic Fe(II) complex **8** was assembled by the addition of iron (II) chloride (FeCl_2_) to a solution of the ligand **7** in a dichloromethane (DCM) and methanol (MeOH) solvent mixture. An immediate color change to deep purple indicated the complex formation. After solvent removal and precipitation from a NH_4_PF_6_ water solution, the octa‐aldehyde complex **8** was isolated in 94 % yield.

All new compounds were fully characterized by ^1^H‐ and ^13^C‐NMR spectroscopy, and high‐resolution mass spectrometry. Single crystals suitable for *x*‐ray diffraction of the complex **8** were obtained by diffusing diethyl ether vapor into an acetonitrile solution of **8**. The solid‐state structure (Figure 2) not only corroborated the identity of the complex, but also gave insight into the intramolecular distances between the aldehyde groups. The inter‐aldehyde O–O spacing varied between 3.60 and 7.15 Å.

**Figure 2:**
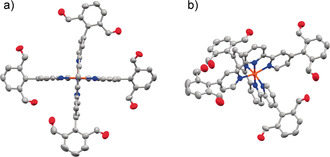
Solid state structure of complex **8** with rotation ellipsoids set at 50 % probability. a) Top view along a N–Fe–N axis and b) side view. Hydrogen atoms and the PF_6_^−^ counter ions were omitted for clarity. Color code: N: blue, Fe: orange, C: gray, O: red.

To explore both, the feasibility of the fourfold interlinking strategy and the ideal dimension of the interlinking units, the Schiff base condensation was selected as test reaction. The reversibility of the imine bond should enable to investigate the thermodynamic stability and accessibility of the rigidified complex.

Initial attempts using ethylene diamine as linker (MM2 calculated N–N distance: 3.80 Å) failed, the focus moved towards aromatic diamines, hoping that the increased delocalization of the imine π‐systems additionally favors the interlinking. And indeed, with *meta*‐phenylenediamine (MM2 calculated N–N distance: 4.5 Å) the formation of an eightfold condensation product with **8** was observed among a variety of possible condensation products by direct injection electrospray mass spectrometry (DI‐ESI‐MS, Figure SI18 in the Supporting Information). While reaction conditions favoring the formation of the fourfold interlinked complex **2** could be found (8 equivalents of the diamine in refluxing 1,2‐dichloroethane (83.5 °C) for 10 days, Figure SI19 in the Supporting Information), all attempts to isolate the fourfold interlinked structure **2** failed, pointing at the lability of the imine bonds. Numerous efforts to reduce the imine linkers to more robust amine bonds employing a variety of reducing agents were also not successful.

Thus, in spite the fact that the mass signal in the DI‐ESI‐MS remains the only indication of the existence of the fourfold interlinked complex **2**, the experiments were indicative to identify the dimension of the ideal interlinking structure.

In order to improve the stability of the fourfold interlinked complex the strategy was to replace the reversible and hydrolysis sensitive imine‐bonds of **2** by the olefins in **1**. Like that the interlinking structure would be of comparable dimension and by using Wittig chemistry, **1** should be accessible from the same octa‐aldehyde precursor Fe(II)tpy_2_ complex **8**. Indeed, treating **8** with 10 equivalents of (1,3‐phenylenebis(methylene))bis(triphenylphosphonium) dibromide in −20 °C cold dimethylformamide using 1,8‐diazabicyclo[5.4.0]undec‐7‐ene as a base resulted in a crude reaction mixture with the mass of the expected fourfold interlinked complex **1** (1330 *m*/*z*) as main signal recorded in a matrix‐assisted laser desorption ionization mass spectrometer (MALDI‐MS). However, even though the interlinking reaction proceeded smoothly, the purification remained challenging due to the lack of conformational selectivity in the double bonds formed by the Wittig reaction. With the *D*_2*h*_ symmetry of **1**, 64 different isomers are possible in theory due to *cis*/*trans* configurations of the eight double bonds. Even though not all of them are likely to be formed, the uninterpretable NMR spectrum of the crude reaction mixture points at several isomers being present. Thus, the crude material was subjected to reversed‐phase HPLC and after two runs, the all‐*trans* isomer could be separated in 4 % yield (Supporting Information, Figure SI23). The similar MALDI‐MS signals of all isolated peaks confirmed the hypothesis of various olefin isomers being formed during the interlinking Wittig reaction. The ^1^H‐NMR spectrum of the all‐*trans* isomer of **1** is displayed in Figure 3. The rather simple spectrum corroborates the identity of the highly symmetric complex, with the 16.4 Hz coupling constants of both alkene protons (magenta in Figure 3) documenting the *trans*‐connection of the double bonds. The all‐*trans* isomer of **1** was further characterized by ^1^H‐, ^13^C‐, COSY‐, ROESY‐, HSQC‐, and HMBC‐NMR spectroscopy, UV/Vis spectroscopy, and high‐resolution electrospray ionization mass spectrometry (HR‐ESI‐MS, see Supporting Information).

**Figure 3:**
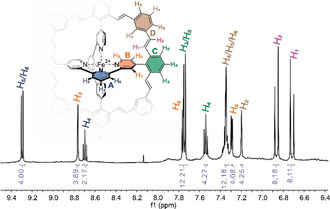
^1^H‐NMR of the all‐*trans* isomer of **1** with assignment of the signals.

Of particular interest was the rigidity gained by the fourfold interlinking in the all‐*trans* isomer of **1**, which was analyzed by the comparison of the spin–spin relaxation times T_2_ for the protons of **1** (all‐*trans* isomer) and the octa‐aldehyde precursor complex **8**. In these complexes of comparable molecular weight, the spin–spin relaxation correlates with the intramolecular mobility of the spin and is thus a sensitive probe for the flexibility of the spin bearing protons. Using a Carr‐Purcell‐Meiboom‐Gill (CPMG) spin echo pulse sequence, T_2_ was measured for all aryl protons (Supporting Information, Figure SI15). The recorded values display for every proton a decreased T_2_ by a factor of 1.20 to 1.75 for the all‐*trans* isomer of **1** compared to **8** (Supporting Information, Table SI3), pointing at a substantial rigidification of the ligand sphere upon fourfold interlinking. The experiment even allows to localize the mobility of individual subunits in both complexes. While the protons of the central pyridine ring **A** are the most static part, the *ortho*‐positioned **B** rings are more mobile in both compounds. Interestingly, the protons of the **C** and **D** rings as subunits of the linker structure of **1** (all‐*trans* isomer) displayed flexibilities in between the values recorded for **A** and **B**, suggesting a tight fixation of the interlinking bridges. In contrast, the **C** ring of **8** displayed an up to three‐fold T_2_ increase, as expected for the higher degree of freedom of a rotating subunit. Another observation pointing at the rigidity of **1** is the increased uniformity of the values recorded for T_2_ (max/min: 1.52) compared with the ones observed for **8** (max/min: 3.04). While the molecular weight increase from **8** to **1** is with 20.9 % too small to rationalize the recorded differences in T_2_, an aggregation of **1** to oligomers might be an alternative explanation for the observation. To disqualify this hypothesis, the hydrodynamic radii of both complexes were determined by diffusion ordered NMR spectroscopy (Supporting Information, Figure SI16 and SI17). The observed difference of 5.2 % in diffusion coefficients corresponds to a mass increase of 16.4 % of **1** compared to **8**, corroborating that both complexes were analyzed as dissolved monomeric complexes.

Density functional theory (DFT) calculations were performed to obtain a better understanding of the electronic structure of **1** and to gauge the ring strain energy of the large ring system surrounding the [Fe(tpy)_2_]^2+^ core of the complex. DFT calculations were completed for compound **1** using a B3LYP functional and a 6‐31G (d,p) basis set using the Gaussian 09 suite (Figure 4). An unrestricted geometry optimization revealed that **1** optimized to retain the *D*_2*d*_ symmetry commonly observed for octahedral bis‐2,2′:6′,2′′‐terpyridine complexes. The flexibility of the surrounding ring structure allowed for a slight bending; the direction of this deformation was identical for the quarter‐segments of the ring diagonally opposed to each other. The electronic structure retained some of the features of the parent complex: The low energy pyridinic π* orbitals were also observed for the terpyridine moiety of **1** and covered the orbitals from LUMO to LUMO+7; at higher energies the π* orbitals of the ring structure started mixing in. The HOMO of the parent [Fe(tpy)_2_]^2+^ is located on the Fe d orbitals and is the origin of the well‐studied MLCT transition of the complex. In contrast, the calculated HOMO of compound **1** is exclusively located on the large π system of the ring and the first metal contributions do not reveal itself until reaching the 1.5 eV more stable HOMO‐8 and the degenerate pair HOMO‐9 and HOMO‐10. The reaction enthalpy of an isodesmic reaction separating the peripheral ring and the metal complex moiety was estimated by DFT calculations and revealed that the overall ring strain energy for **1** is around 16 kcal mol^−1^. The strain is distributed over 4 ring systems formed by more than 70 atoms making it negligible. Details of these calculations are documented in the Supporting Information (Figure SI24).

**Figure 4:**
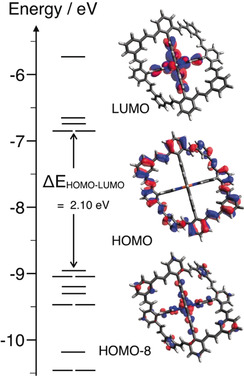
Frontier molecular orbital diagram of **1** generated from DFT calculations (B3LYP/6‐31G (d,p)). The rendered 98 % surfaces of selected orbitals are also depicted. The first filled orbital with a significant metal contribution is HOMO‐8, which is in stark contrast to the metal‐centered HOMO observed for the parent [Fe(tpy)_2_]^2+^ complex.

The rigidity of **1** (all‐*trans* isomer) was also observed in its magnetic behavior recorded in a superconducting quantum interference device (SQUID) magnetometer. In a temperature range between 5 K and 365 K, complex **1** remained in its low spin state. While the observation supports the claim of a fixed coordination sphere in the all‐*trans* isomer of **1**, it does not qualify to account for an increased rigidity by fourfold interlinking, as also the open octa‐aldehyde **8** remains in its low‐spin state in this temperature range.

To our delight, the structural integrity of **1** was even preserved upon deposition on a gold surface in a STM experiment. Employing an electrospray setup with in line mass selection^20^ to deposit **1** on an Au(111) surface (Supporting Information, Figure SI21) enabled its deposition and subsequent investigation of the sample with a STM at 4.4 K under UHV conditions. The electrospray deposition ensured the absence of counter ions on the surface. A STM constant‐current topograph of a typical molecule is displayed in Figure 5 a. The adsorbate most probably corresponds to intact **1**, although a degree of distortion may be present. The geometry of **1** on Au(111) is most likely the one represented in the side view (Figure 5 b), exposing three dominant extrusions into vacuum (two phenyl groups of interlinking subunits and the central pyridine moiety of a ligand). In agreement with this simple model, the topograph exhibits three protrusions with submolecular dimensions. The distance between the outermost protrusions (1.6 nm) approximately matches the phenyl to phenyl distance of **1** (see scale bars in Figure 5 a and b). The apparent height of the molecular adsorbates (Figure 5 a) of approximately 550 pm is considerably higher than the 180 pm observed for the flattened terpyridine ligands of the first generation of Fe(II)tpy_2_ complexes disintegrating upon deposition on Au(111).^11^ This observation is in agreement with a limited adsorption‐induced distortion of **1**, further documenting the structural integrity gained by fourfold interlinking of the tpy ligands. The charge of **1** cannot be unambiguously clarified from the present data.

**Figure 5:**
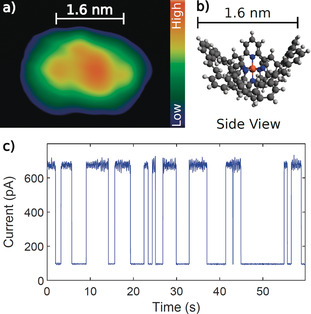
a) Constant‐current STM topograph of **1** on Au(111). Current *I*=5 pA and sample voltage *V=*−0.5 V. b) Side‐view model of **1**. Orange, blue, dark gray, and light gray spheres represent iron, nitrogen, carbon, and hydrogen atoms, respectively. c) Time series of the tunneling current measured at *V*=0.9 V with the tip position frozen over the center of **1**.

While spin switching of individual complexes has been evidenced on partially insulating layers,^21^, ^22^, ^23^, ^24^, ^25^, ^26^ the switching on metal surfaces remains a challenge with a few exceptions.^27^, ^28^ The strong molecule–substrate interaction on metal surfaces often affects the integrity and functionality of adsorbed complexes.^11^, ^26^ The fixation of the ligands by fourfold interlinking not only increases the structural integrity of **1**, but also is expected to preserve the functionality of the Fe(II)tpy_2_ complex caged in the center of the macrocycle upon adsorption, which was not the case for less tightly fixed Fe(II)tpy_2_ model complexes (Supporting Information, Figure SI22). And indeed, two‐level fluctuations of the tunneling current are observed at slightly elevated sample voltages (*V*=0.9 V in Figure 5 c). Even though the finding is consistent with structural and electronic changes expected upon spin transitions in spin crossover complexes, an unambiguous identification of the underlying switching mechanism will require further experiments, most likely using large magnetic fields.

In summary, the tight fixation of the coordination sphere of a Fe(II)tpy_2_ complex by fourfold interlinking of the suitably decorated tpy ligands is reported. Profiting from the octa‐aldehyde precursor **8**, the ideal dimensions of the interlinking subunits was determined by Schiff base condensation, while the caged iron complex **1** was obtained by Wittig olefination. The all‐*trans* isomer of **1** was isolated by HPLC and fully characterized by NMR spectroscopy, UV/Vis spectroscopy, and mass spectrometry. The rigidity of the ligand sphere was analyzed by CPMG‐NMR experiments and increased the integrity of the caged complex to a level enabling to preserve its structure and functionality upon adsorption on metal surfaces. Low‐temperature STM experiments evidenced preserved switching functionality of the complex.

The approach holds perspectives not only for spin‐switch functionality, but also to couple the spin of the complexes to more reactive ferromagnetic substrates,^29^ of relevance for molecular spintronics. Currently, we are exploring both, the structural diversity of caged complexes as well as their peripheral functionalization.

## Conflict of interest

The authors declare no conflict of interest.

## Supporting information

As a service to our authors and readers, this journal provides supporting information supplied by the authors. Such materials are peer reviewed and may be re‐organized for online delivery, but are not copy‐edited or typeset. Technical support issues arising from supporting information (other than missing files) should be addressed to the authors.

Supplementary
