# Supplementary material for: Iron in a Cage: Fixation of a Fe(II)tpy2 Complex by Fourfold Interlinking
Source: Angew Chem Int Ed Engl. 2020 Jun 29;59(37):15947–52. doi: 10.1002/anie.202006340 (PMC7540000; doi:10.1002/anie.202006340)
Supplement: Supplementary file 1 — Supplementary [file ANIE-59-15947-s001.pdf]

Angewandte Chemie
Eine Zeitschrift der Gesellschaft Deutscher Chemiker

Supporting Information

Iron in a Cage: Fixation of a Fe(II)tpy$_2$ Complex by Fourfold Interlinking

*Thomas Brandl, Sven Johannsen, Daniel Häussinger, Nithin Suryadevara, Alessandro Prescimone, Stefan Bernhard, Manuel Gruber, Mario Ruben, Richard Berndt, and Marcel Mayor**

anie_202006340_sm_miscellaneous_information.pdf

Table of Contents

Experimental Procedures 3

^{1}H-, ^{13}C-NMR ($CDCl_3$, 400/101 MHz, 22 °C) and HR-ESI-MS spectra of compound **4** 14

^{1}H-, ^{13}C-NMR ($CDCl_3$, 400/101 MHz, 22 °C) and HR-ESI-MS spectra of compound **5** 16

^{1}H-, ^{13}C-NMR (CD_2Cl_2, 400/101 MHz, 22 °C) and HR-ESI-MS spectra of compound **6** 18

^{1}H-, ^{13}C-NMR (CD_2Cl_2, 400/101 MHz, 22 °C) and HR-ESI-MS spectra of compound **7** 20

^{1}H-, ^{13}C-, COSY-, ROESY-, HSQC-, HMBC-NMR (Acetone-d_6/Acetonitrile-d_3, 600/400/151/101 MHz, 25 °C/22 °C) with full assignment and HR-ESI-MS spectra of compound **8** 22

HR-ESI-MS spectra of compound **2** 29

^{1}H-, ^{13}C-, COSY-, ROESY-, HSQC-, HMBC-NMR (Acetone-d_6, 600/151 MHz, 25 °C) with full assignment and HR-ESI-MS spectra of compound **1** 30

UV-Vis Spectra of Compound **8** and Compound **1** 37

Crystal Data of **8** and Unsuccessful Crystallization Attempts of **1** 48

SQUID Measurements 50

IR-Spectra 52

Determination of the Spin-Spin Relaxation Times T_2 of Compound **8** and all-*trans* Isomer **1** by NMR Spectroscopy 54

Monitoring the Imine Condensation towards the Fourfold Interlinked Complex **2** by DI-ESI-MS and NMR Spectroscopy 58

Scanning Tunneling Microscope (STM) Experiments of Complex **1** 60

Fe(II)tpy$_2$ Model Complexes of Varying Rigidity in STM Experiments 60

HPLC Chromatograms of Complex **1** 62

DFT Calculations 63

Experimental Procedures

General Procedures: All chemicals were directly used for the synthesis without further purification, unless stated differently. Solvents for photophysical measurements were HPLC grade. Dry solvents were used as crown cap and purchased from *Acros Organics* and *Sigma-Aldrich*. NMR solvents were obtained from *Cambridge Isotope Laboratories, Inc.* (Andover, MA, USA). All NMR spectra were recorded on *Bruker Avance III HD* instruments operating at either 400.13 or 600.13 MHz proton frequency. The spectrometers were equipped with broadband direct detection probes (BBFO, 400 and 600 MHz) or a cryogenic quadruple indirect detection probe (QCI, 600 MHz), all with self-shielded z-gradients. Chemical shifts (δ) are reported in parts per million (ppm) relative to the residual solvent peak. Coupling constants (*J*) are given in Hertz (Hz). The PFGSE (dosy) experiments were performed using a pulse sequence with bipolar gradients for diffusion and two spoil gradients (ledbpgp2s).[1] 16 Increments were recorded with linear increasing gradient strength from 5 to 95%, using 16 scans per increment and a recycling delay of 3s. Processing was performed with a two-parameter fit (I(0) and D) using the Bruker Topspin 3.5 software package.[2] The T2 relaxation times were determined with a Carr-Purcell-Meiboom-Gill (CPMG) sequence.[3] A recycling delay of 3 s and an echo time of 1 ms were applied for 8 increments with the following loop counters: 2,16,64,128,256,512,768,1024. Each increment was repeated 16 or 32 times and processed using the *Dynamics* modul of the Bruker Topspin 3.5 software package.[4]

Gas Chromatography (GC-MS) was performed on a *Shimadzu* GCMS-QP2010 SE gas chromatograph system, with a ZB-5HT inferno column (30 m x 0.25 mm x 0.25 mm), at 1 mL/min He-flow rate (split = 20:1) with a *Shimadzu* mass detector (EI 70 eV) was used. For high resolution mass spectra (HRMS) a HR-ESI-ToF-MS measurement on a *maXis™ 4G* instrument from *Bruker* was performed. MALDI-ToF mass spectra were performed on a *Bruker microflex*™ mass spectrometer, calibrated with CsI_3, and α-cyano-4-hydroxycinnamic acid was used as matrix. Column chromatography was performed with SiliaFlash® P60 from *SILICYCLE* with a particle size of 40-63 µm (230-400 mesh) and for TLC *Silica gel 60* F_{254} glass plates with a thickness of 0.25 mm from *Merck* were used. The detection was observed with a UV-lamp at 254 or 366 nm. For analytical HPLC a Shimadzu LC-20AD and a LC-20AT HPLC, respectively, was used equipped with a diodearray UV/Vis detector (SPD-M10A VP from Shimadzu, λ = 200-600 nm) and a column oven Shimadzu CTO-20AC. The used column for reverse phase was a Reprosil 100 C18, 5 µm, 250 x 16 mm; Dr. Maisch GmbH. For preparative HPLC a Shimadzu LC-20Ap and a LC-20Ap was used equipped with a diode array

[1] D. Wu et al., *J. Magn. Res. A*, **1995**, *115*, 260 – 264.
[2] Bruker BioSpin GmbH, Rheinstetten, (**2017**).
[3] S. Meiboom and D. Gill, *Rev. Scientific Instruments*, **1958**, *29*, 688-691.
[4] Bruker BioSpin GmbH, Rheinstetten, (**2017**).

UV/Vis detector (SPD-20A from Shimadzu, λ = 200-600 nm). The used column was a Reprosil 100 C18, 10 μm,250 x 30 mm; Dr Maisch GmbH.

- **2,2'-(2-bromo-1,3-phenylene)bis(1,3-dioxolane) (4)**

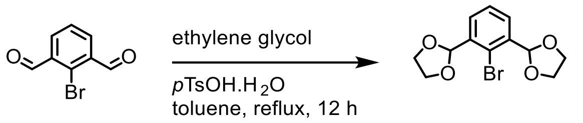

A 50 mL round-bottomed flask was charged with 2-bromoisophthaldehyde (**3**, 500 mg, 2.35 mmol, 1.0 eq), *p*TsOH·H_2O (9.03 mg, 47.0 µmol, 2 mol-%), ethyleneglycol (788 µL, 14.1 mmol, 6.0 eq.) and toluene (50 mL). The reaction mixture was heated to reflux for 12 hours and the formed water was removed using a Dean-Stark apparatus. The reaction mixture was cooled to room temperature, was washed with aqueous saturated $NaHCO_3$ solution and was extracted with DCM. The combined organic layers were washed with brine, dried over $MgSO_4$, filtered and concentrated under reduced pressure to yield the product as a white solid (711 mg, 2.36 mmol, 100%). The product was sufficiently pure and was used for the next reaction without further purification.

Analytical Data for 4:

^{1}H NMR (400 MHz, $CDCl_3$, 22 °C) δ 7.62 (d, *J* = 7.5 Hz, 2H), 7.37 (t, *J* = 7.7 Hz, 1H), 6.19 (s, 2H), 4.20 – 4.01 (m, 8H).

^{13}C NMR (101 MHz, $CDCl_3$, 22 °C) δ 137.55, 128.67, 127.39, 123.78, 102.69, 65.54.

GC-MS (EI +, 70 eV): m/z (%) = 299 (26) [M+], 221 (16) [M+-Br], 186 (15), 184 (15), 89 (17), 73 (100).

HRMS (ESI-ToF): calc. for $[C_{12}H_{14}BrO_4]^+$ 301.0070; found 301.0075.

- **2-(2,6-di(1,3-dioxolan-2-yl)phenyl)-4,4,5,5-tetramethyl-1,3,2-dioxaborolane (5)**

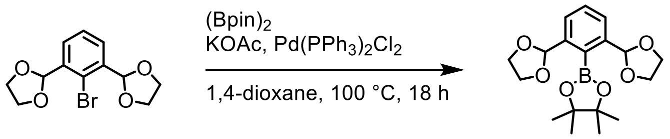

An oven-dried 100 mL Schlenk tube was charged with 2,2'-(2-bromo-1,3-phenylene)bis(1,3-dioxolane) (**4**, 666 mg, 2.21 mmol, 1.0 eq.) KOAc (438 mg, 4.42 mmol, 2.0 eq.), $(Bpin)_2$ (1.12 g, 4.42 mmol, 2.0 eq.) and 1,4-dioxane (30 mL). The reaction mixture was degassed with argon for 15 minutes, before $Pd(PPh_3)_2Cl_2$ (78.3 mg, 111 μmol, 5 mol-%) was added. The reaction mixture was degassed for further 15 minutes and then heated to 100 °C for 18 hours. After GC-MS indicated full consumption of the starting material, the reaction was cooled to room temperature, concentrated and purified by flash column chromatography (cyclohexane:ethyl acetate = 3:1) to obtain the product as white solid (667 mg, 1.92 mmol, 87%).

Analytical Data for 5:

^{1}H NMR (400 MHz, $CDCl_3$, 22 °C) δ = 7.52 (d, J = 7.7 Hz, 2H), 7.40 – 7.31 (m, 1H), 6.16 (s, 2H), 4.05 – 3.93 (m, 8H), 1.40 (s, 12H).

^{13}C NMR (101 MHz, $CDCl_3$, 22 °C) δ = 142.62, 128.94, 125.39, 102.90, 83.95, 64.80, 25.37.

GC-MS (EI +, 70 eV): m/z (%) = 348 (10) [M+], 303 (33), 203 (100), 202 (30), 133 (40), 132 (25), 73 (45).

HRMS (ESI-ToF): calc. for $[C_{18}H_{26}BO_6]^+$ 349.1820; found 349.1819.

- **4,4''-bis(2,6-di(1,3-dioxolan-2-yl)phenyl)-2,2':6',2''-terpyridine (6)**

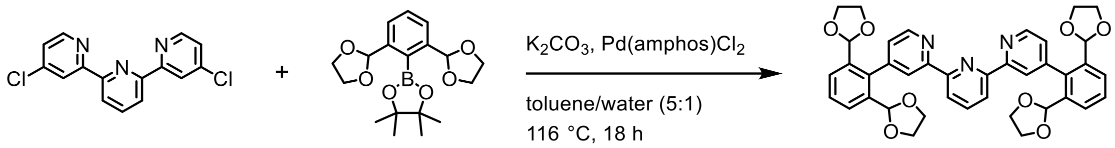

An oven-dried 25 mL round bottom flask was purged with argon and charged with 4,4''-dichloro-2,2':6',2''-terpyridine (50.0 mg, 165 μmol, 1.0 eq.), 2-(2,6-di(1,3-dioxolan-2-yl)phenyl)-4,4,5,5-tetramethyl-1,3,2-dioxaborolane (**5**, 144 mg, 413 μmol, 2.5 eq.), K_2CO_3 (137 mg, 990 μmol, 6.0 eq.) and bis(di-tert-butyl(4-dimethylaminopehnyl)-phosphine) dichloropalladium (II) (17.5 mg, 24.7 μmol, 15 mol-%.). The reactants were suspended in an extensively degassed solvent mixture of toluene/water (5:1 mL). The flask was sealed and heated to slight reflux at 116 °C oil bath temperature for 18 hours. The mixture was diluted with DCM and washed with water and brine, dried over $MgSO_4$, filtered and concentrated under reduced pressure. The crude material was purified by flash column chromatography (cyclohexane/acetone = 2:1 + 1% NH_4OH) to obtain the product as a white solid (83.0 mg, 123 μmol, 75%).

Analytical Data for 6:

^{1}H NMR (400 MHz, CD_2Cl_2, 22 °C) δ 8.70 (dd, *J* = 4.9, 0.9 Hz, 2H), 8.56 – 8.50 (m, 4H), 8.01 (t, *J* = 7.9 Hz, 1H), 7.69 (d, *J* = 7.8 Hz, 4H), 7.49 (t, *J* = 7.8 Hz, 2H), 7.29 (dd, *J* = 4.9, 1.7 Hz, 2H), 5.40 (s, 4H), 3.94 (dt, *J* = 7.1, 5.8 Hz, 4H), 3.84 – 3.63 (m, 12H).

^{13}C NMR (101 MHz, CD_2Cl_2, 22 °C) δ 156.23, 155.97, 148.80, 146.43, 139.32, 138.33, 136.37, 128.82, 127.89, 126.62, 123.23, 121.91, 101.50, 65.92, 65.72.

HRMS (ESI-ToF): calc. for $[C_{39}H_{36}N_3O_8]^+$ 674.2497; found 674.2507.

- **2,2'-([2,2':6',2''-terpyridine]-4,4''-diyl)diisophthalaldehyde (7)**

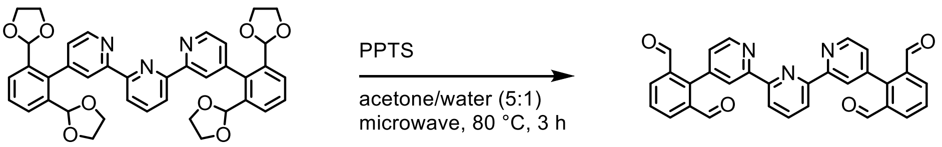

A 20 ml microwave vial was charged with 4,4''-bis(2,6-di(1,3-dioxolan-2-yl)phenyl)-2,2':6',2''-terpyridine (**6**, 80.0 mg, 119 µmol, 1.0 eq.), absolute acetone (12.75 ml) and pyridinium *p*-toluenesulfonate (133 mg, 524 µmol, 4.4 eq). Then, 2.25 ml water was added and the vial was sealed. The reaction mixture was heated to 80 °C for three hours in a microwave. The solvent was removed under reduced pressure and the remaining substance was diluted with DCM and the combined organic layers were washed with $NaHCO_3$, water and brine, dried over $MgSO_4$, filtered and concentrated under reduced pressure to obtain the product as white solid (50.0 mg, 101 µmol, 85%).

Analytical Data for 7:

^{1}H NMR (400 MHz, $CDCl_3$, 22 °C) δ 9.78 (d, *J* = 0.8 Hz, 4H), 8.83 (dd, *J* = 4.9, 0.9 Hz, 2H), 8.65 (d, *J* = 7.9 Hz, 2H), 8.54 (dd, *J* = 1.7, 0.9 Hz, 2H), 8.22 (d, *J* = 7.7 Hz, 4H), 8.07 (t, *J* = 7.9 Hz, 1H), 7.71 (tt, *J* = 7.7, 0.8 Hz, 2H), 7.35 (dd, *J* = 4.9, 1.7 Hz, 2H).

^{13}C NMR (101 MHz, CD_2Cl_2, 22 °C) δ 190.40, 156.41, 155.41, 149.71, 145.42, 143.15, 138.73, 134.82, 133.47, 129.80, 126.01, 122.78, 122.70.

HRMS (ESI-ToF): calc. for $[C_{31}H_{20}N_3O_4]^+$ 498.1448; found 498.1442.

- **2,2'-([2,2':6',2''-terpyridine]-4,4''-diyl)diisophthalaldehyde iron hexafluorophosphate (8)**

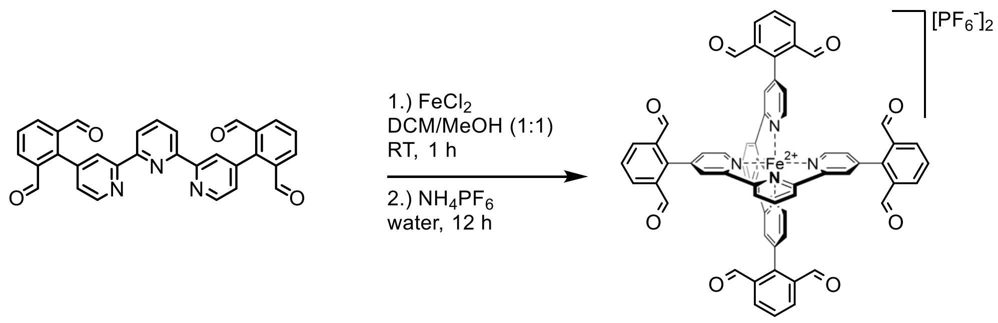

A 100 mL round-bottomed flask was charged with 2,2'-([2,2':6',2''-terpyridine]-4,4''-diyl)diisophthalaldehyde (**7**, 27.3 mg, 54.9 μmol, 2.0 eq.), and a solvent mixture of DCM/MeOH (50 mL, 1:1). Then, $FeCl_2$ (3.48 mg, 27.5 μmol, 1.0 eq.) was added. The reaction mixture was stirred for one hour at room temperature. The reaction mixture was concentrated to a minimum, before NH_4PF_6 salt (224 mg, 1.37 mmol, 50.0 eq.) dissolved in water (50 mL) was added in order to exchange the counter ion from Cl^- to PF_6^- and thus make the homoleptic $[Fe(tpy)_2]^{2+}[(PF_6)^-]_2$ – complex soluble in organic solvents. The formed precipitate was filtered and was washed with excess water. The purple solid was eluted with DCM, dried over $MgSO_4$, filtered and concentrated under reduced pressure. The pure product was obtained as purple solid (34.5 mg, 26.0 μmol, 94%).

Analytical Data for 8:

^{1}H NMR (400 MHz, Acetone-d_6, 22 °C) δ 9.72 (s, 8H), 9.33 (d, J = 8.1 Hz, 4H), 9.08 (dd, J = 1.9, 0.7 Hz, 4H), 8.90 (t, J = 8.1 Hz, 2H), 8.32 (d, J = 7.7 Hz, 8H), 7.97 (t, J = 7.7 Hz, 4H), 7.77 (dd, J = 5.8, 0.7 Hz, 4H), 7.52 (dd, J = 5.7, 1.9 Hz, 4H).

(600 MHz, Acetonitrile-d_3, 25 °C) δ 9.66 (d, J = 0.6 Hz, 8H), 8.85 (d, J = 8.1 Hz, 4H), 8.66 (t, J = 8.1 Hz, 2H), 8.62 (dd, J = 1.9, 0.7 Hz, 4H), 8.26 (d, J = 7.7 Hz, 8H), 7.87 (t, J = 7.7 Hz, 4H), 7.32 (dd, J = 5.8, 0.7 Hz, 4H), 7.26 (dd, J = 5.8, 1.8 Hz, 4H).

^{13}C NMR (101 MHz, Acetone-d_6, 22 °C) δ 190.71, 161.59, 158.89, 153.74, 147.42, 141.61, 139.42, 135.33, 135.28, 131.29, 129.65, 126.43, 125.41.

(151 MHz, Acetonitrile-d_3, 25 °C) δ 191.68, 161.69, 158.91, 154.23, 147.48, 142.23, 139.77, 135.77, 135.60, 131.83, 129.92, 126.67, 125.59.

HRMS (ESI-ToF): calc. for $[C_{62}H_{38}FeN_6O_8]^{2+}$ 525.1046; found 525.1052.

- **Imine iron terpyridine cage complex (2)**

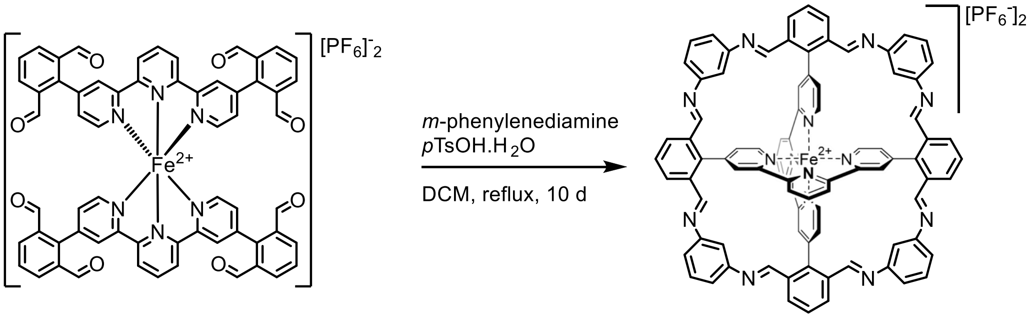

A 100 mL three-necked round bottom flask was charged with the iron complex (**8**, 10.3 mg, 7.68 µmol, 1.0 eq.), freshly distilled DCM (50 mL) and *p*-toluenesulfonic acid monohydrate (one crystal). The reaction mixture was heated to reflux *m*-phenylene-diamine (3.44 mg, 31.5 µmol, 4.1 eq.) dissolved in previously distilled DCM (10 mL) was added with a syringe pump over 10 hours. The reaction progress was controlled by ESI-MS analysis. ESI-MS clearly indicated the formation of, onefold linked, twofold linked, threefold linked and fourfold linked iron terpyridine cage complex. As the reaction was not completed after 118 hours, additional *m*-phenylenediamine (3.58 mg, 32.3 µmol, 4.3 eq.) was added. Furthermore the solvent was exchanged by 1,2-dichloroethane to allow higher temperature. The reaction was heated to reflux and after another 123 hours, ESI-MS indicated complete formation of the product. Purification attempts by column chromatography (SiO_2), reversed-phase HPLC or crystallization led to the re-opening of the reversible imine bonds.

Analytical Data for 2:

HRMS (ESI-ToF): calc. for $[C_{86}H_{54}FeN_{14}]^{2+}$ 669.2005; found 669.1999.

- **Wittig iron terpyridine cage complex (1)**

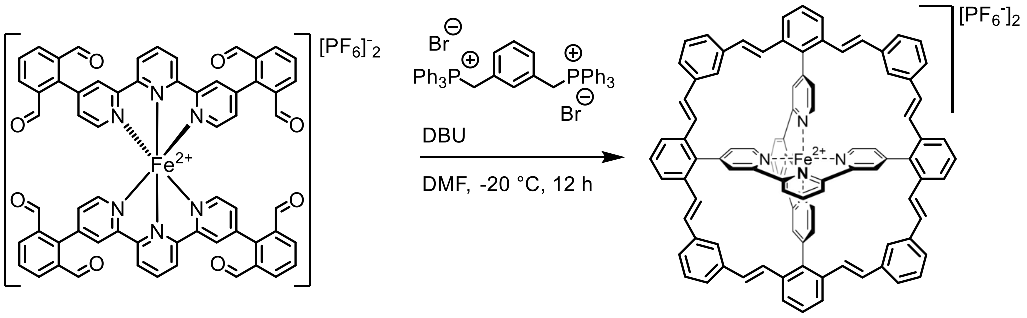

A 100 mL two-necked round bottomed flask was charged with the iron aldehyde complex (**8**, 100 mg, 74.6 µmol, 1.0 eq.), dry DMF (50 mL) and DBU (445 uL, 2.98 mmol, 40 eq.). The reaction mixture was cooled to -20 °C before the diphosphonium salts (588 mg, 746 µmol, 10 eq.) dissolved in dry DMF (5 mL) was added dropwise. MALDI-MS indicated complete conversion to the product. The reaction was stopped and concentrated to dryness under reduced pressure. The solid was redissolved in ethanol, filtered and subjected to preparative HPLC. With a solvent mixture EtOH/MeOH/H_2O/TEA/TFA (44.64:44.64:9.99:0.45:0.28) the product was separated from overreacted and only threefold interlinked side-product. As the Wittig reaction is not selective and it was assumed that the complex can have several conformers a second HPLC run using a solvent mixture of MeCN with 0.1% TFA / H_2O with 10 mM NH_4PF_6 (7:3) was performed. Using these conditions it was possible to isolate a small fraction of the complex (4.50 mg, 3.00 µmol, 4%) that could be identify as the all-trans isomer as a purple solid.

Analytical Data for 1:

^{1}H NMR (600 MHz, Acetone-*d*$_6$, 25 °C) δ 9.30 (d, *J* = 8.1 Hz, 4H), 8.76 (dd, *J* = 1.8, 0.7 Hz, 4H), 8.70 (t, *J* = 8.1 Hz, 2H), 7.78 – 7.71 (m, 12H), 7.54 (t, *J* = 7.8 Hz, 4H), 7.38 – 7.32 (m, 12H), 7.28 (dd, *J* = 5.7, 1.8 Hz, 4H), 7.20 (d, *J* = 1.7 Hz, 4H), 6.86 (d, *J* = 16.4 Hz, 8H), 6.71 (d, *J* = 16.4 Hz, 8H).

(600 MHz, Acetonitrile-*d*$_3$, 25 °C) δ 8.78 (d, *J* = 8.1 Hz, 4H), 8.51 (t, *J* = 8.1 Hz, 2H), 8.44 (dd, *J* = 1.8, 0.7 Hz, 4H), 7.69 (d, *J* = 7.9 Hz, 8H), 7.51 (t, *J* = 7.9 Hz, 4H), 7.37 – 7.31 (m, 4H), 7.30 – 7.26 (m, 8H), 7.14 (dd, *J*

= 5.8, 1.8 Hz, 4H), 7.10 (s, 4H), 6.79 (d, *J* = 16.3 Hz, 8H), 6.69 (d, *J* = 16.4 Hz, 8H).

^{13}C NMR (151 MHz, Acetone-d_6, 25 °C) δ 161.34, 159.22, 153.24, 151.17, 138.91, 138.20, 137.73, 135.21, 133.63, 131.37, 130.47, 130.18, 129.93, 127.06, 126.89, 126.85, 125.10, 120.82.

HRMS (ESI-ToF): calc. for $[C_{94}H_{62}FeN_6]^{2+}$ 665.2188; found 665.2189.

^{1}H-, ^{13}C-NMR ($CDCl_3$, 400/101 MHz, 22 °C) and HR-ESI-MS spectra of compound 4

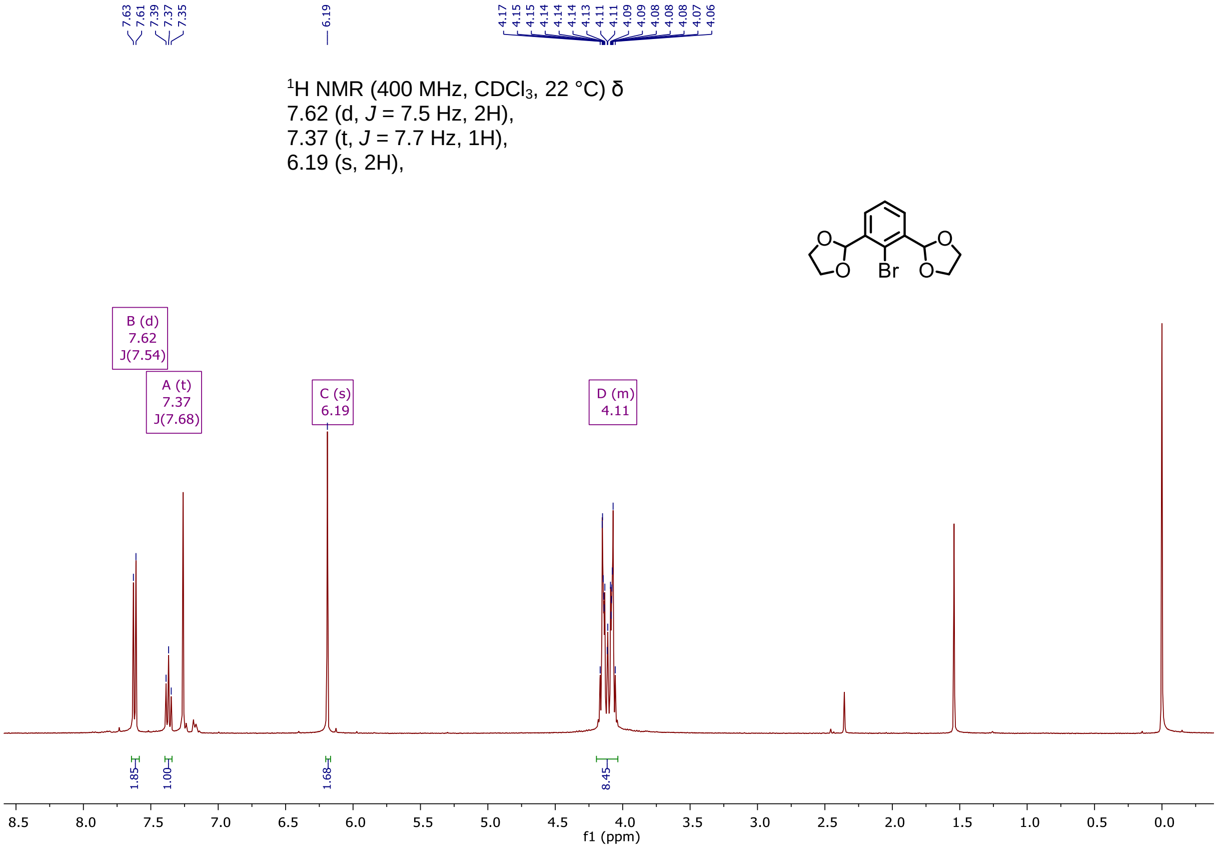

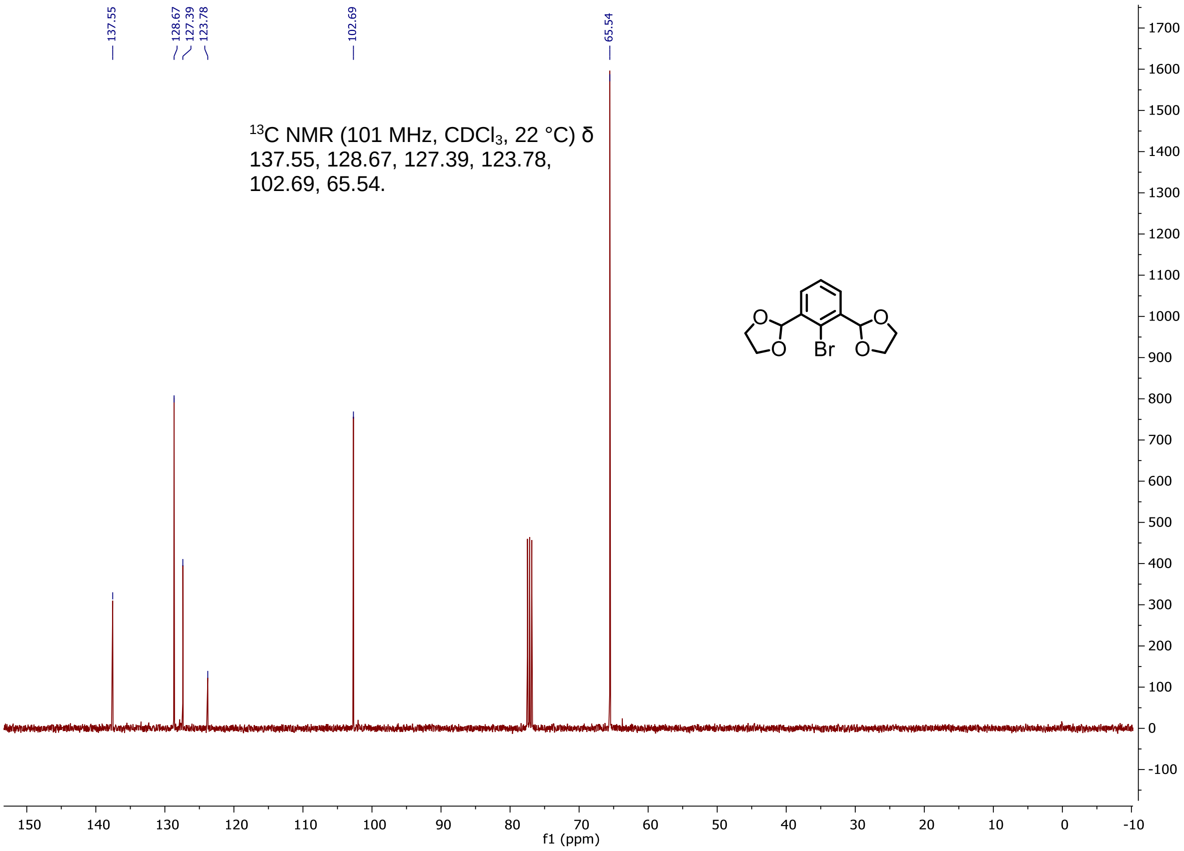

Mass Spectrum SmartFormula Report

Analysis Info

Analysis Name	D:\Data\acquisition data hn\BRT362.d	Acquisition Date	12.03.2019 09:08:45
Method	22 Direct_pos_mid.m	Operator	Miff
Sample Name	Thomas Brandl / BRT362	Instrument / Ser#	maXis 4G 21243
Comment	10 mg/mL in DCM, analyzed in MeOH		

Acquisition Parameter

Source Type	ESI	Ion Polarity	Positive	Set Nebulizer	0.4 Bar
Focus	Not active	Set Capillary	3600 V	Set Dry Heater	180 °C
Scan Begin	75 m/z	Set End Plate Offset	-500 V	Set Dry Gas	4.0 l/min
Scan End	1700 m/z	Collision Energy	8.0 eV	Set Ion Energy (MS only)	4.0 eV

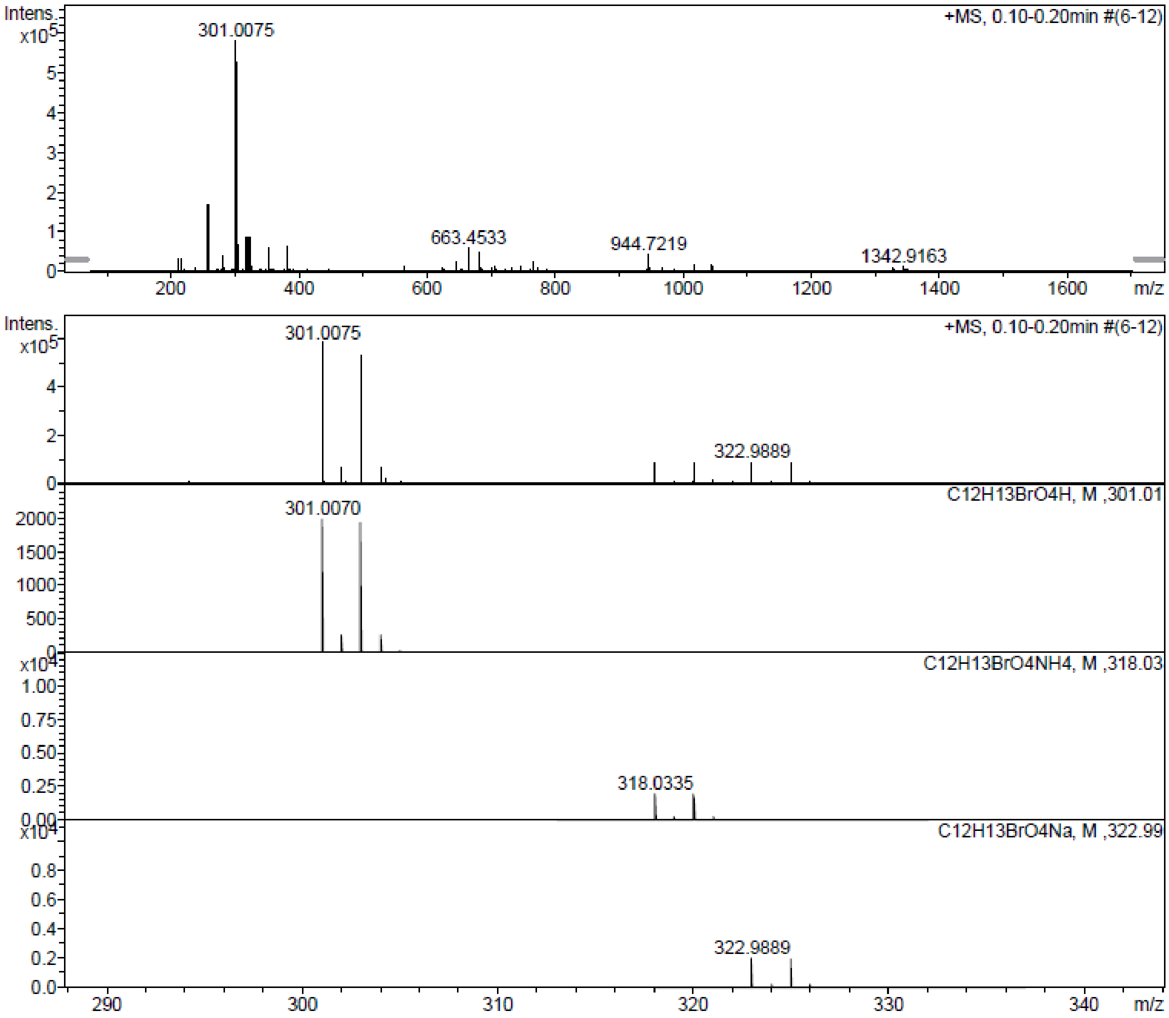

Meas. m/z	#	Formula	Score	m/z	err [mDa]	err [ppm]	mSigma	rdb	e⁻ Conf	z
301.0075	1	C 12 H 14 Br O 4	100.00	301.0070	-0.5	-1.7	37.1	5.5	even	1+
318.0335	1	C 12 H 17 Br N O 4	100.00	318.0335	-0.0	-0.0	5.3	4.5	even	
322.9889	1	C 12 H 13 Br Na O 4	100.00	322.9889	-0.0	-0.0	12.9	5.5	even	

^{1}H-, ^{13}C-NMR ($CDCl_3$, 400/101 MHz, 22 °C) and HR-ESI-MS spectra of compound 5

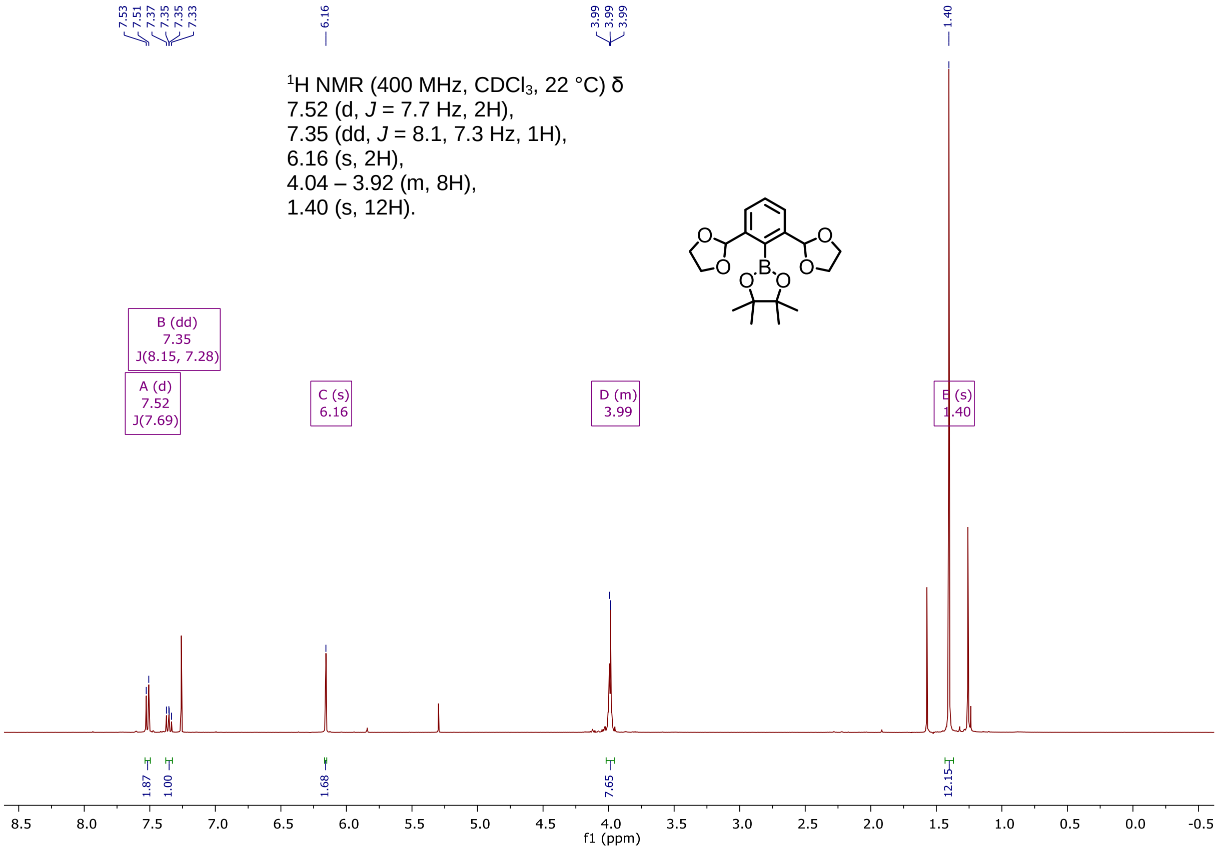

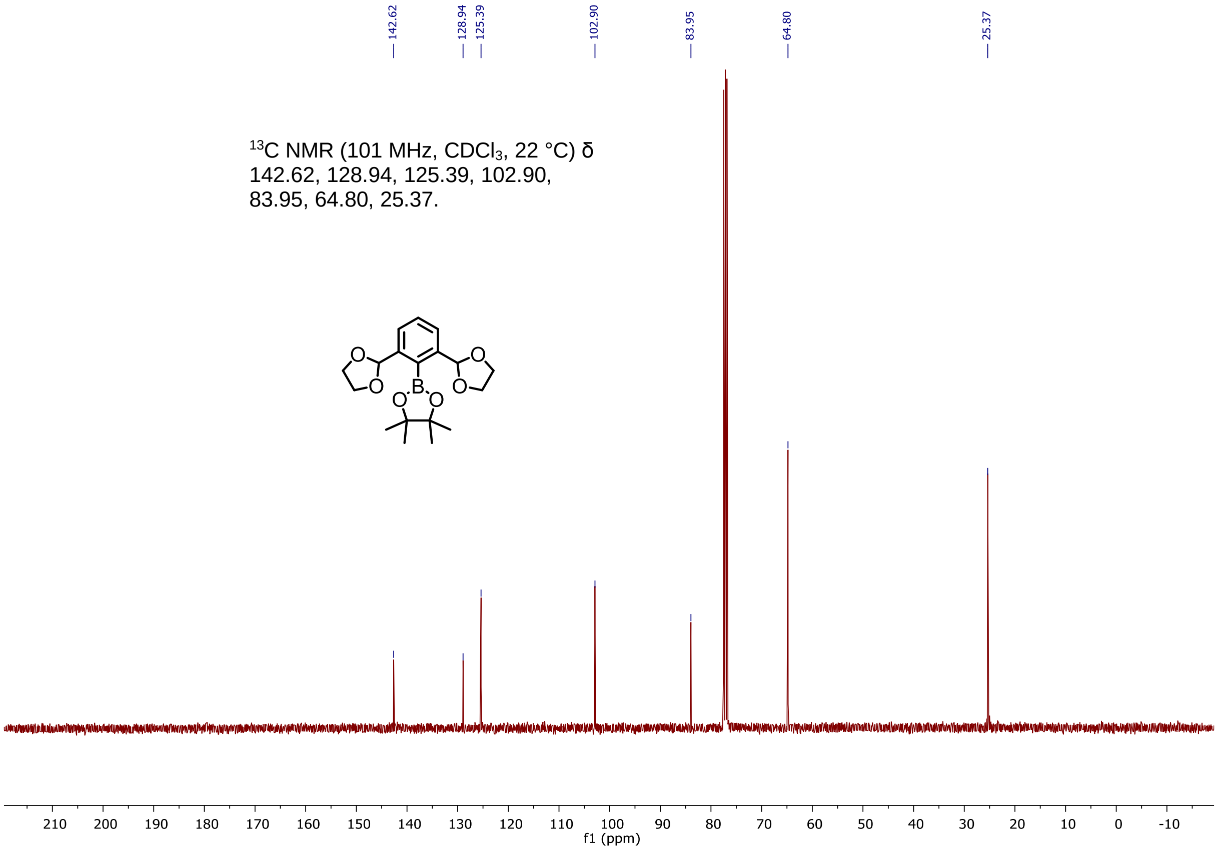

Mass Spectrum SmartFormula Report

Analysis Info

Analysis Name	N:\new acq data\BRT099 001.d	Acquisition Date	04.11.2015 15:31:23
Method	hn Direct_Infusion_pos mode_75-1700 mid 4eV.m	Operator	hn
Sample Name	Thomas Brandl	Instrument / Ser#	maXis 4G 21243
Comment	BRT099, ca. 10 ug/ml MeCN, 1:10 verdünnt, mit HCOOH		

Acquisition Parameter

Source Type	ESI	Ion Polarity	Positive	Set Nebulizer	0.4 Bar
Focus	Not active	Set Capillary	3600 V	Set Dry Heater	180 °C
Scan Begin	75 m/z	Set End Plate Offset	-500 V	Set Dry Gas	4.0 l/min
Scan End	1700 m/z	Set Collision Cell RF	350.0 Vpp	Set Ion Energy (MS only)	4.0 eV

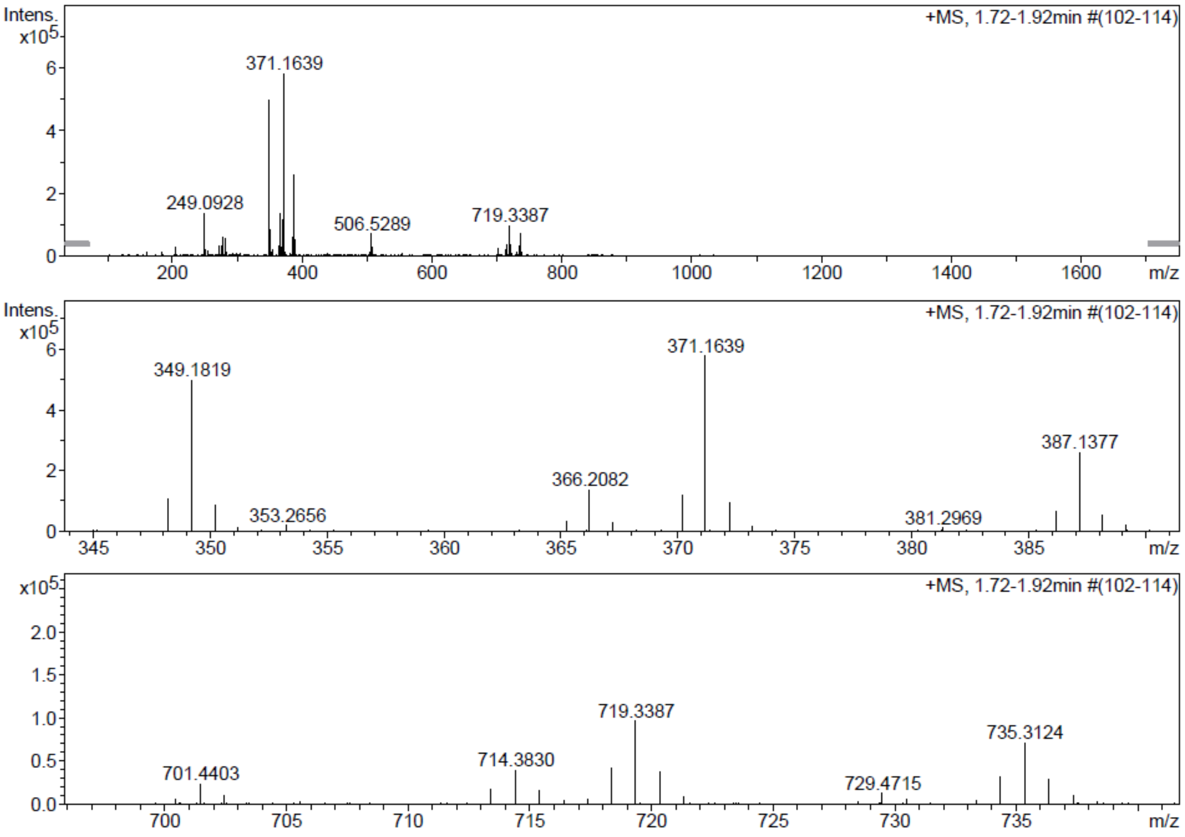

Meas. m/z	#	Formula	Score	m/z	err [mDa]	err [ppm]	mSigma	rdb	e⁻ Conf	N-Rule	z
349.1819	1	C 18 H 26 B O 6	100.00	349.1820	0.1	0.2	17.2	6.5	even	ok	1+
366.2082	1	C 18 H 29 B N O 6	100.00	366.2086	0.0	0.0	0.7	5.5	even	ok	
371.1639	1	C 18 H 25 B Na O 6	100.00	371.1640	0.1	0.1	24.8	6.5	even	ok	
387.1377	1	C 18 H 25 B K O 6	100.00	387.1379	-0.1	-0.3	12.4	6.5	even	ok	
714.3830	1	C 36 H 54 B 2 N O 12	100.00	714.3838	-0.4	-0.5	8.6	11.5	even	ok	
719.3387	1	C 36 H 50 B 2 Na O 12	100.00	719.3392	0.5	0.8	5.1	12.5	even	ok	
735.3124	1	C 36 H 50 B 2 K O 12	100.00	735.3132	-0.4	-0.6	8.5	12.5	even	ok	

^{1}H-, ^{13}C-NMR (CD_2Cl_2, 400/101 MHz, 22 °C) and HR-ESI-MS spectra of compound 6

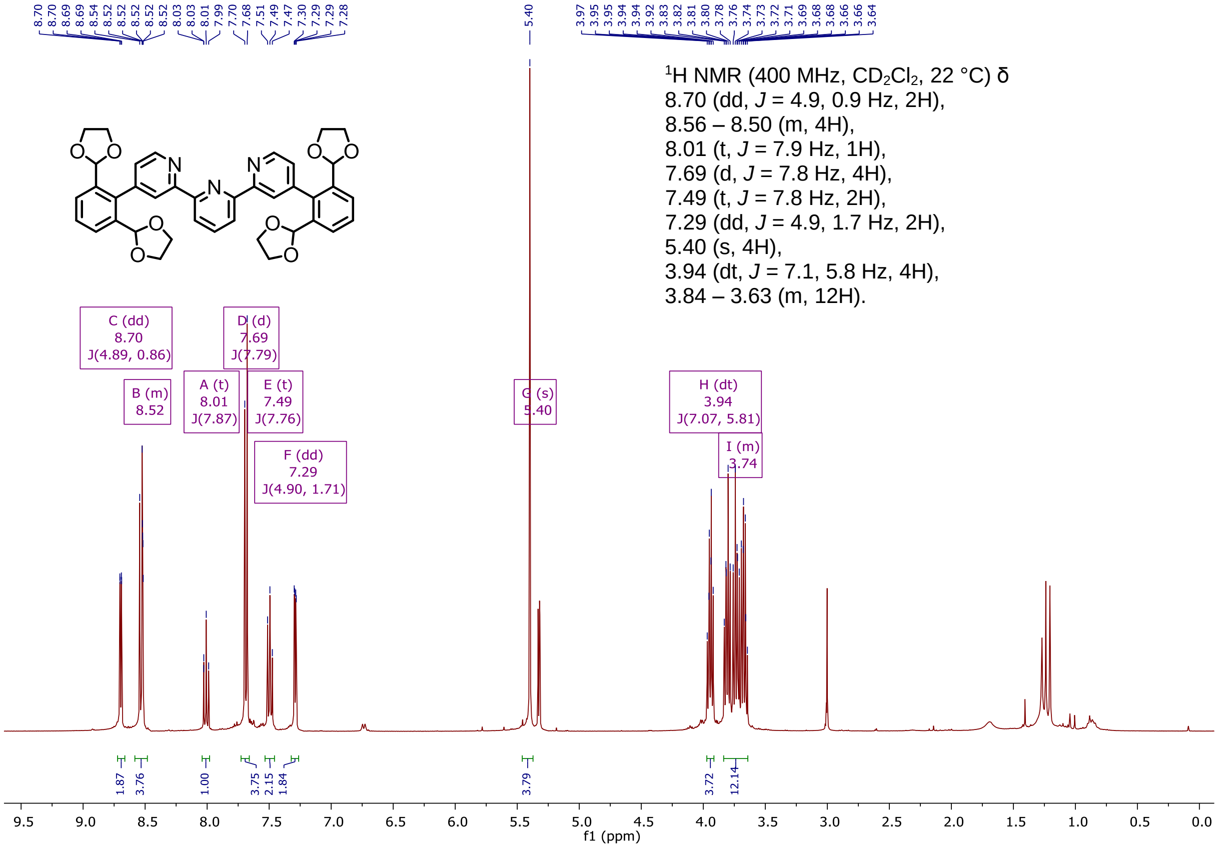

^{1}H NMR (400 MHz, CD_2Cl_2, 22 °C) δ
8.70 (dd, J = 4.9, 0.9 Hz, 2H),
8.56 – 8.50 (m, 4H),
8.01 (t, J = 7.9 Hz, 1H),
7.69 (d, J = 7.8 Hz, 4H),
7.49 (t, J = 7.8 Hz, 2H),
7.29 (dd, J = 4.9, 1.7 Hz, 2H),
5.40 (s, 4H),
3.94 (dt, J = 7.1, 5.8 Hz, 4H),
3.84 – 3.63 (m, 12H).

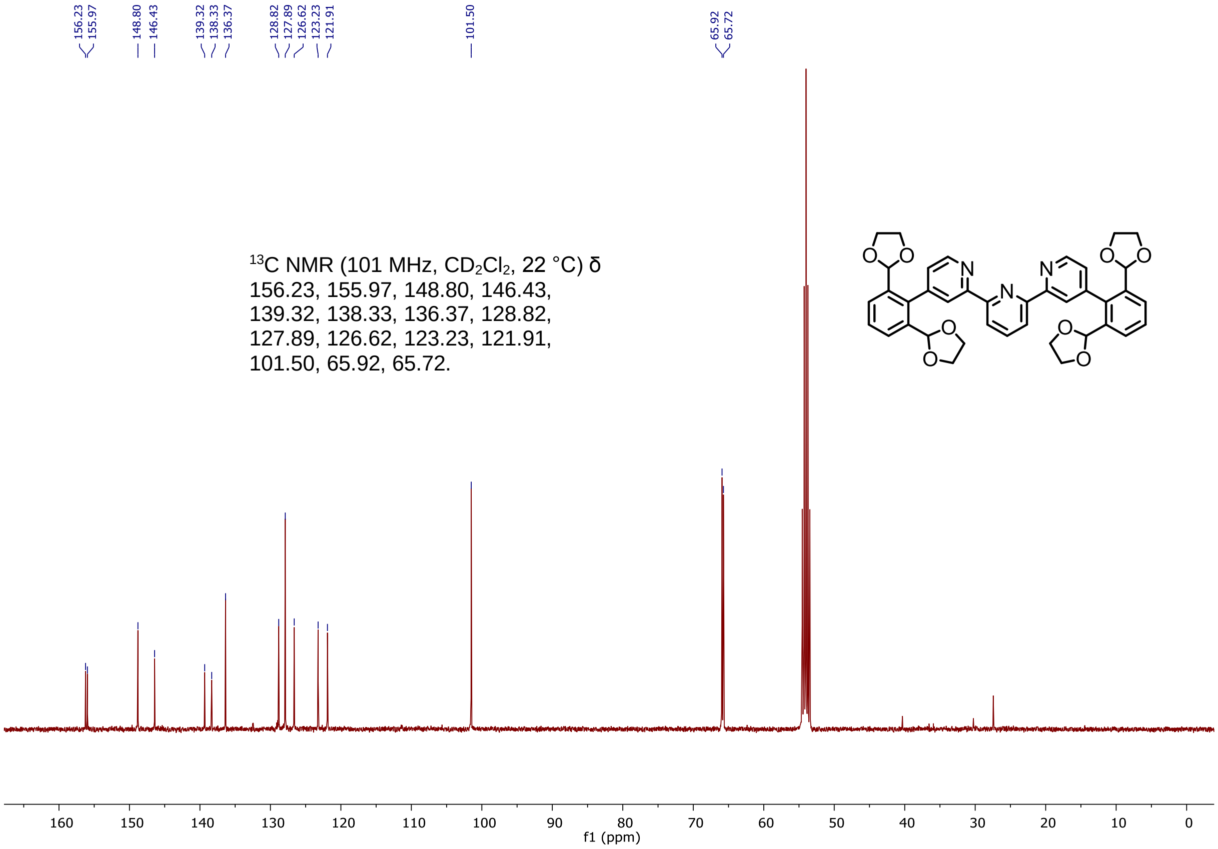

^{13}C NMR (101 MHz, CD_2Cl_2, 22 °C) δ
156.23, 155.97, 148.80, 146.43,
139.32, 138.33, 136.37, 128.82,
127.89, 126.62, 123.23, 121.91,
101.50, 65.92, 65.72.

Mass Spectrum SmartFormula Report

Analysis Info

Analysis Name	N:\new acq data\BRT078 001.d	Acquisition Date	08.05.2015 17:03:23
Method	hn Direct_Infusion_pos mode_75-1700 mid 4eV.m	Operator	hn
Sample Name	Thomas Brandl, BRT078	Instrument / Ser#	maXis 4G 21243
Comment	BRT078, ca. 50 ug/ml MeCN, 1:10 verdünnt		

Acquisition Parameter

Source Type	ESI	Ion Polarity	Positive	Set Nebulizer	0.4 Bar
Focus	Not active	Set Capillary	3600 V	Set Dry Heater	180 °C
Scan Begin	75 m/z	Set End Plate Offset	-500 V	Set Dry Gas	4.0 l/min
Scan End	1700 m/z	Set Collision Cell RF	500.0 Vpp	Set Ion Energy (MS only)	4.0 eV

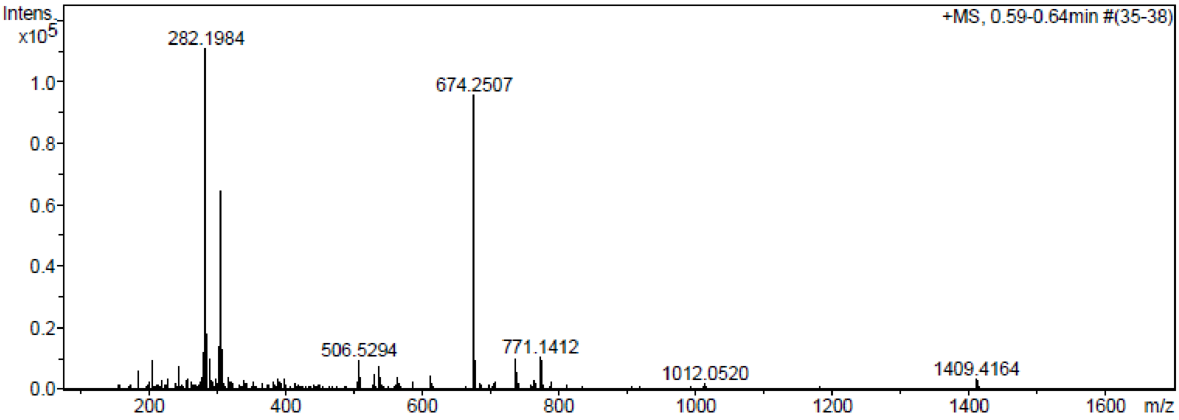

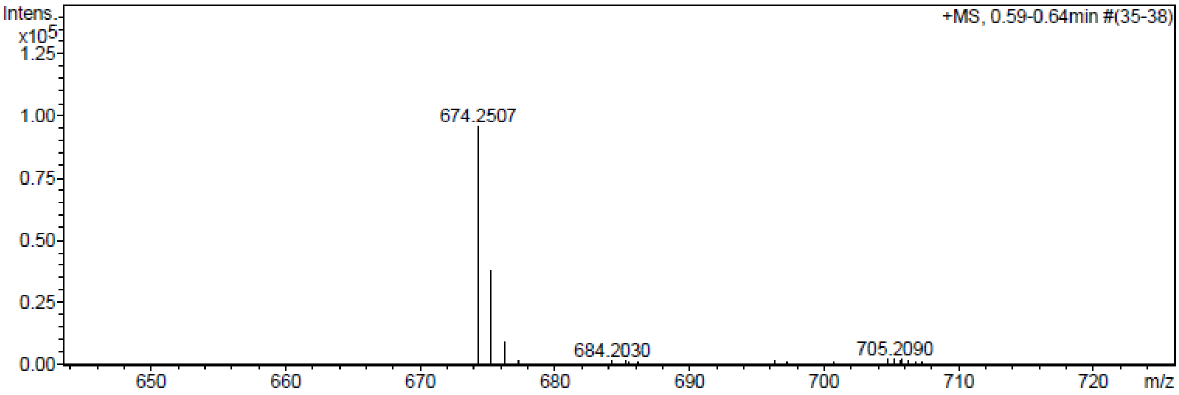

Meas. m/z	#	Formula	Score	m/z	err [mDa]	err [ppm]	mSigma	rdb	e⁻ Conf	N-Rule	z
674.2507	1	C 39 H 36 N 3 O 8	100.00	674.2497	-1.0	-1.5	23.4	23.5	even	ok	1+

^{1}H-, ^{13}C-NMR (CD_2Cl_2, 400/101 MHz, 22 °C) and HR-ESI-MS spectra of compound 7

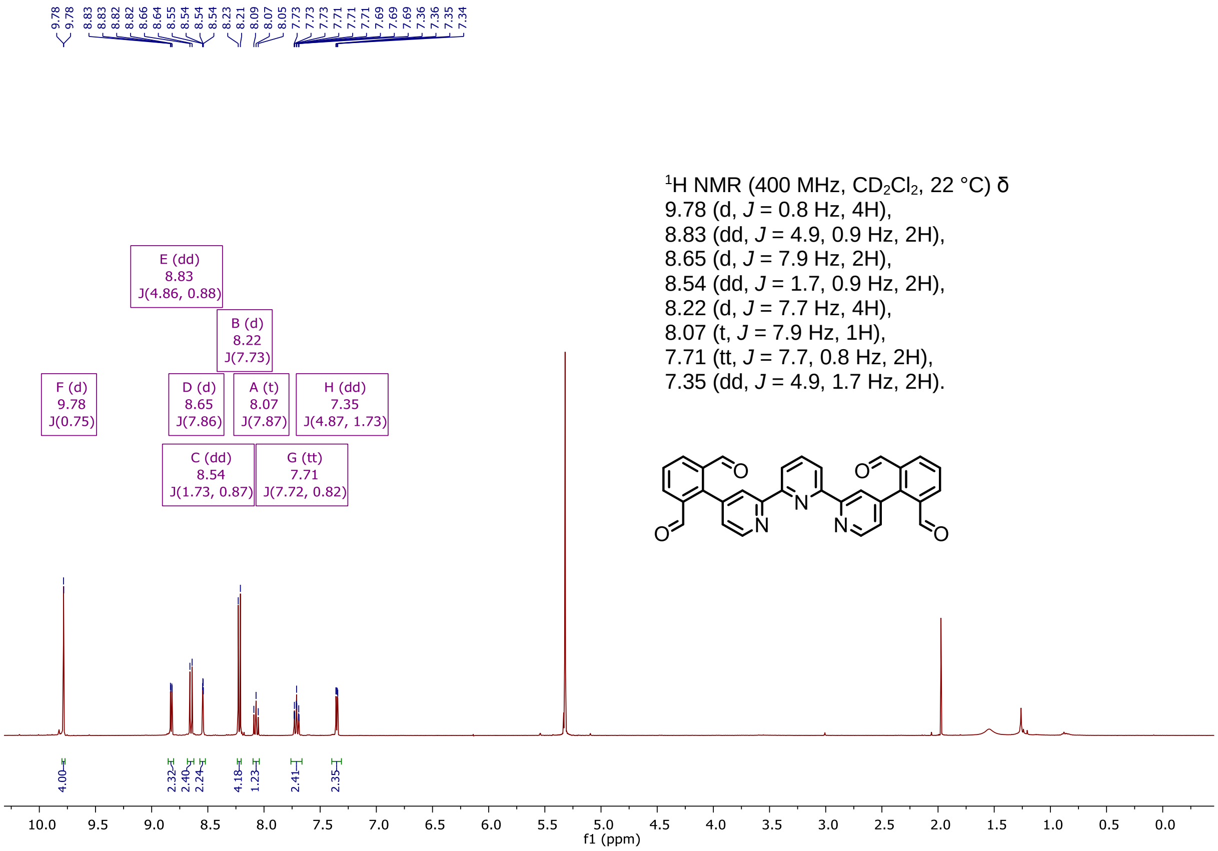

^{1}H NMR (400 MHz, CD_2Cl_2, 22 °C) δ
9.78 (d, J = 0.8 Hz, 4H),
8.83 (dd, J = 4.9, 0.9 Hz, 2H),
8.65 (d, J = 7.9 Hz, 2H),
8.54 (dd, J = 1.7, 0.9 Hz, 2H),
8.22 (d, J = 7.7 Hz, 4H),
8.07 (t, J = 7.9 Hz, 1H),
7.71 (tt, J = 7.7, 0.8 Hz, 2H),
7.35 (dd, J = 4.9, 1.7 Hz, 2H).

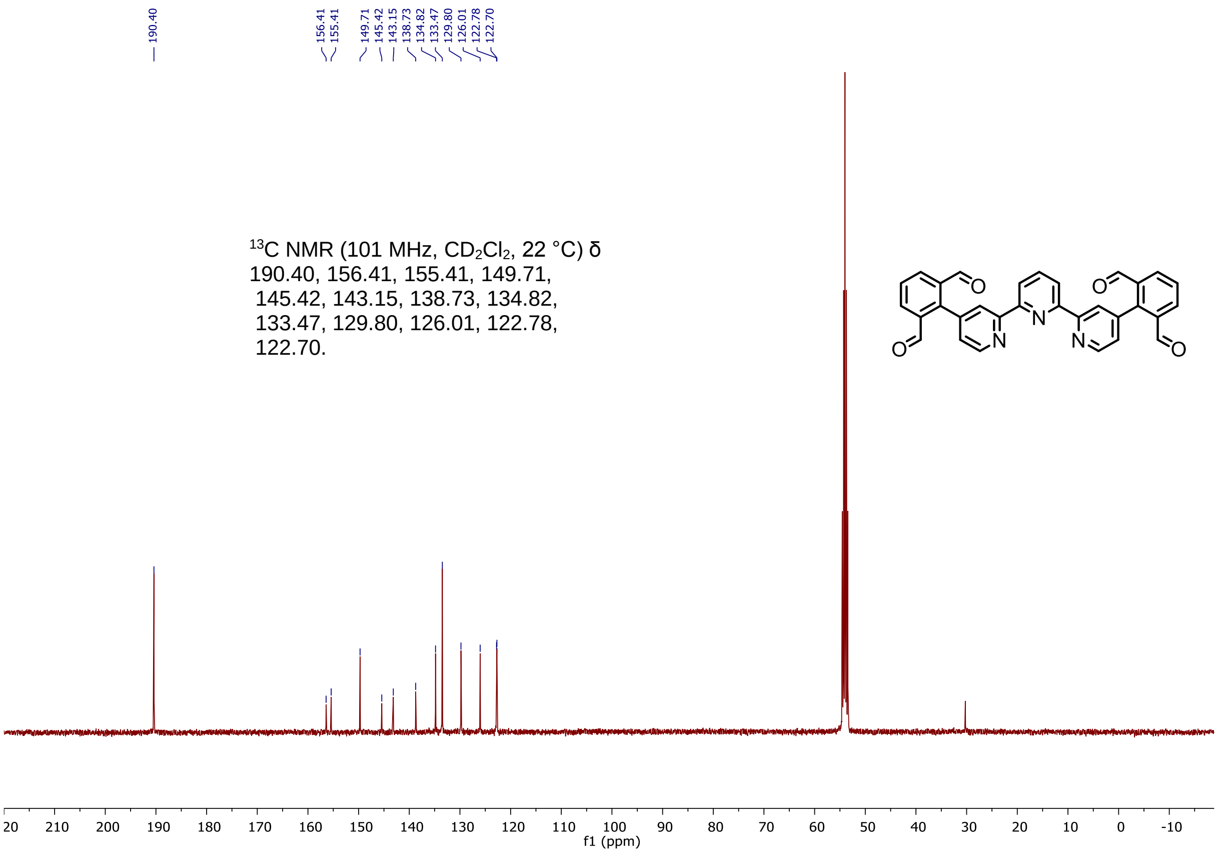

^{13}C NMR (101 MHz, CD_2Cl_2, 22 °C) δ
190.40, 156.41, 155.41, 149.71,
145.42, 143.15, 138.73, 134.82,
133.47, 129.80, 126.01, 122.78,
122.70.

Mass Spectrum SmartFormula Report

Analysis Info

Analysis Name	N:\new acq data\BRT080 002.d	Acquisition Date	12.05.2015 15:56:51
Method	hn Direct_Infusion_pos mode_75-1700 mid 4eV.m	Operator	hn
Sample Name	Thomas Brandl, BRT080	Instrument / Ser#	maXis 4G 21243
Comment	BRT080, ca. 10 ug/ml MeCN, via Spritze hinter MeOH/HCOOH		

Acquisition Parameter

Source Type	ESI	Ion Polarity	Positive	Set Nebulizer	0.4 Bar
Focus	Not active	Set Capillary	3600 V	Set Dry Heater	180 °C
Scan Begin	75 m/z	Set End Plate Offset	-500 V	Set Dry Gas	4.0 l/min
Scan End	1700 m/z	Set Collision Cell RF	500.0 Vpp	Set Ion Energy (MS only)	4.0 eV

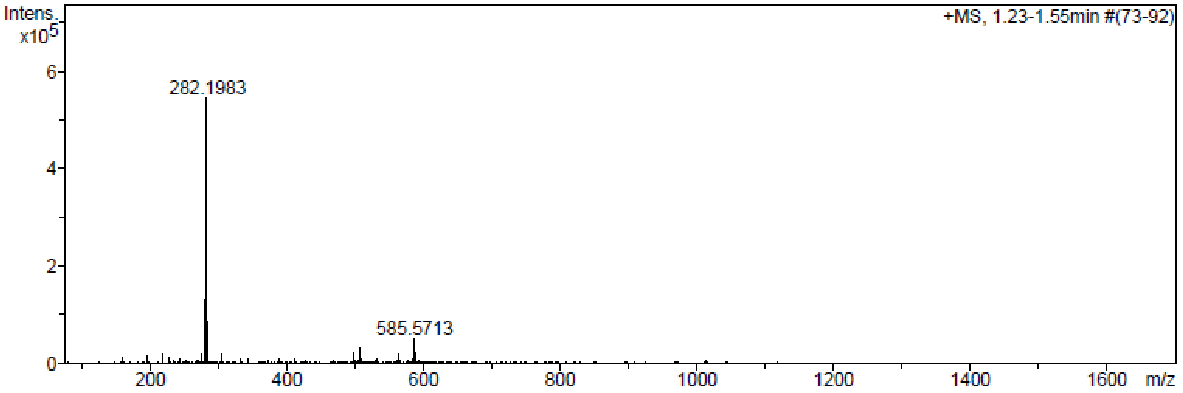

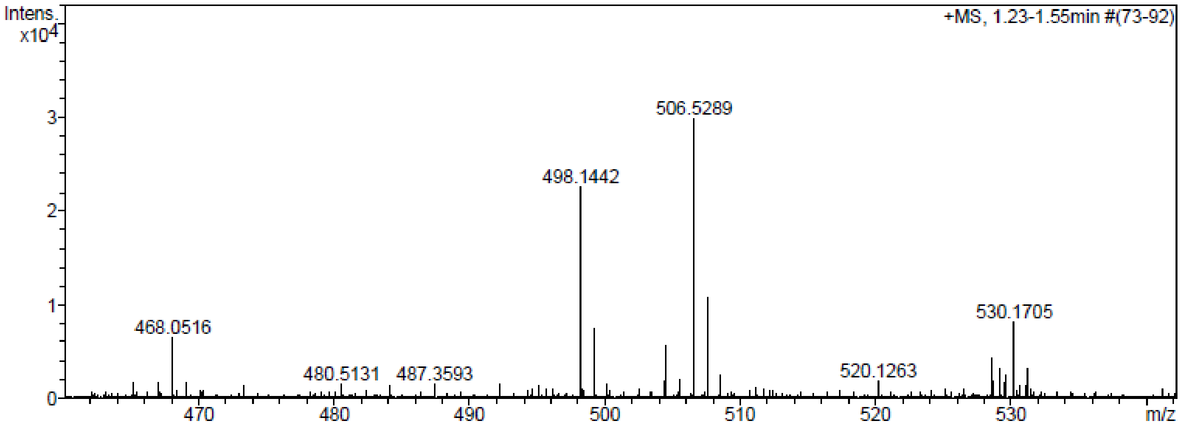

Meas. m/z	#	Formula	Score	m/z	err [mDa]	err [ppm]	mSigma	rdb	e⁻ Conf	N-Rule	z
498.1442	1	C 31 H 20 N 3 O 4	100.00	498.1448	0.6	1.2	6.7	23.5	even	ok	1+
520.1263	1	C 31 H 19 N 3 Na O 4	100.00	520.1268	0.4	0.8	32.1	23.5	even	ok	

Bruker Compass DataAnalysis 4.0 printed: 12.05.2015 17:01:55 Page 1 of 1

^{1}H-, ^{13}C-, COSY-, ROESY-, HSQC-, HMBC-NMR (Acetone-d$_6$/Acetonitrile-d$_3$, 600/400/151/101 MHz, 25 °C/22 °C) with full assignment and HR-ESI-MS spectra of compound 8

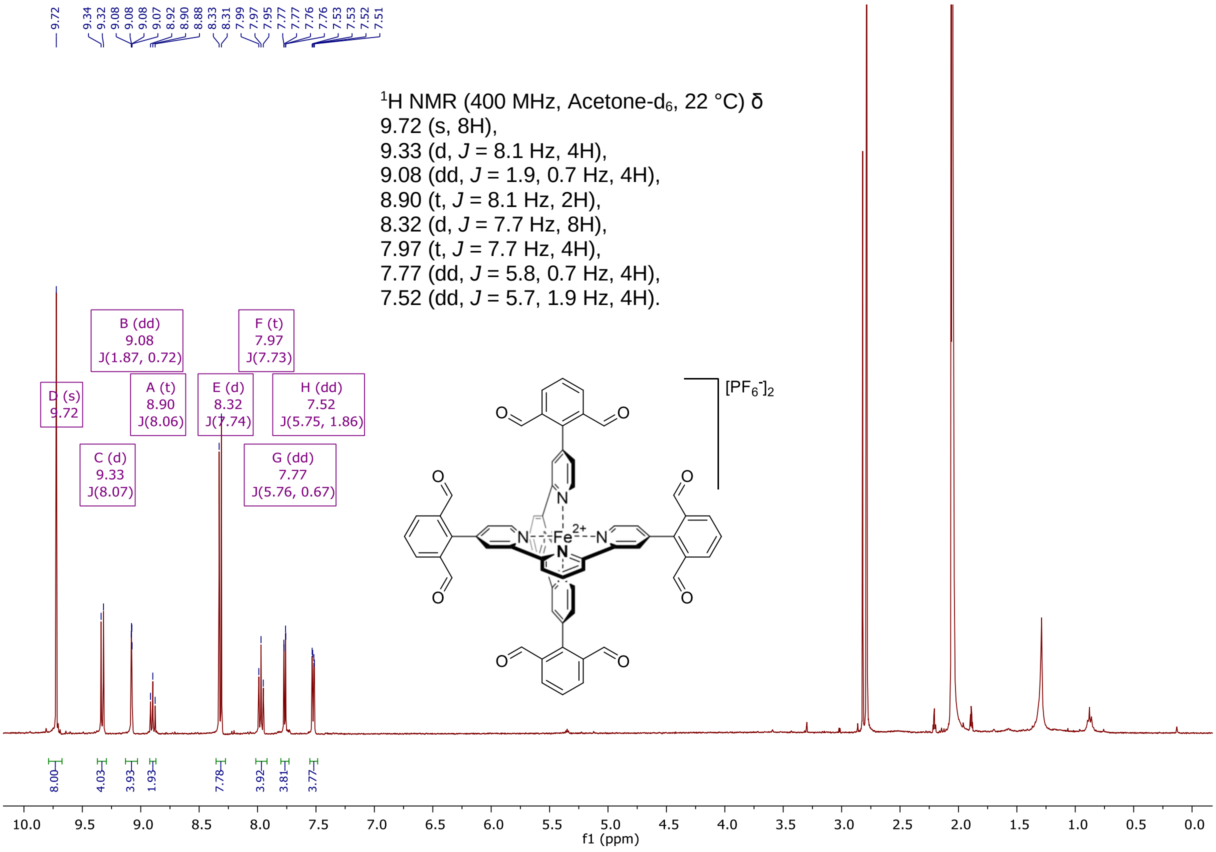

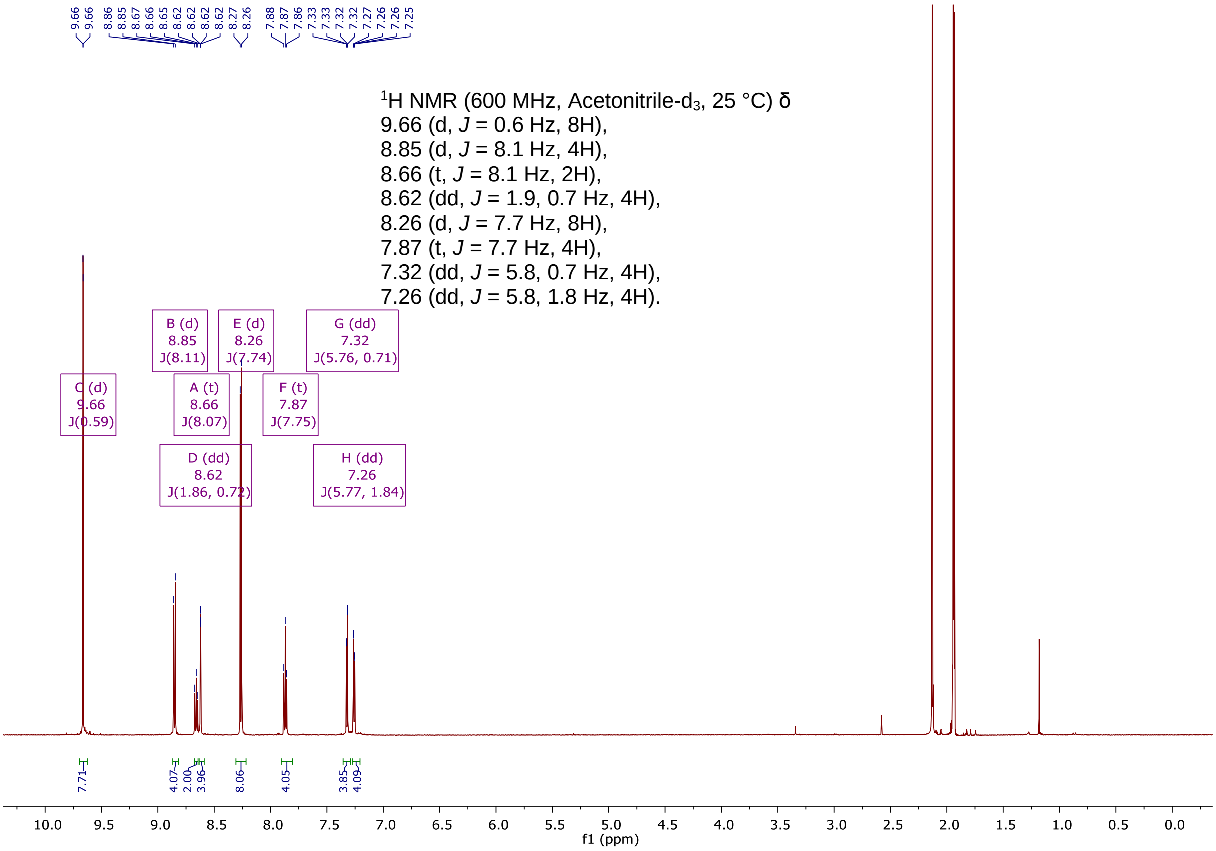

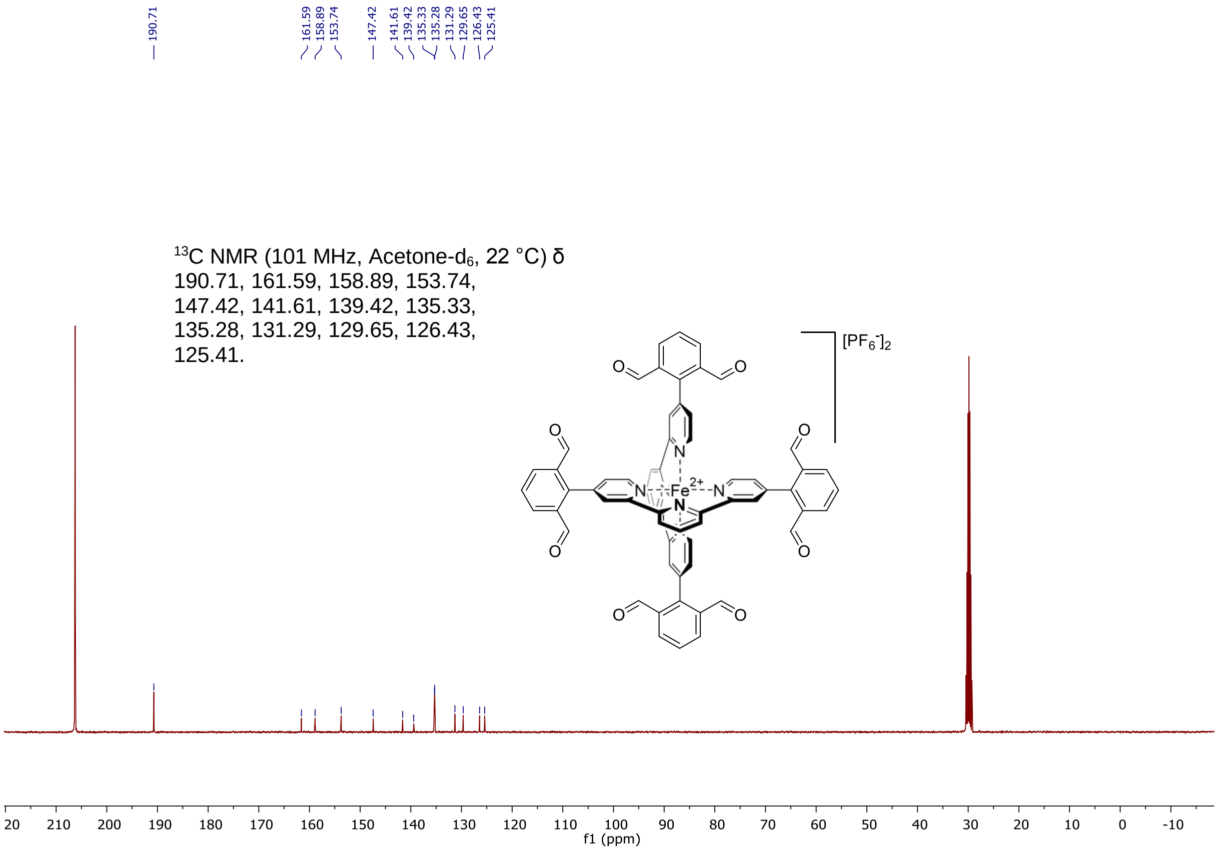

^{13}C NMR (101 MHz, Acetone-d_6, 22 °C) δ 190.71, 161.59, 158.89, 153.74, 147.42, 141.61, 139.42, 135.33, 135.28, 131.29, 129.65, 126.43, 125.41.

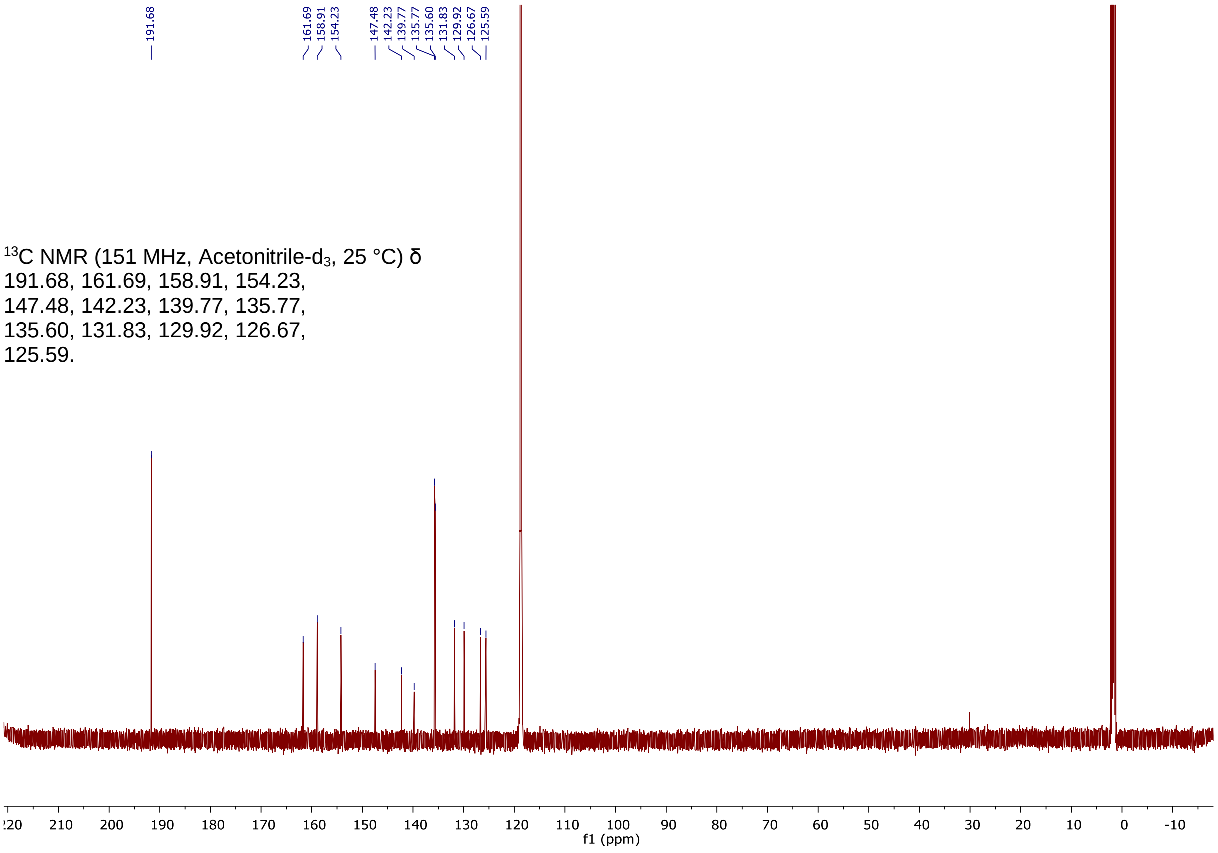

^{13}C NMR (151 MHz, Acetonitrile-d_3, 25 °C) δ 191.68, 161.69, 158.91, 154.23, 147.48, 142.23, 139.77, 135.77, 135.60, 131.83, 129.92, 126.67, 125.59.

COSY-NMR spectrum

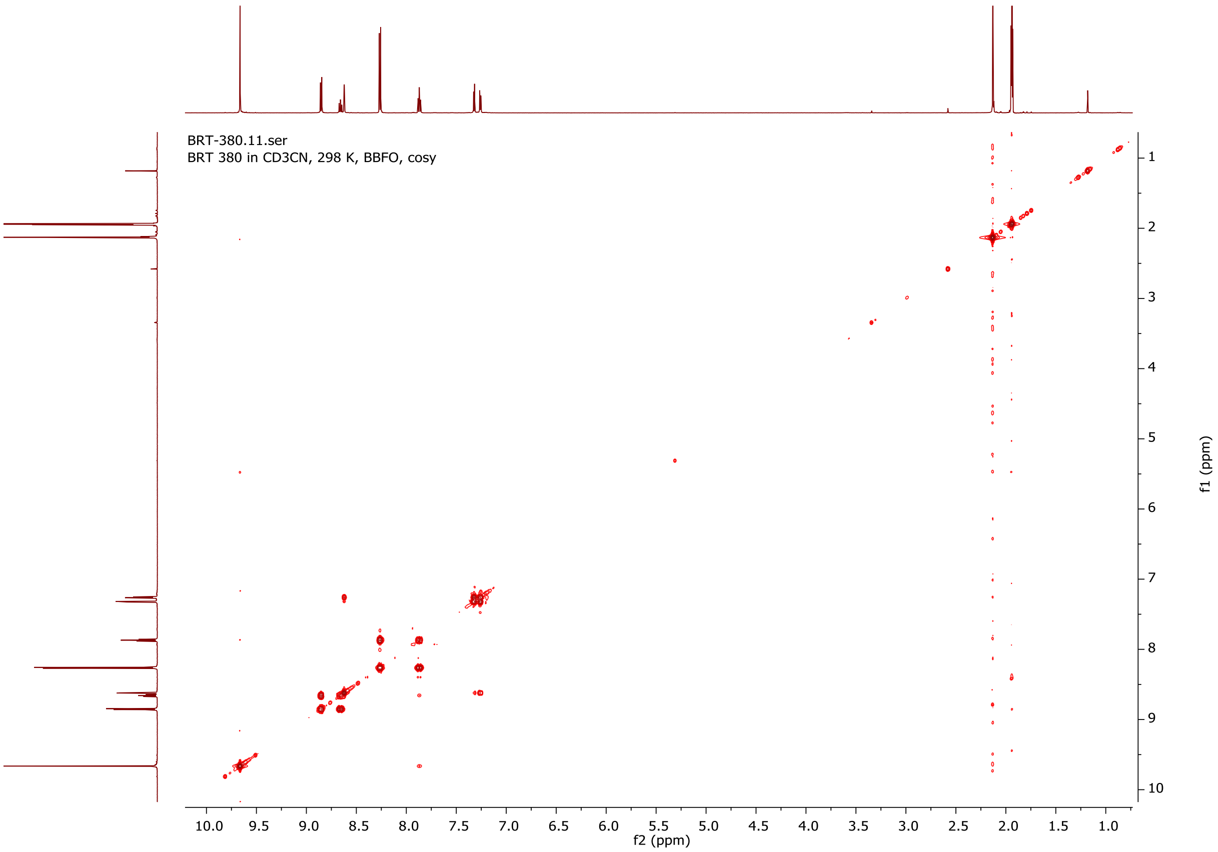

ROESY-NMR spectrum

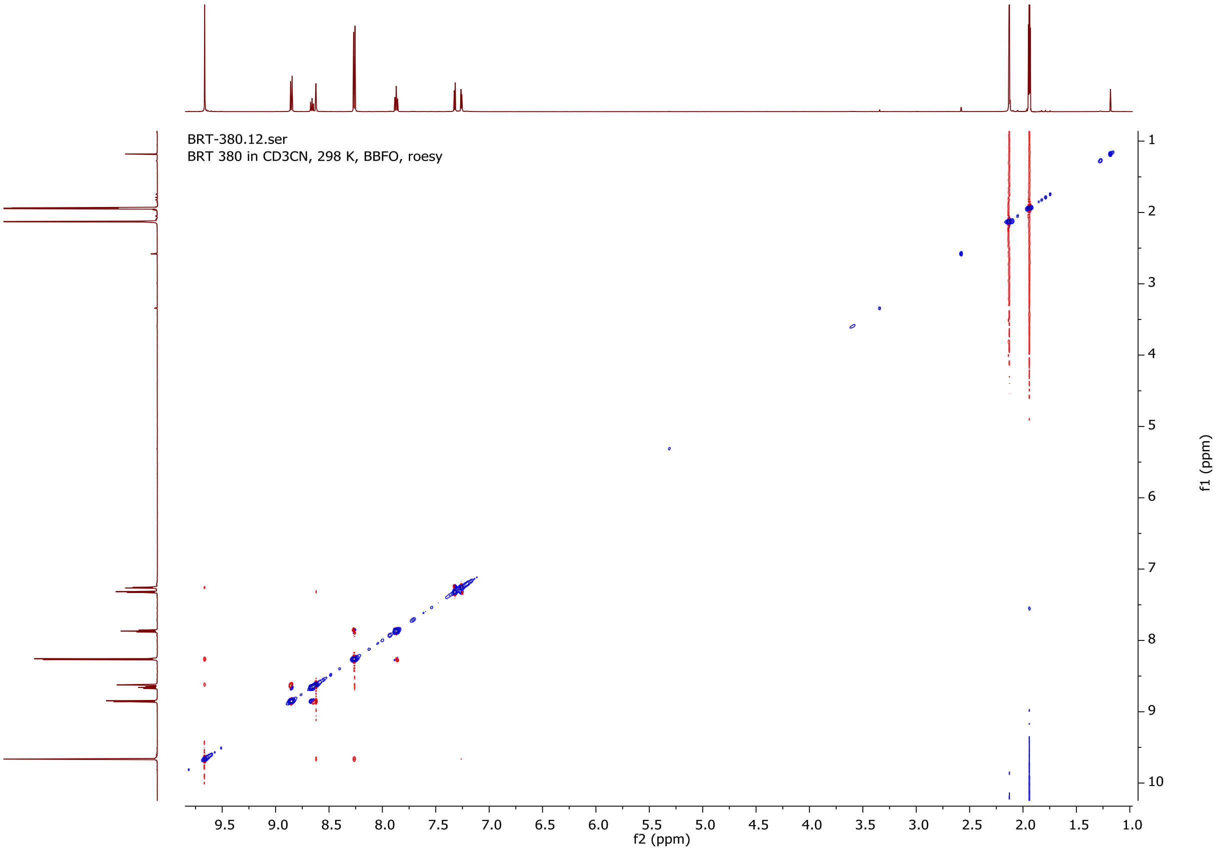

HSQC-NMR spectrum

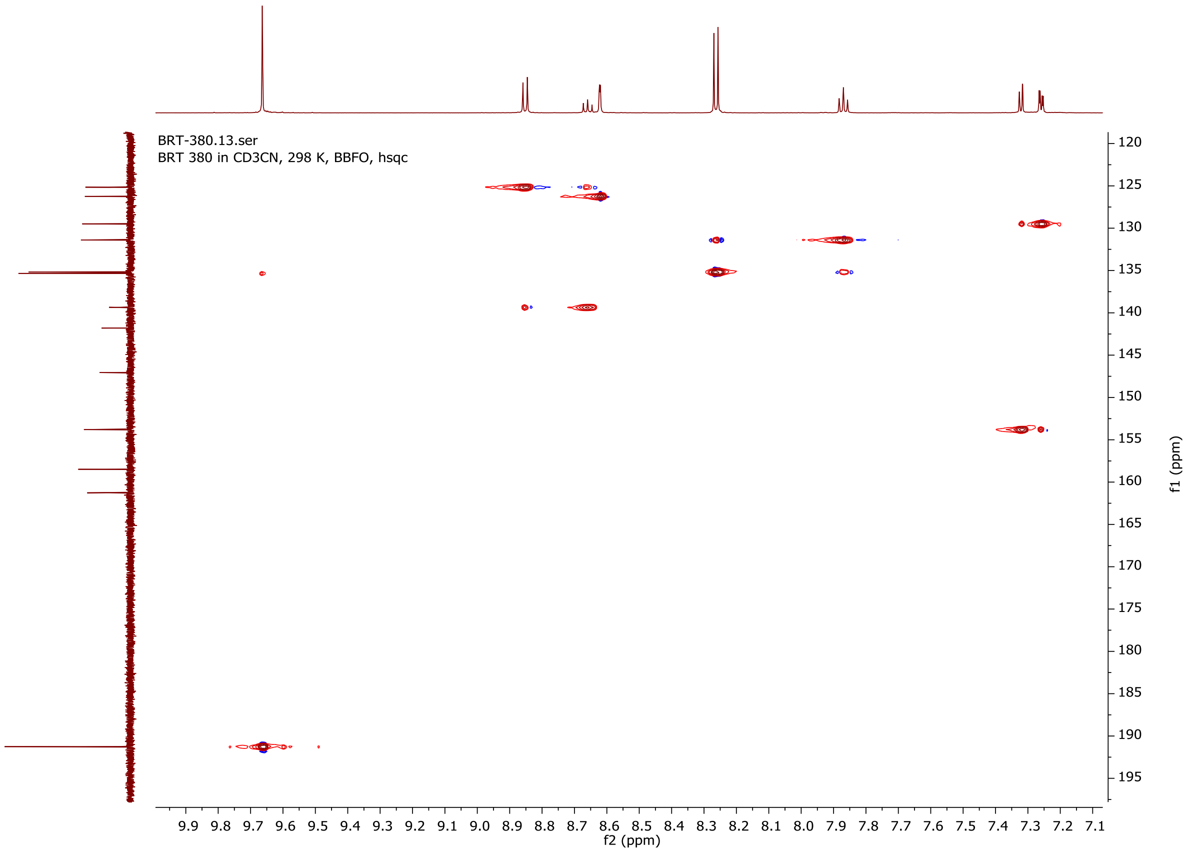

HMBC-NMR spectrum

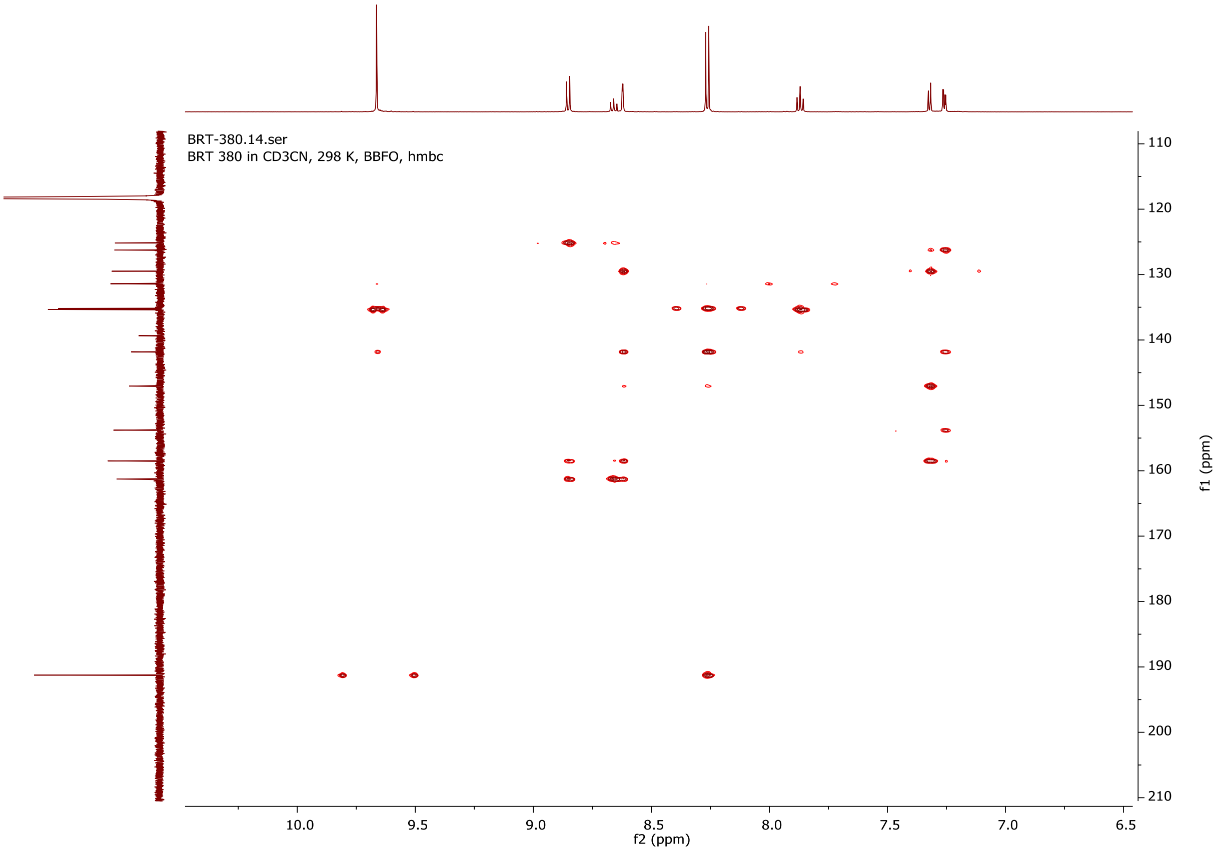

Full assignment of 8

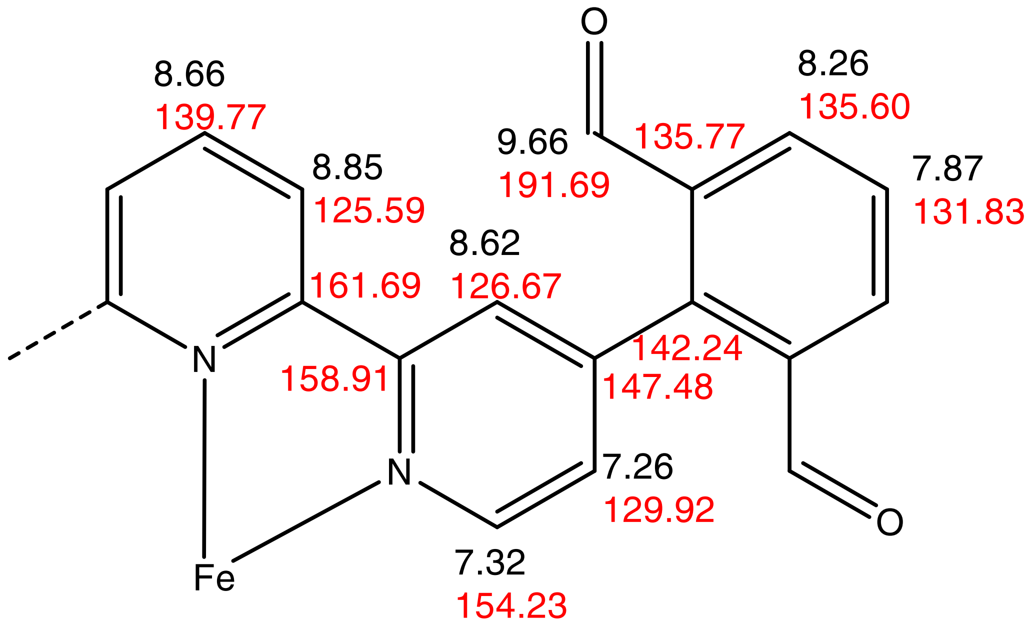

Figure SI1: Full-assignment of compound **8** in acetonitrile-d$_3$, 298 K, 600 MHz, BBFO.

Mass Spectrum SmartFormula Report

Analysis Info

Analysis Name	N:\new acq data\BRT083 001.d	Acquisition Date	19.05.2015 15:43:06
Method	hn Direct_Infusion_pos mode_75-1700 mid 4eV.m	Operator	hn
Sample Name	Thomas Brandl, BRT083	Instrument / Ser#	maXis 4G 21243
Comment	BRT083, ca. 10 ug/ml MeCN		

Acquisition Parameter

Source Type	ESI	Ion Polarity	Positive	Set Nebulizer	0.4 Bar
Focus	Not active	Set Capillary	3600 V	Set Dry Heater	180 °C
Scan Begin	75 m/z	Set End Plate Offset	-500 V	Set Dry Gas	4.0 l/min
Scan End	1700 m/z	Set Collision Cell RF	500.0 Vpp	Set Ion Energy (MS only)	4.0 eV

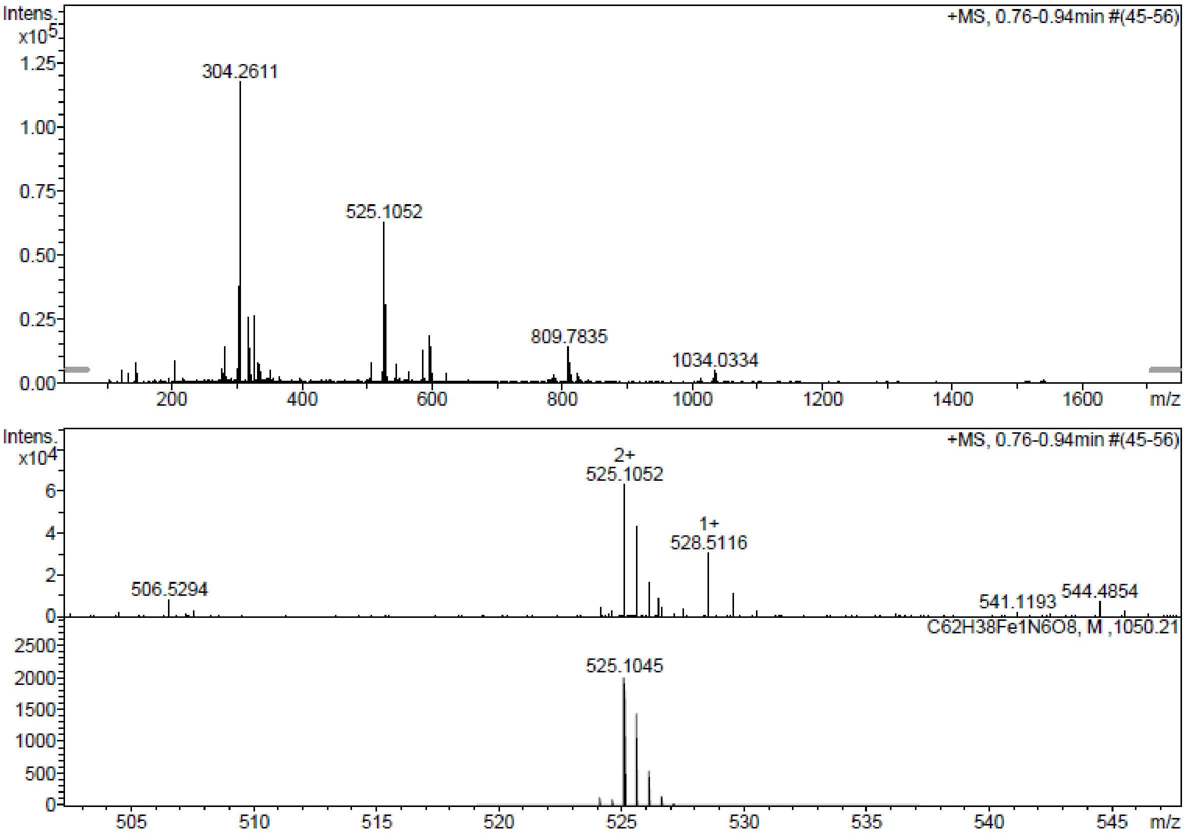

Meas. m/z	#	Formula	Score	m/z	err [mDa]	err [ppm]	mSigma	rdb	e⁻ Conf	N-Rule	z
525.1052	1	C 62 H 38 Fe N 6 O 8	100.00	525.1046	-0.6	-1.2	12.9	47.0	even	ok	2+

Mass Spectrum SmartFormula Report

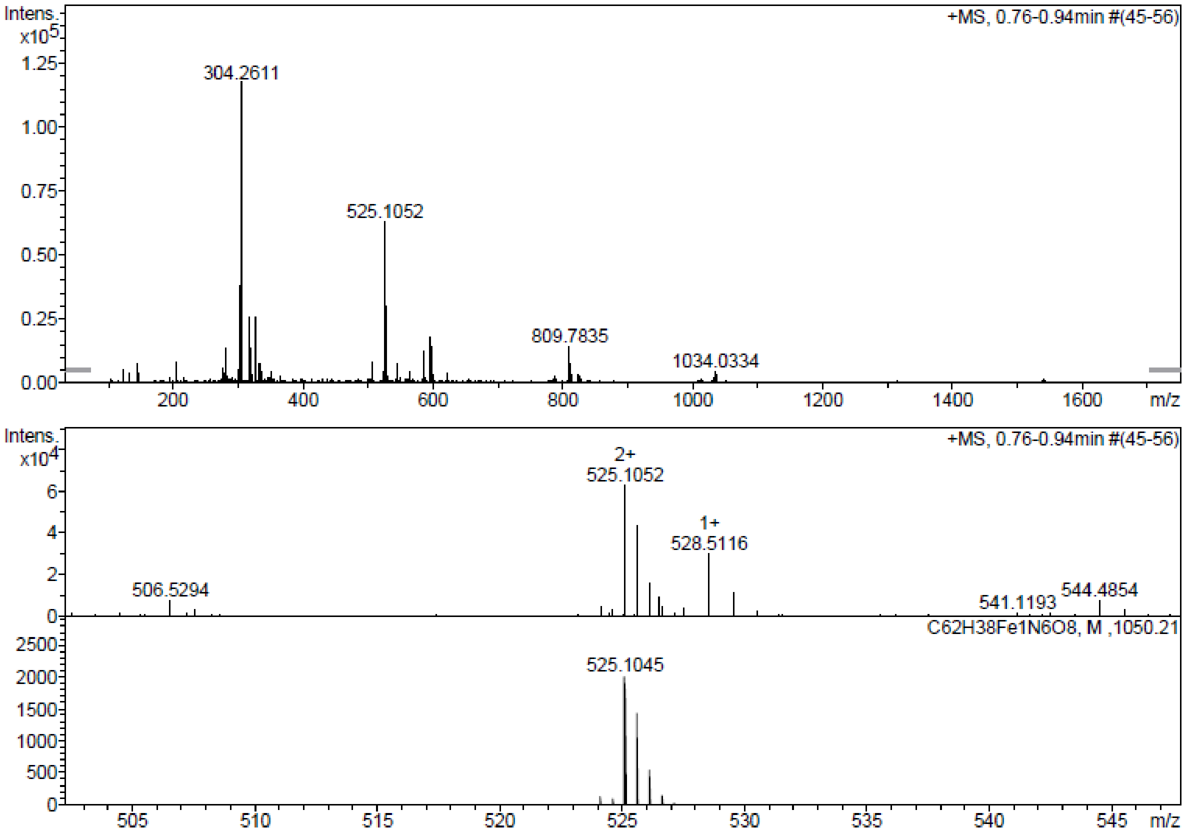

Meas. m/z	#	Formula	Score	m/z	err [mDa]	err [ppm]	mSigma	rdb	e⁻ Conf	N-Rule	z

HR-ESI-MS spectra of compound 2

Mass Spectrum SmartFormula Report

Analysis Info

Analysis Name	N:\new acq data\BRT093 003.d	Acquisition Date	15.06.2015 17:04:49
Method	hn Direct_Infusion_pos mode_75-2000 higher 4eV.m	Operator	hn
Sample Name	Thomas Brandl, BRT093	Instrument / Ser#	maXis 4G 21243
Comment	ca. 10 ug/ml MeCN		

Acquisition Parameter

Source Type	ESI	Ion Polarity	Positive	Set Nebulizer	0.4 Bar
Focus	Not active	Set Capillary	3600 V	Set Dry Heater	180 °C
Scan Begin	75 m/z	Set End Plate Offset	-500 V	Set Dry Gas	4.0 l/min
Scan End	2000 m/z	Set Collision Cell RF	500.0 Vpp	Set Ion Energy (MS only)	4.0 eV

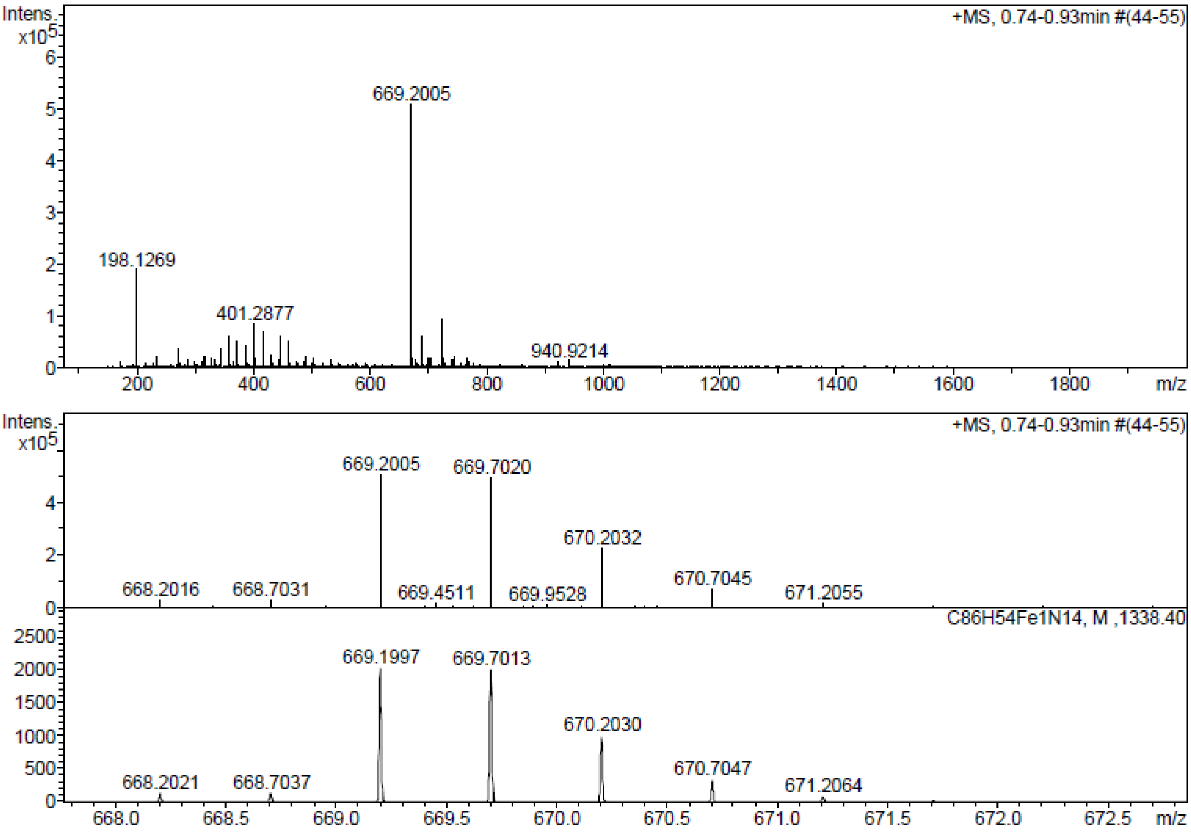

Meas. m/z	#	Formula	Score	m/z	err [mDa]	err [ppm]	mSigma	rdb	e⁻ Conf	N-Rule	z
669.2005	1	C 86 H 54 Fe N 14	100.00	669.1999	-0.6	-0.9	20.2	67.0	even	ok	2+

^{1}H-, ^{13}C-, COSY-, ROESY-, HSQC-, HMBC-NMR (Acetone-d$_6$, 600/151 MHz, 25 °C) with full assignment and HR-ESI-MS spectra of compound 1

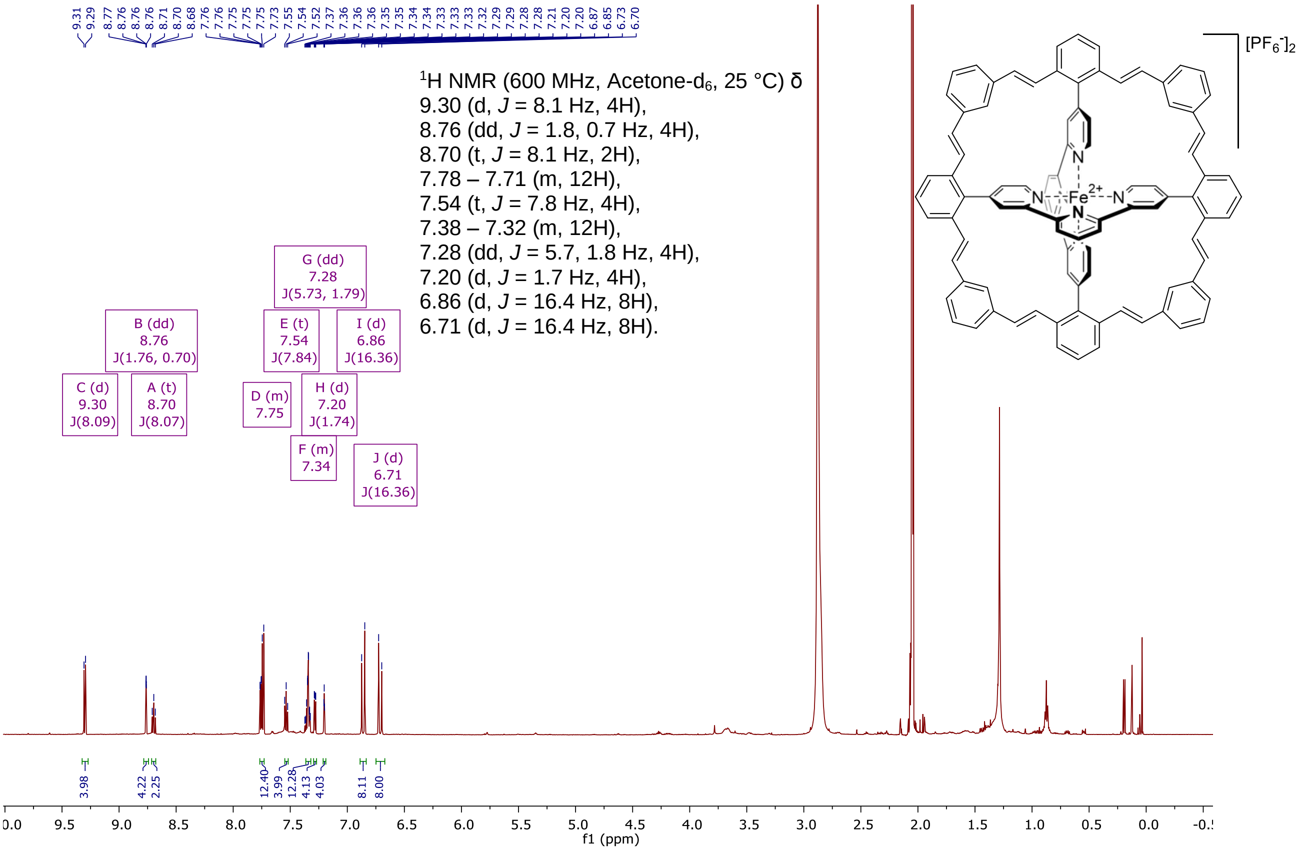

^{1}H NMR (600 MHz, Acetone-d$_6$, 25 °C) δ 9.30 (d, *J* = 8.1 Hz, 4H), 8.76 (dd, *J* = 1.8, 0.7 Hz, 4H), 8.70 (t, *J* = 8.1 Hz, 2H), 7.78 – 7.71 (m, 12H), 7.54 (t, *J* = 7.8 Hz, 4H), 7.38 – 7.32 (m, 12H), 7.28 (dd, *J* = 5.7, 1.8 Hz, 4H), 7.20 (d, *J* = 1.7 Hz, 4H), 6.86 (d, *J* = 16.4 Hz, 8H), 6.71 (d, *J* = 16.4 Hz, 8H).

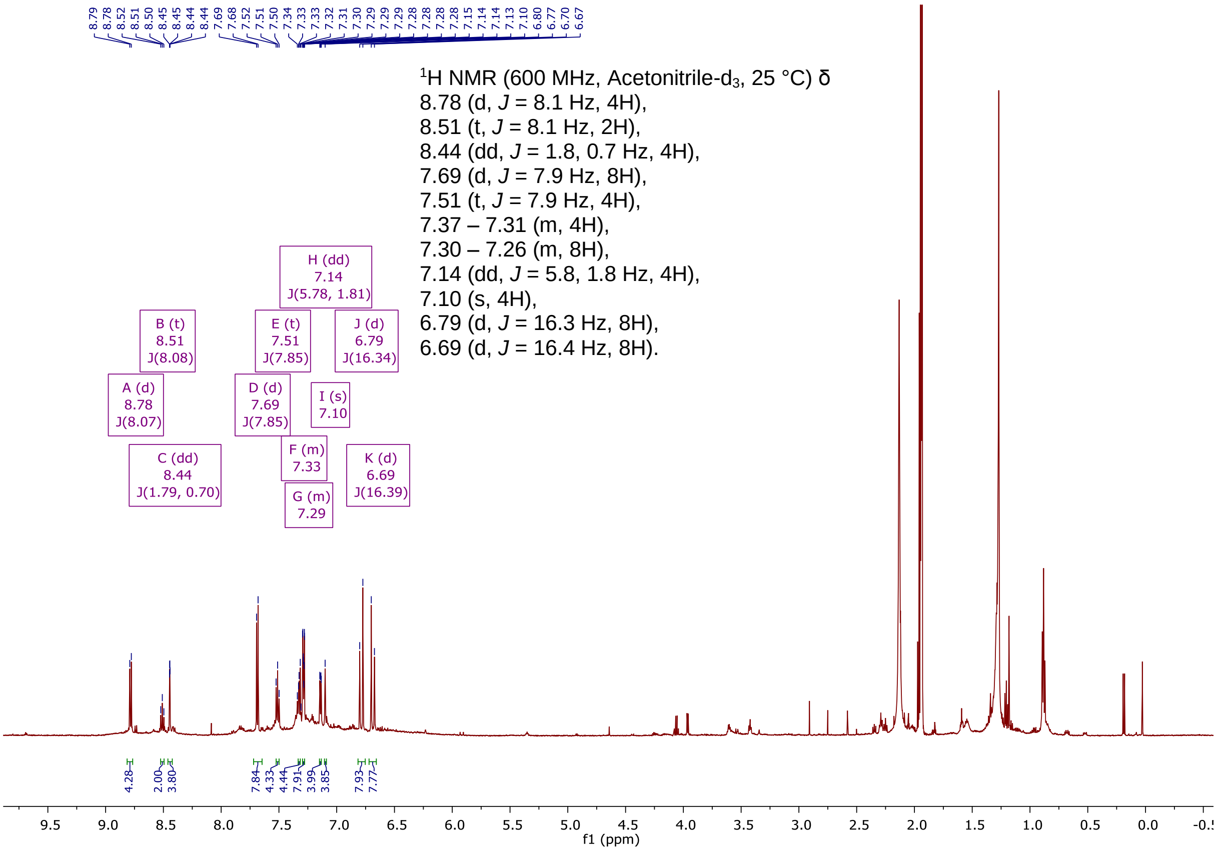

^{1}H NMR (600 MHz, Acetonitrile-d$_3$, 25 °C) δ 8.78 (d, *J* = 8.1 Hz, 4H), 8.51 (t, *J* = 8.1 Hz, 2H), 8.44 (dd, *J* = 1.8, 0.7 Hz, 4H), 7.69 (d, *J* = 7.9 Hz, 8H), 7.51 (t, *J* = 7.9 Hz, 4H), 7.37 – 7.31 (m, 4H), 7.30 – 7.26 (m, 8H), 7.14 (dd, *J* = 5.8, 1.8 Hz, 4H), 7.10 (s, 4H), 6.79 (d, *J* = 16.3 Hz, 8H), 6.69 (d, *J* = 16.4 Hz, 8H).

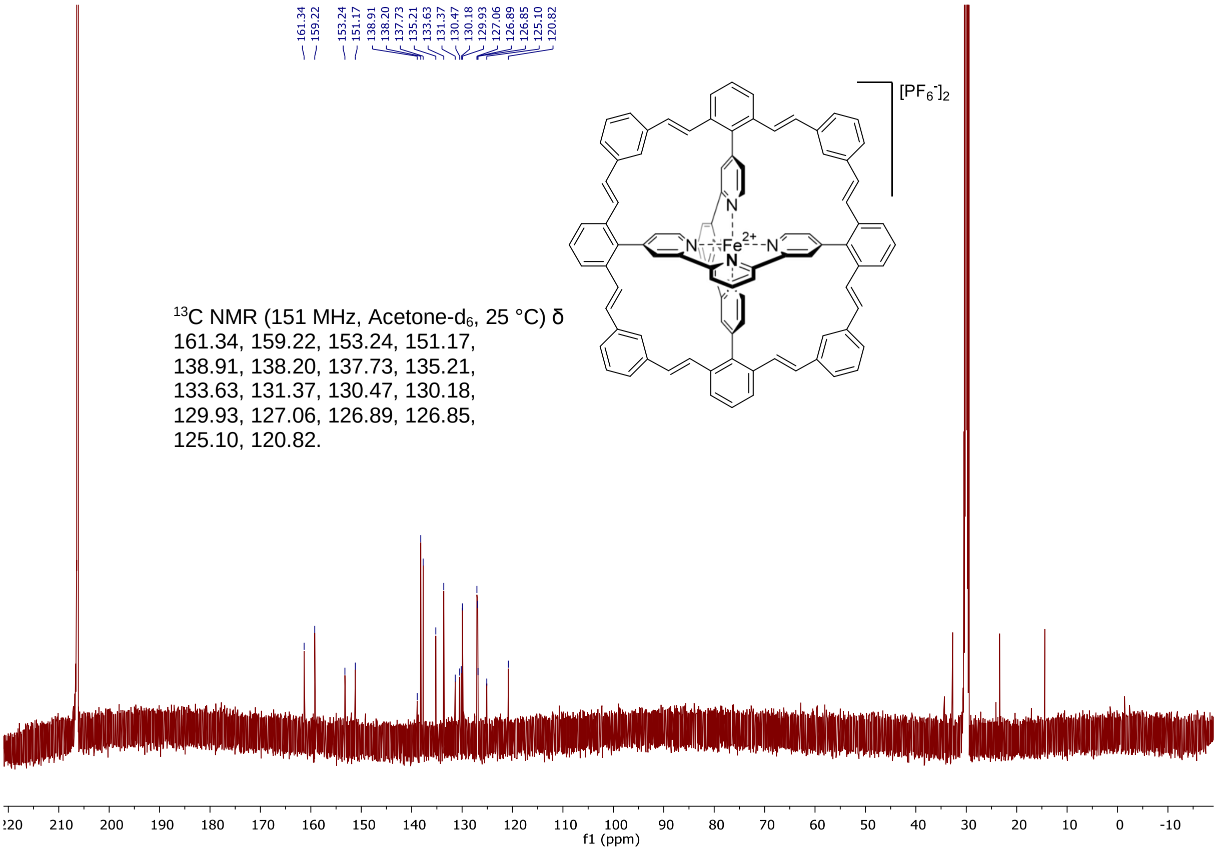

COSY-NMR spectrum

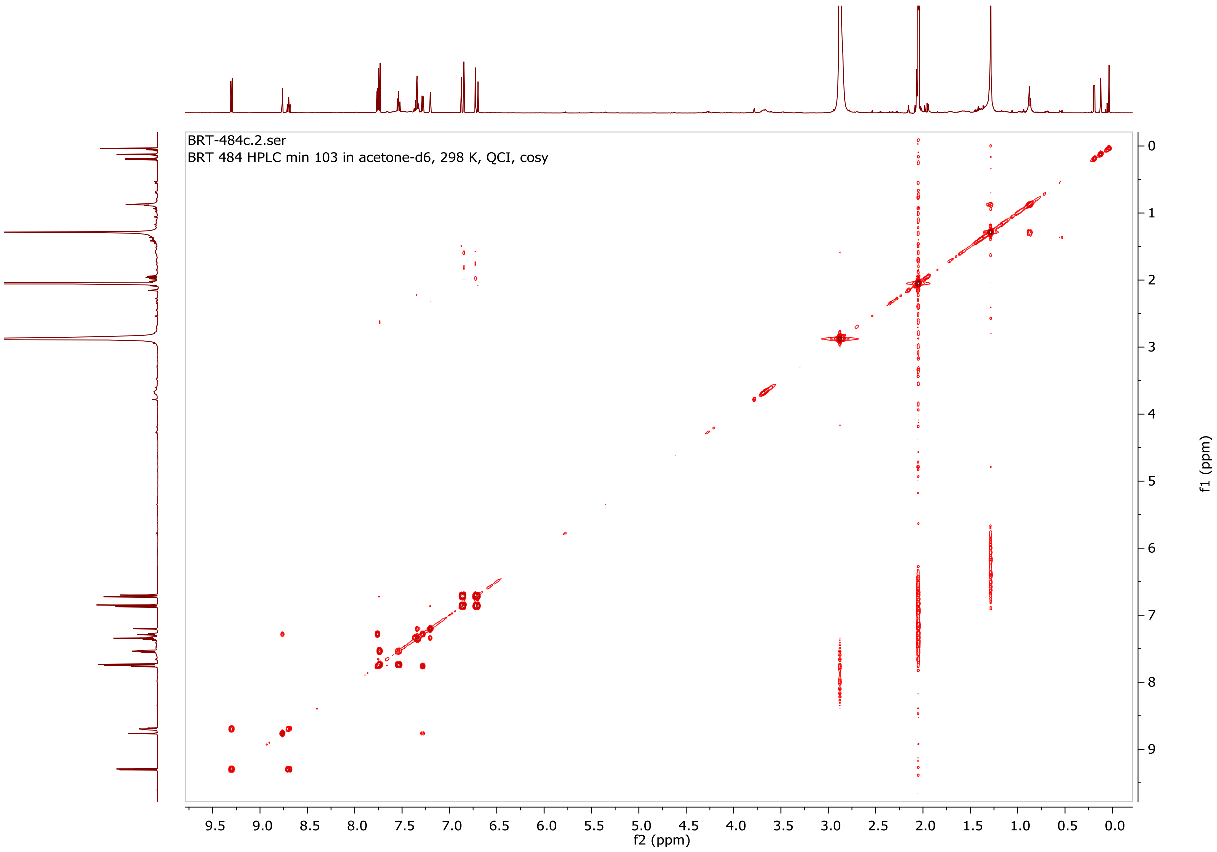

ROESY-NMR spectrum

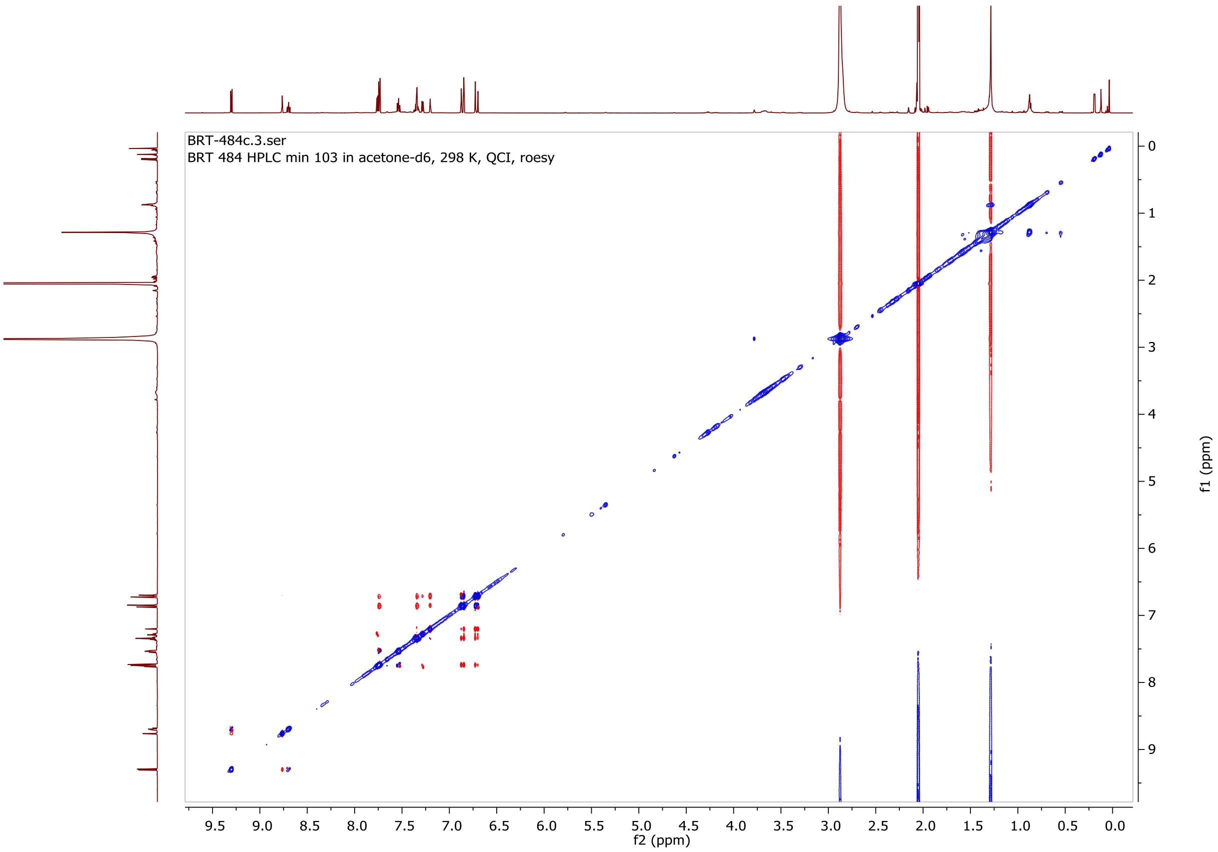

HSQC-NMR spectrum

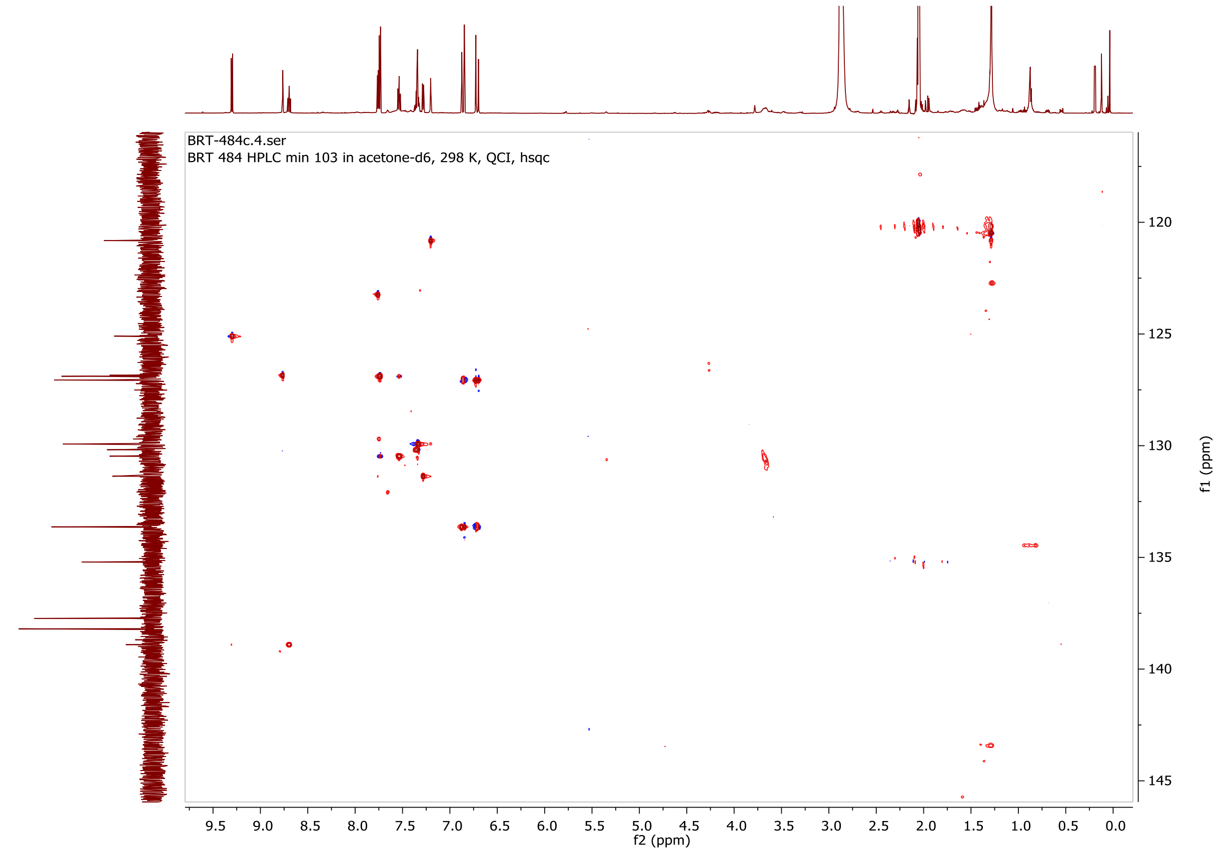

HMBC-NMR spectrum

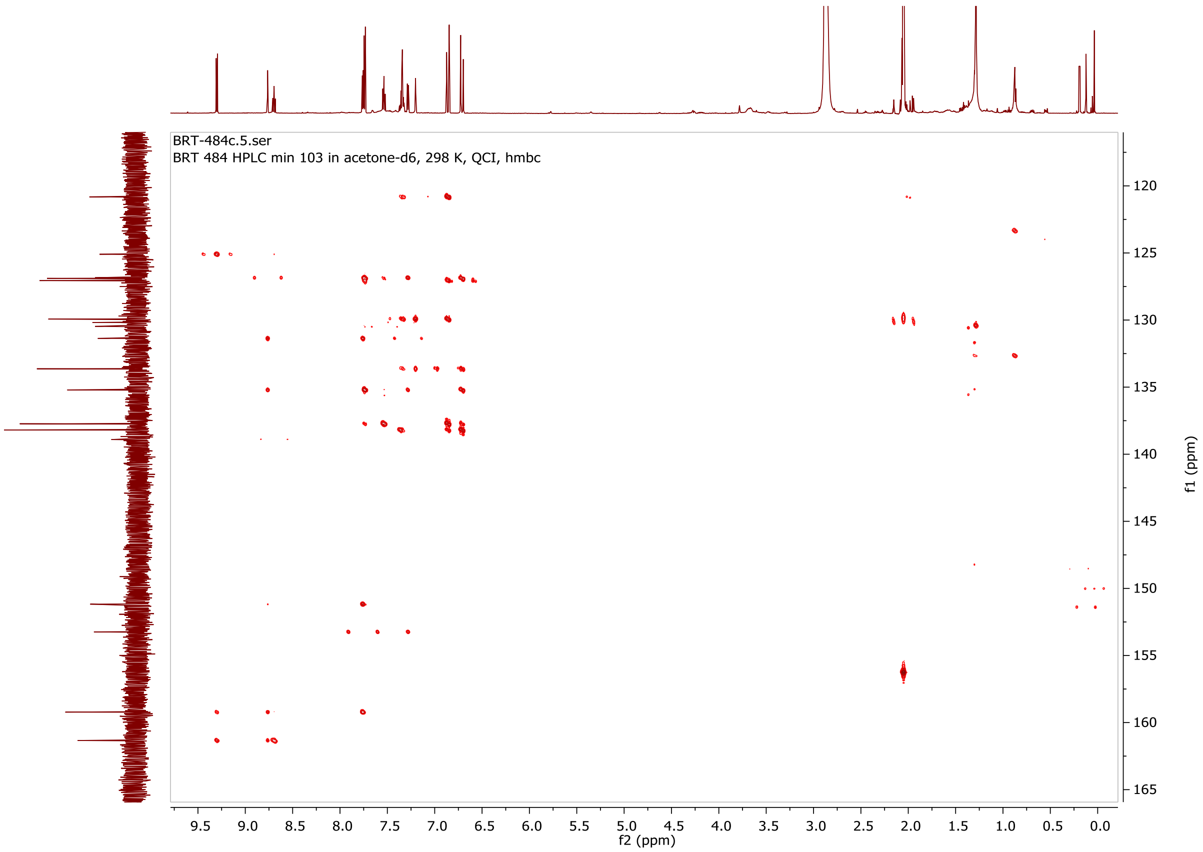

Full assignment of the all-trans isomer of 1

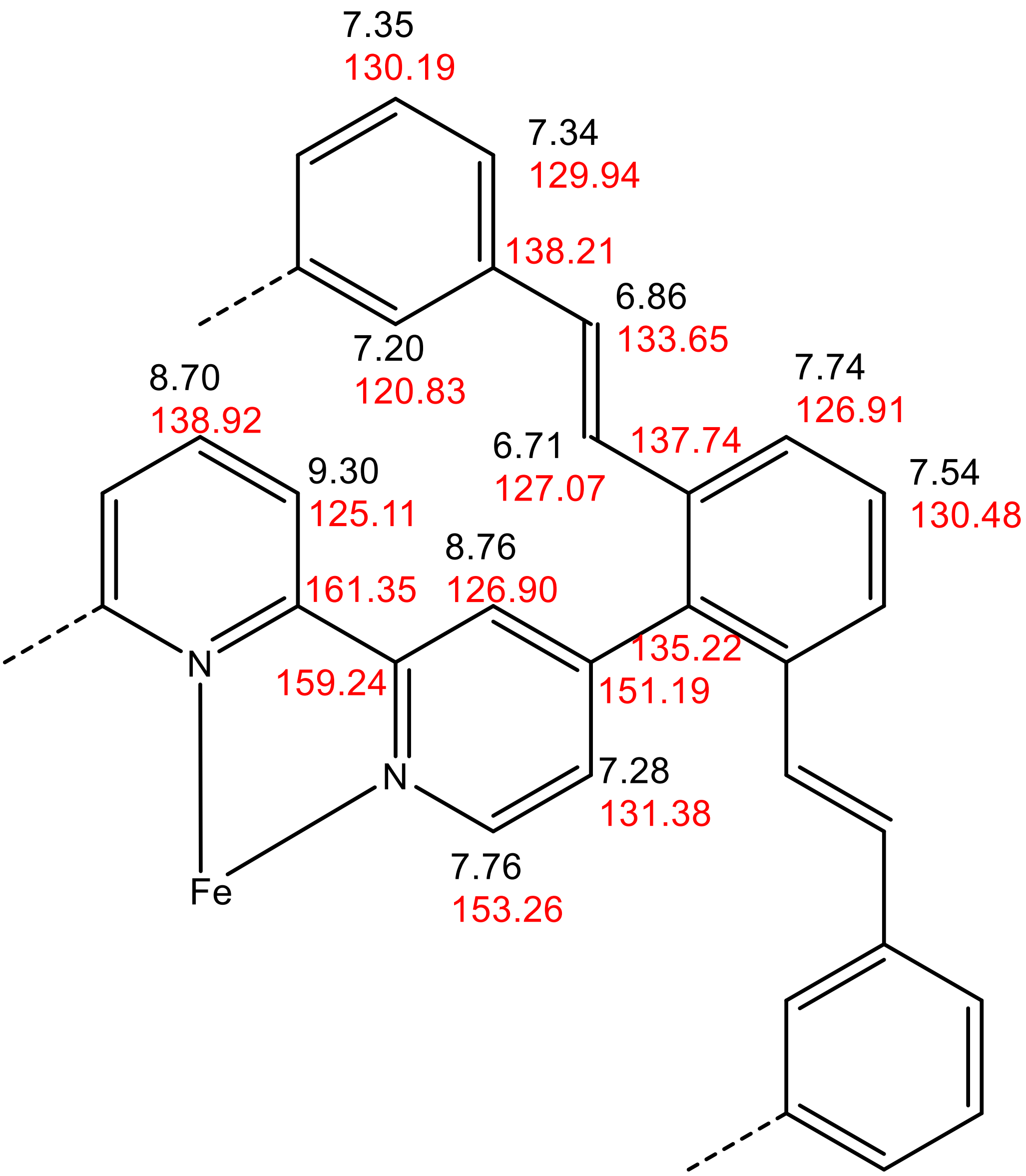

Figure SI2: Full-assignment of compound **1** in acetone-d6, 298 K, 600 MHz,QCI.

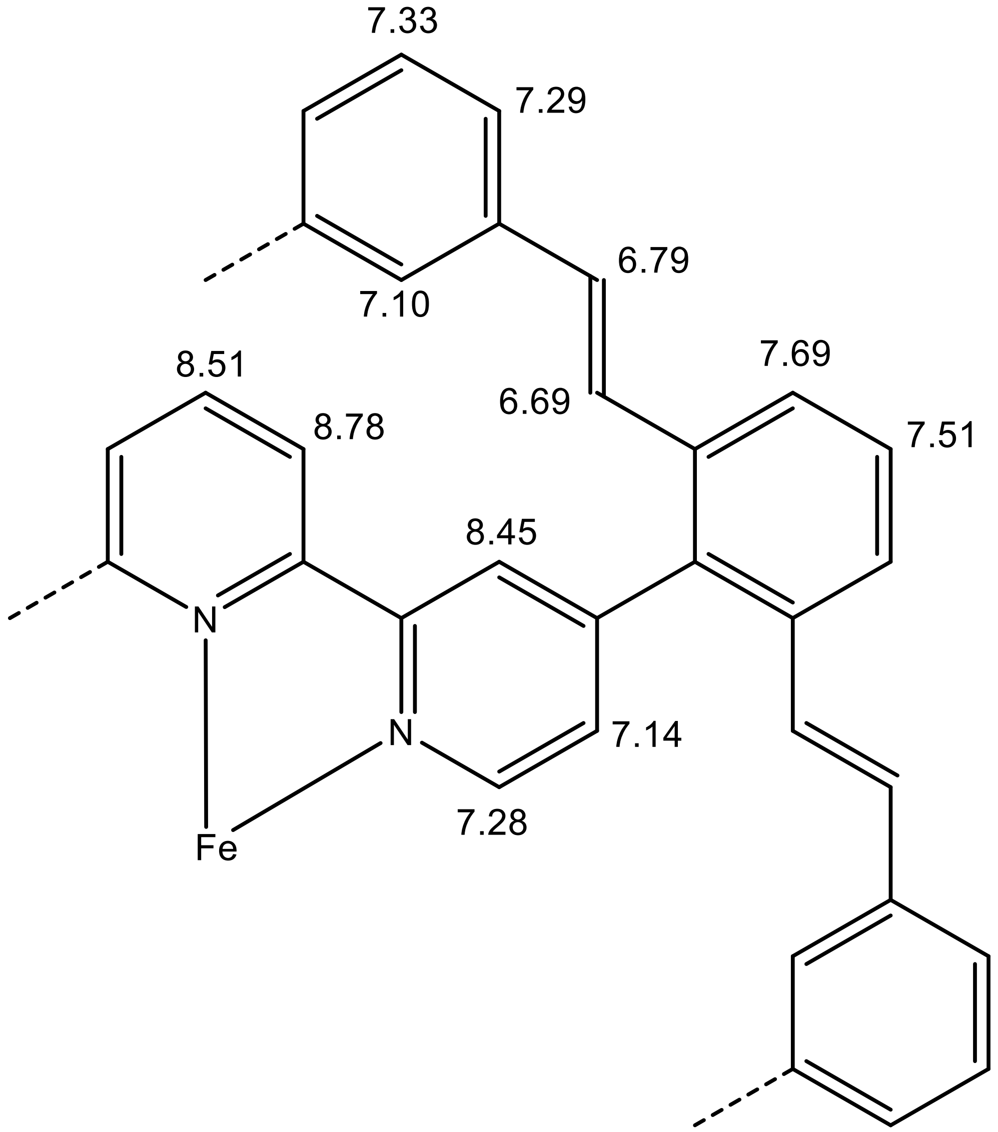

Figure SI3: Proton-assignment of compound **1** in acetonitrile-d_3, 298 K, 600 MHz,BBFO.

Mass Spectrum SmartFormula Report

Analysis Info

Analysis Name	E:\ESI Results Backup\AK Mayor\Thomas Brandl\BRT484_HPLC_min103.d	Acquisition Date	26.11.2018 10:30:22
Method	22 Direct_pos_mid.m	Operator	Miff
Sample Name	Thomas Brandl / BRT484_HPLC_min103	Instrument / Ser#	maXis 4G 21243
Comment	10 ug/mL in acetone, analyzed in acetonitrile		

Acquisition Parameter

Source Type	ESI	Ion Polarity	Positive	Set Nebulizer	0.4 Bar
Focus	Not active	Set Capillary	3600 V	Set Dry Heater	180 °C
Scan Begin	75 m/z	Set End Plate Offset	-500 V	Set Dry Gas	4.0 l/min
Scan End	1700 m/z	Collision Energy	8.0 eV	Set Ion Energy (MS only)	4.0 eV

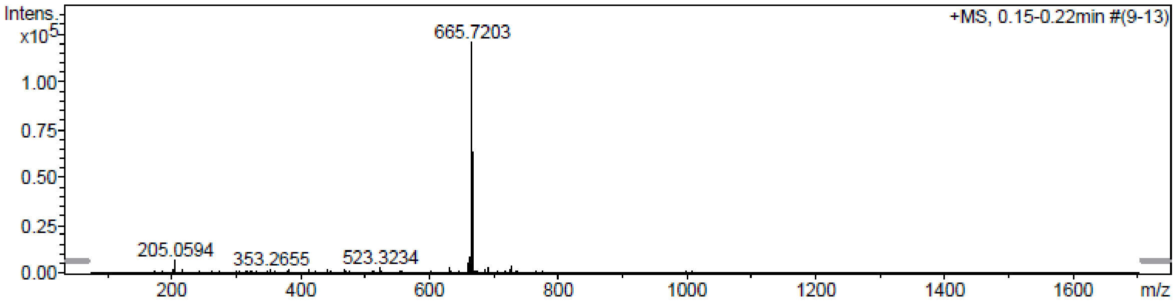

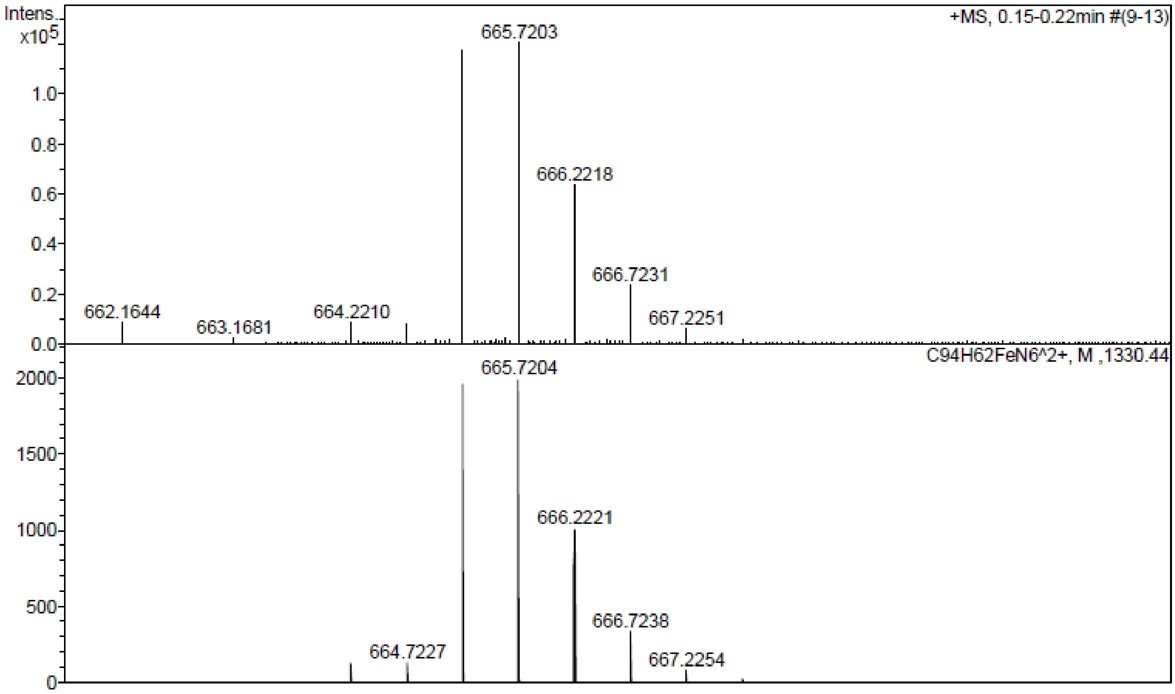

Meas. m/z	#	Formula	Score	m/z	err [mDa]	err [ppm]	mSigma	rdb	e⁻ Conf	z
665.2189	1	C 94 H 62 Fe N 6	100.00	665.2188	-0.1	-0.2	8.2	67.0	even	2+

UV-Vis Spectra of Compound 8 and Compound 1

The UV-Vis spectrum of compound **8** was recorded on a Shimadzu UV spectrometer UV-1800. The wavelength was measured in nm. The solution was measured under air saturated conditions in acetonitrile. The spectrum was recorded using optical 1115F-QS Hellma cuvettes (10 mm light path).

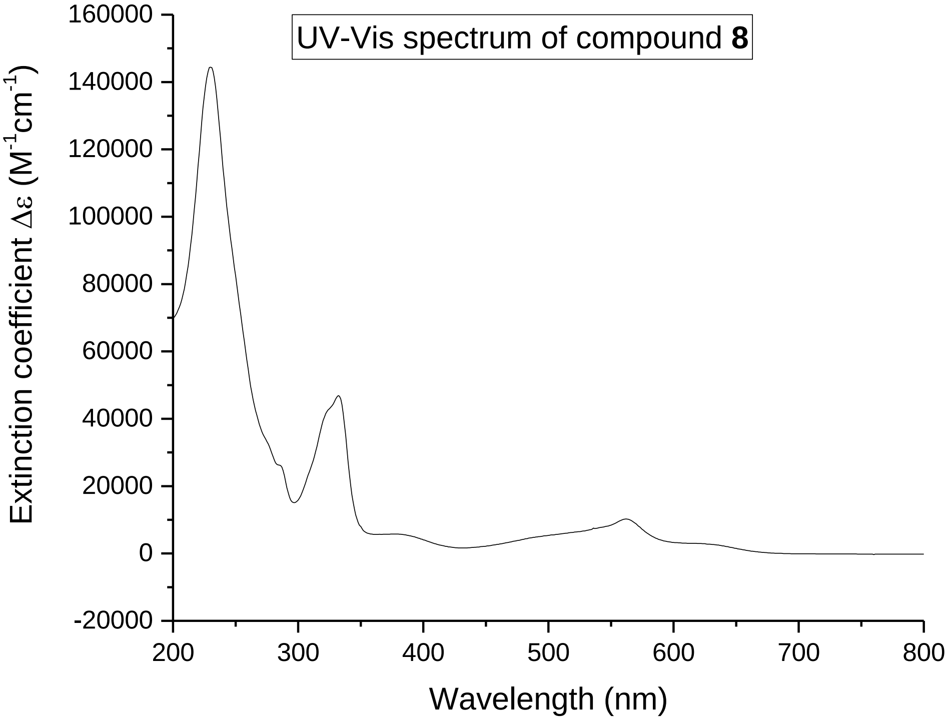

Figure SI4: UV-Vis spectrum of compound **8**.

Table SI1: UV-Vis data for compound **8**

Wavelength λ [nm]	Extinction coefficient Δε [M^{-1}cm^{-1}]	λ [nm]	Δε [M^{-1} cm^{-1}]	λ [nm]	Δε [M^{-1} cm^{-1}]	λ [nm]	Δε [M^{-1} cm^{-1}]
800	-161.5508	755	-161.5508	710	-88.5659	665	576.4987
799	-161.5508	754	-161.5508	709	-88.5659	664	615.0412
798	-161.5508	753	-161.5508	708	-88.5659	663	661.7844
797	-161.5508	752	-161.5508	707	-88.5659	662	688.0261
796	-161.5508	751	-161.5508	706	-88.5659	661	783.9726
795	-161.5508	750	-161.5508	705	-88.5659	660	822.5152
794	-161.5508	749	-161.5508	704	-88.5659	659	895.5001
793	-161.5508	748	-161.5508	703	-88.5659	658	917.6416
792	-161.5508	747	-161.5508	702	-88.5659	657	1029.9891
791	-161.5508	746	-151.7101	701	-88.5659	656	1068.5317
790	-161.5508	745	-151.7101	700	-88.5659	655	1153.8174
789	-161.5508	744	-151.7101	699	-88.5659	654	1241.5633
788	-161.5508	743	-151.7101	698	-76.2651	653	1314.5482
787	-161.5508	742	-151.7101	697	-76.2651	652	1361.2913
786	-161.5508	741	-151.7101	696	-76.2651	651	1409.6746
785	-161.5508	740	-151.7101	695	-59.8640	650	1522.0221
784	-161.5508	739	-151.7101	694	-59.8640	649	1570.4054
783	-161.5508	738	-139.4093	693	-50.0233	648	1645.8504
782	-161.5508	737	-151.7101	692	-50.0233	647	1768.0386
781	-161.5508	736	-151.7101	691	-50.0233	646	1806.5812
780	-161.5508	735	-151.7101	690	-50.0233	645	1863.1650
779	-161.5508	734	-151.7101	689	-37.7225	644	1963.2117
778	-161.5508	733	-151.7101	688	-27.8818	643	2036.1966
777	-161.5508	732	-151.7101	687	21.3214	642	2074.7392
776	-161.5508	731	-139.4093	686	62.3241	641	2137.8835
775	-161.5508	730	-139.4093	685	62.32418	640	2260.0717
774	-161.5508	729	-139.4093	684	74.62501	639	2282.2131
773	-161.5508	728	-123.0082	683	84.46567	638	2345.3574
772	-161.5508	727	-123.0082	682	84.46567	637	2433.1033
771	-161.5508	726	-123.0082	681	84.46567	636	2489.6871
770	-161.5508	725	-123.0082	680	110.7074	635	2528.2297
769	-161.5508	724	-123.0082	679	123.0082	634	2554.4714
768	-161.5508	723	-123.0082	678	123.0082	633	2591.3739
767	-161.5508	722	-123.0082	677	157.4505	632	2662.7187
766	-161.5508	721	-123.0082	676	186.1525	631	2688.9605
765	-161.5508	720	-123.0082	675	195.9931	630	2735.7036
764	-161.5508	719	-123.0082	674	257.4972	629	2752.1047
763	-161.5508	718	-123.0082	673	291.9396	628	2774.2462
762	-161.5508	717	-123.0082	672	330.4821	627	2784.0868
761	-161.5508	716	-123.0082	671	330.4821	626	2837.3904
760	-351.8036	715	-123.0082	670	369.0247	625	2896.4344
759	-161.5508	714	-123.0082	669	403.4670	624	2908.7352
758	-161.5508	713	-98.4066	668	442.0096	623	2934.9770
757	-161.5508	712	-98.4066	667	503.5138	622	2959.5786
756	-161.5508	711	-98.4066	666	537.9561	621	2971.8794

λ [nm]	Δε [M^{-1} cm^{-1}]	λ [nm]	Δε [M^{-1} cm^{-1}]	λ [nm]	Δε [M^{-1} cm^{-1}]	λ [nm]	Δε [M^{-1} cm^{-1}]
620	2981.7201	574	7489.5627	528	6666.2274	482	4380.7340
619	2981.7201	573	7847.9267	527	6617.8442	481	4307.7491
618	2998.1212	572	8125.9254	526	6554.6999	480	4195.4016
617	3010.4220	571	8496.5903	525	6495.6560	479	4147.0183
616	3020.2627	570	8815.5917	524	6466.9540	478	4061.7326
615	3020.2627	569	9071.4489	523	6420.2109	477	3949.3851
614	3020.2627	568	9346.1673	522	6381.6683	476	3901.0018
613	3020.2627	567	9575.7827	521	6371.8277	475	3854.2587
612	3020.2627	566	9821.7992	520	6259.4801	474	3744.3713
611	3020.2627	565	10007.1317	519	6237.3386	473	3681.2271
610	3067.0058	564	10167.8625	518	6211.0969	472	3608.2422
609	3083.4069	563	10202.3048	517	6164.3537	471	3520.4963
608	3083.4069	562	10253.1482	516	6135.6518	470	3435.2105
607	3083.4069	561	10202.3048	515	6039.7054	469	3396.668
606	3142.4509	560	10151.4614	514	6013.4636	468	3290.8809
605	3154.7517	559	10007.1317	513	5991.3221	467	3227.7366
604	3171.1528	558	9821.7992	512	5928.1779	466	3189.1940
603	3205.5951	557	9697.9709	511	5889.6353	465	3067.0058
602	3217.8960	556	9490.4970	510	5830.5913	464	3028.4632
601	3227.7366	555	9307.6247	509	5767.4471	463	2943.1775
600	3266.2792	554	9100.1508	508	5745.3056	462	2847.2311
599	3300.7215	553	8900.8774	507	5694.4622	461	2808.6885
598	3388.4674	552	8742.6068	506	5672.3207	460	2735.7036
597	3451.6116	551	8569.5752	505	5584.5748	459	2697.1610
596	3473.7531	550	8425.2455	504	5537.8317	458	2601.2146
595	3536.8974	549	8323.5587	503	5511.5899	457	2562.6720
594	3624.6433	548	8189.0696	502	5499.2891	456	2499.5277
593	3697.6282	547	8116.0847	501	5426.3042	455	2479.8464
592	3758.3122	546	8055.4007	500	5348.3990	454	2367.4989
591	3880.5004	545	7943.0531	499	5313.9567	453	2328.9563
590	3965.7862	544	7879.9089	498	5275.4141	452	2282.2131
589	4110.1158	543	7831.5256	497	5253.2726	451	2243.6706
588	4211.8027	542	7735.5792	496	5190.1283	450	2172.3258
587	4372.5335	541	7697.0366	495	5131.0844	449	2121.4824
586	4504.5623	540	7624.0517	494	5067.9401	448	2109.1815
585	4677.5939	539	7526.4652	493	5029.3976	447	2070.6389
584	4901.4690	538	7451.0201	492	5007.2561	446	2036.1966
583	5084.3412	537	7419.0380	491	4894.9085	445	1948.4507
582	5291.8152	536	7564.1877	490	4885.0679	444	1926.3093
581	5527.9910	535	7254.2069	489	4821.9236	443	1901.7076
580	5774.0075	534	7119.7179	488	4783.3810	442	1863.1650
579	6029.8647	533	7046.7330	487	4677.5939	441	1853.3244
578	6275.8812	532	6949.1464	486	4639.0513	440	1816.4219
577	6544.0392	531	6873.7013	485	4575.9071	439	1790.1801
576	6902.4033	530	6800.7165	484	4537.3645	438	1728.6760
575	7170.5613	529	6712.9706	483	4419.2766	437	1718.8353

λ [nm]	Δε [M^{-1} cm^{-1}]	λ [nm]	Δε [M^{-1} cm^{-1}]	λ [nm]	Δε [M^{-1} cm^{-1}]	λ [nm]	Δε [M^{-1} cm^{-1}]
436	1677.8326	390	5201.6091	344	15050.4702	298	15188.2395
435	1655.6911	389	5274.5940	343	17382.7068	297	15102.9537
434	1655.6911	388	5386.9416	342	20168.4338	296	15125.0952
433	1655.6911	387	5435.3248	341	23292.0234	295	15399.8137
432	1655.6911	386	5498.4690	340	27056.8961	294	15926.2890
431	1655.6911	385	5561.6133	339	30994.8005	293	16808.6682
430	1655.6911	384	5632.9581	338	34947.4658	292	18051.0516
429	1655.6911	383	5671.5007	337	38470.4223	291	19536.9914
428	1655.6911	382	5718.2438	336	41653.0559	290	21252.5465
427	1677.8326	381	5744.4856	335	44157.5040	289	23062.4080
426	1718.8353	380	5756.7864	334	45853.3778	288	24634.4535
425	1740.9768	379	5756.7864	333	46717.7159	287	25630.8204
424	1816.4219	378	5766.6270	332	46878.4467	286	26106.4523
423	1853.3244	377	5766.6270	331	46520.0826	285	26232.7408
422	1891.8669	376	5766.6270	330	45836.9767	284	26291.7848
421	1948.4507	375	5756.7864	329	45078.4258	283	26450.0554
420	1974.6925	374	5756.7864	328	44366.6180	282	26925.6873
419	2087.0400	373	5744.4856	327	43811.4408	281	27735.0817
418	2121.4824	372	5718.2438	326	43408.793	280	28680.6051
417	2194.4670	371	5744.4856	325	43025.8280	279	29760.6176
416	2333.0566	370	5718.2438	324	42635.4818	278	30791.4268
415	2367.4989	369	5718.2438	323	42161.4900	277	31727.1096
414	2440.4838	368	5718.2438	322	41510.3663	276	32562.7457
413	2538.0703	367	5681.3413	321	40564.0228	275	33284.3942
412	2623.3560	366	5693.6421	320	39535.6738	274	33974.0605
411	2725.0429	365	5718.2438	319	38235.8865	273	34577.6210
410	2847.2311	364	5693.6421	318	36726.9852	272	35358.3134
409	2932.5168	363	5681.3413	317	35079.4947	271	36181.6486
408	3093.2476	362	5693.6421	316	33412.3227	270	37204.2573
407	3178.5333	361	5693.6421	315	31814.0355	269	38369.5555
406	3313.0223	360	5670.6806	314	30278.0724	268	39687.3840
405	3424.5498	359	5766.6270	313	28829.8551	267	41012.5929
404	3559.0389	358	5766.6270	312	27565.3303	266	42464.0903
403	3670.5663	357	5851.9128	311	26397.5719	265	44015.6345
402	3814.8960	356	5974.1010	310	25342.1610	264	45662.3050
401	3965.7862	355	6097.9293	309	24312.992	263	47577.1336
400	4060.9125	354	6280.8016	308	23282.1828	262	49740.4388
399	4221.6433	353	6526.8181	307	22202.1703	261	52190.7632
398	4306.9291	352	6845.8195	306	21057.3734	260	54846.1015
397	4504.5623	351	7377.2151	305	20016.7236	259	57498.9796
396	4565.2464	350	8030.7990	304	18936.7111	258	60168.2587
395	4703.8357	349	8368.6617	303	17974.7865	257	62922.8236
394	4798.9621	348	9157.5547	302	17118.6490	256	65710.1907
393	4933.4511	347	10225.2663	301	16419.1421	255	68438.5139
392	5018.7368	346	11488.1511	300	15849.2038	254	71207.8398
391	5091.7217	345	13145.4823	299	15508.0609	253	74021.4486

λ [nm]	Δε [M^{-1} cm^{-1}]	λ [nm]	Δε [M^{-1} cm^{-1}]
252	76849.8185	206	74144.4569
251	79592.0825	205	73170.2315
250	82371.2491	204	72238.6489
249	85048.7288	203	71398.0925
248	87886.1193	202	70788.7916
247	90692.3476	201	70149.1487
246	93537.1186	200	69882.6308
245	96599.2041		
244	99709.6729		
243	103026.7956		
242	106655.5392		
241	110549.9806		
240	114384.5580		
239	118617.6821		
238	123041.8791		
237	127341.4277		
236	131222.7482		
235	135017.9630		
234	138330.1653		
233	141261.0420		
232	143129.1274		
231	144265.7237		
230	144419.0740		
229	144317.3872		
228	143157.8294		
227	141225.7797		
226	138807.4373		
225	135928.2241		
224	132375.7456		
223	128352.5556		
222	123829.1319		
221	119347.5311		
220	115005.3396		
219	110463.8748		
218	106188.1078		
217	102153.4370		
216	98239.3142		
215	94575.3083		
214	91224.5634		
213	88094.4133		
212	85305.4060		
211	82891.1640		
210	80599.1101		
209	78577.6745		
208	76839.1578		
207	75365.5188		

The UV-Vis spectrum of compound **1** was recorded on a Jasco V-770 spectrophotometer. The wavelength was measured in nm. The solution was measured under air saturated conditions in acetonitrile. The spectrum was recorded using optical 1115F-QS Hellma cuvettes (10 mm light path).

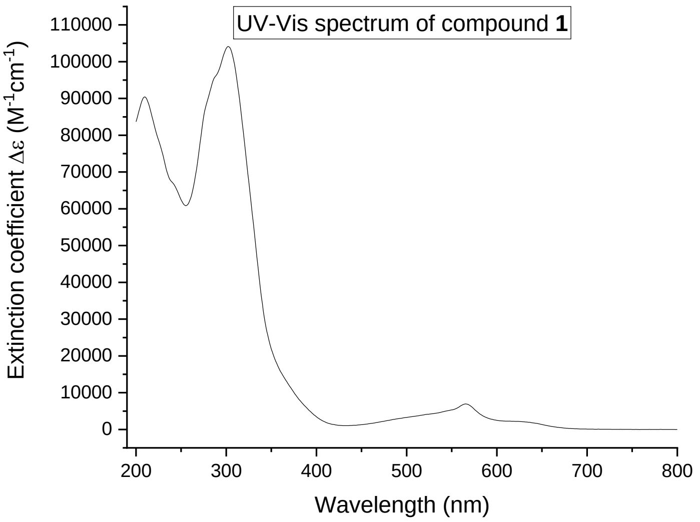

Figure SI5: UV-Vis spectrum of compound **1**.

Table SI2: UV-Vis data for compound **1**

Wavelength λ [nm]	Extinction coefficient Δε [M^{-1}cm^{-1}]	λ [nm]	Δε [M^{-1} cm^{-1}]	λ [nm]	Δε [M^{-1} cm^{-1}]	λ [nm]	Δε [M^{-1} cm^{-1}]
800	-26.4574	755	3.412625	710	65.92816	665	663.3109
799	-25.0814	754	18.1451	709	74.35112	664	698.5857
798	-26.3043	753	-3.64195	708	73.36165	663	731.3999
797	-16.0478	752	6.278621	707	90.14079	662	769.6306
796	2.91433	751	7.110329	706	96.84158	661	811.7446
795	-11.2044	750	0.893387	705	93.84598	660	847.0939
794	-4.60728	749	15.65123	704	97.42146	659	894.0154
793	-6.41298	748	9.385055	703	94.43528	658	933.5789
792	-20.4771	747	9.621734	702	95.08576	657	977.3002
791	-11.0752	746	13.59087	701	107.4241	656	1030.227
790	-16.267	745	10.66038	700	109.3625	655	1081.746
789	-18.1543	744	11.3772	699	110.6543	654	1140.706
788	-21.7893	743	14.32197	698	117.0383	653	1185.526
787	-17.1119	742	20.83243	697	114.8166	652	1239.916
786	-12.9394	741	12.41767	696	116.3414	651	1291.109
785	-13.8813	740	35.17847	695	117.9285	650	1340.369
784	-4.555	739	28.71058	694	118.0769	649	1395.276
783	-3.28811	738	32.66352	693	124.1879	648	1447.904
782	8.556238	737	45.54457	692	139.6457	647	1503.228
781	14.0331	736	40.99197	691	152.7792	646	1553.383
780	5.509954	735	33.37415	690	173.1029	645	1604.456
779	0.464773	734	35.3598	689	184.4344	644	1645.083
778	-15.848	733	26.89324	688	189.9146	643	1682.659
777	-10.4971	732	34.57986	687	201.9543	642	1722.513
776	-4.86724	731	26.34628	686	210.6084	641	1755.277
775	-10.7333	730	21.66426	685	221.788	640	1793.024
774	-9.17705	729	20.91697	684	235.4526	639	1833.11
773	-21.4213	728	19.02435	683	239.941	638	1867.233
772	-20.8634	727	38.97127	682	246.9171	637	1910.808
771	-9.80198	726	49.31061	681	266.5474	636	1946.527
770	0.533917	725	56.40832	680	275.4496	635	1972.805
769	9.782258	724	53.0347	679	289.5676	634	2004.86
768	6.223819	723	55.97787	678	317.2244	633	2025.145
767	3.785546	722	51.18831	677	326.687	632	2045.823
766	2.088633	721	43.83246	676	351.2945	631	2072.656
765	3.415407	720	48.24306	675	373.688	630	2088.542
764	8.37243	719	47.81309	674	395.2923	629	2106.885
763	5.471269	718	56.81656	673	427.4348	628	2124.204
762	4.48875	717	61.22031	672	456.0116	627	2142.354
761	-6.08725	716	69.28873	671	484.4003	626	2156.213
760	-13.7537	715	69.60957	670	503.7416	625	2173.039
759	-15.7313	714	74.20042	669	523.7362	624	2188.857
758	-28.3318	713	77.58419	668	552.3626	623	2192.75
757	-10.2826	712	83.11194	667	585.3304	622	2199.844
756	-2.72054	711	76.3868	666	624.0671	621	2206.67

λ [nm]	Δε [M^{-1} cm^{-1}]	λ [nm]	Δε [M^{-1} cm^{-1}]	λ [nm]	Δε [M^{-1} cm^{-1}]	λ [nm]	Δε [M^{-1} cm^{-1}]
620	2204.511	574	5725.733	528	4261.977	482	2567.492
619	2216.055	573	5955.05	527	4237.247	481	2520.12
618	2230.371	572	6168.453	526	4207.35	480	2473.739
617	2236.502	571	6367.201	525	4181.937	479	2432.742
616	2246.509	570	6533.913	524	4146.008	478	2386.034
615	2251.691	569	6686.476	523	4118.485	477	2333.903
614	2258.563	568	6807.055	522	4102.452	476	2289.509
613	2262.365	567	6887.283	521	4057.504	475	2245.294
612	2260.322	566	6932.654	520	4024.226	474	2193.964
611	2268.801	565	6936.215	519	3980.251	473	2156.464
610	2264.689	564	6891.753	518	3944.34	472	2114.451
609	2274.617	563	6815.79	517	3910.468	471	2061.611
608	2287.129	562	6707.547	516	3868.659	470	2026.381
607	2299.693	561	6572.331	515	3827.876	469	1987.936
606	2325.499	560	6430.652	514	3777.256	468	1939.962
605	2341.156	559	6275.212	513	3730.661	467	1901.225
604	2362.353	558	6117.265	512	3694.577	466	1849.136
603	2382.165	557	5963.482	511	3656.642	465	1806.944
602	2410.94	556	5828.358	510	3622.565	464	1771.02
601	2444.581	555	5708.39	509	3592.027	463	1735.579
600	2484.123	554	5598.881	508	3562.524	462	1700.85
599	2527.981	553	5506.302	507	3525.531	461	1658.375
598	2571.475	552	5428.627	506	3485.669	460	1623.261
597	2617.106	551	5368.256	505	3442.188	459	1581.226
596	2666.864	550	5319.007	504	3406.362	458	1544.877
595	2726.826	549	5274.897	503	3371.04	457	1507.857
594	2783.68	548	5221.537	502	3336.63	456	1467.239
593	2852.064	547	5169.132	501	3302.211	455	1443.316
592	2923.026	546	5120.417	500	3267.169	454	1407.95
591	3000.644	545	5072.521	499	3244.745	453	1378.358
590	3090.959	544	5028.151	498	3211.033	452	1345.868
589	3188.235	543	4979.494	497	3173.243	451	1312.009
588	3291.395	542	4917.895	496	3137.075	450	1306.558
587	3396.001	541	4862.022	495	3095.416	449	1286.104
586	3511.743	540	4801.881	494	3051.813	448	1263.933
585	3641.147	539	4736.543	493	3017.48	447	1240.089
584	3773.702	538	4680.227	492	2982.929	446	1216.599
583	3923.118	537	4621.27	491	2950.128	445	1185.416
582	4081.868	536	4562.041	490	2921.615	444	1157.416
581	4247.229	535	4514.334	489	2881.206	443	1147.008
580	4434.999	534	4475.34	488	2833.455	442	1137.525
579	4629.638	533	4431.12	487	2788.044	441	1131.29
578	4839.874	532	4396.832	486	2744.6	440	1124.049
577	5048.326	531	4358.183	485	2700.614	439	1094.286
576	5266.655	530	4315.093	484	2659.653	438	1070.749
575	5493.93	529	4287.233	483	2608.969	437	1063.099

λ [nm]	Δε [M^{-1} cm^{-1}]	λ [nm]	Δε [M^{-1} cm^{-1}]	λ [nm]	Δε [M^{-1} cm^{-1}]	λ [nm]	Δε [M^{-1} cm^{-1}]
436	1043.882	390	5670.963	344	27933.69	298	102494.4
435	1042.238	389	5924.302	343	29256.96	297	101750.4
434	1047.454	388	6170.114	342	30672.8	296	100943.4
433	1037.262	387	6439.647	341	32280.1	295	100113.2
432	1041.997	386	6684.605	340	34246.22	294	99268.48
431	1043.728	385	6949.174	339	36088.56	293	98483.49
430	1045.602	384	7228.812	338	38047.75	292	97760.54
429	1059.36	383	7485.084	337	40159.29	291	97164.15
428	1073.902	382	7797.029	336	42335.67	290	96693.1
427	1084.184	381	8084.745	335	44586.88	289	96325.24
426	1103.343	380	8390.287	334	46907.66	288	96017.76
425	1121.037	379	8714.716	333	49253.95	287	95668.08
424	1138.346	378	9013.734	332	51564.71	286	95193.02
423	1174.942	377	9355.275	331	53863.55	285	94555.58
422	1212.688	376	9708.354	330	56167.79	284	93745.3
421	1257.456	375	10066.69	329	58407.32	283	92829.24
420	1305.66	374	10433.73	328	60675.64	282	91866.52
419	1340.129	373	10784.57	327	62896.49	281	90948
418	1375.421	372	11153.06	326	65099.26	280	90093.41
417	1425.922	371	11519.65	325	67321.52	279	89299.05
416	1481.517	370	11883.09	324	69542.44	278	88490.77
415	1555.087	369	12255.23	323	71784.31	277	87557.14
414	1634.363	368	12634.3	322	74006.02	276	86439.32
413	1702.068	367	13039.63	321	76240.05	275	85077.91
412	1778.332	366	13413.32	320	78455.11	274	83480.44
411	1873.145	365	13796.97	319	80687.26	273	81692.41
410	2009.071	364	14199.91	318	82904.22	272	79802.52
409	2110.079	363	14580.54	317	85064.88	271	77844.87
408	2224.98	362	14985.18	316	87163.84	270	75936.18
407	2348.888	361	15407.95	315	89188.91	269	74088.88
406	2478.893	360	15838.1	314	91144.38	268	72315.88
405	2617.681	359	16321.1	313	93007.3	267	70660.98
404	2756.609	358	16836.26	312	94793.13	266	69112.01
403	2900.313	357	17358.39	311	96493.85	265	67682.44
402	3077.233	356	17925.3	310	98039.63	264	66381.88
401	3257.398	355	18492.57	309	99442.45	263	65218.47
400	3443.893	354	19087.87	308	100710.3	262	64172.8
399	3647.975	353	19728.38	307	101765.9	261	63261.6
398	3842.007	352	20394.37	306	102648.7	260	62499.02
397	4047.437	351	21107.09	305	103344.8	259	61882.52
396	4267.025	350	21874.12	304	103819.8	258	61400.66
395	4482.205	349	22698.93	303	104076.3	257	61083.91
394	4702.813	348	23606.53	302	104123.6	256	60899.6
393	4939.265	347	24567.55	301	103964.3	255	60855.98
392	5167.821	346	25619.76	300	103627.3	254	60972.89
391	5414.556	345	26723.55	299	103113	253	61195.38

λ [nm]	Δε [M^{-1} cm^{-1}]	λ [nm]	Δε [M^{-1} cm^{-1}]
252	61525.43	206	88946.29
251	61965.02	205	88139.63
250	62444.33	204	87251.4
249	62984.67	203	86296.12
248	63563.58	202	85350.02
247	64130.15	201	84471.12
246	64706.5	200	83665.39
245	65275.58		
244	65792.69		
243	66249.11		
242	66647.23		
241	66954.11		
240	67219.8		
239	67480		
238	67797.55		
237	68221.89		
236	68794.43		
235	69516.63		
234	70353.96		
233	71310.3		
232	72337.43		
231	73386.11		
230	74416.98		
229	75394.29		
228	76285.41		
227	77114.98		
226	77886.82		
225	78641.92		
224	79399.54		
223	80183.02		
222	81030.56		
221	81917.7		
220	82872.94		
219	83852.28		
218	84843.87		
217	85824.17		
216	86756.93		
215	87663.75		
214	88484.58		
213	89203.66		
212	89801.52		
211	90210.93		
210	90401.2		
209	90375.73		
208	90108.16		
207	89614.11		

The combined UV-Vis spectra of compound **1** and **8** can be found in figure SI6. Furthermore, the absorption maxima for both compounds are summarized in table SI3. It can be seen that the metal-to-ligand charge transfer (MLCT) absorption is in the same range for both complexes. Only a small bathochromic shift for the cage compound **1** of 3 nm is observed. The intensity of the MLCT band is slightly decreased upon macrocyclization. These minuscule spectral changes indicate that the surrounding ring system does not distort the coordination geometry at the metal center and is only weakly coupled to the complex' tpy ligand system. A bigger difference can be observed for the ligand-centered (LC) transitions. For the aldehyde complex **8** a weak transition at 332 nm is observed, whereas we observe a much more intense transition for the complex **1** at 302 nm. This intense transition is attributed to the macrocycle formed upon interlinking the ligands in **1** resulting in a large delocalized π-system.

Table SI3: Absorption maxima for compound **1** and compound **8**

Compound **1**		Compound **8**	
Wavelength λ_{max} [nm]	Extinction coefficient $\Delta\varepsilon$ [$M^{-1}cm^{-1}$]	Wavelength λ_{max} [nm]	Extinction coefficient $\Delta\varepsilon$ [$M^{-1}cm^{-1}$]
210	90401	233	144419
302	104124	332	46878
565	6936	562	10253

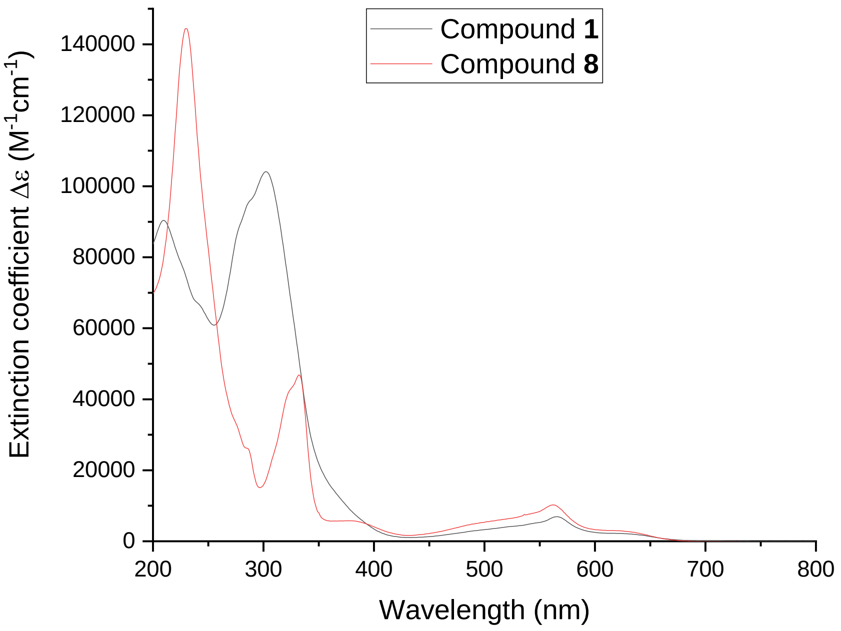

Figure SI6: Combined UV-Vis spectrum of compound **1** and compound **8**.

Fluorescence spectra of the complexes **1** and **8** were recorded in degassed acetonitrile at room temperature using quartz fluorescence cuvettes with a 1 cm optical path length on a Horiba Jobin–Yvon FluoroMax 4 fluorimeter. However, no emission of the complexes was observed under these conditions.

Crystal Data of 8 and Unsuccessful Crystallization Attempts of 1

Single crystals of compound **8** were grown by vapor diffusion technique with acetonitrile as solvent and diethyl ether as anti-solvent. Solid state structure of complex **8** with rotation ellipsoids at 50% probability are displayed. Hydrogen atoms, solvent molecules and the PF_6^- counter ions were omitted for clarity. Color code: N: blue, Fe: orange, O: red and C: gray.

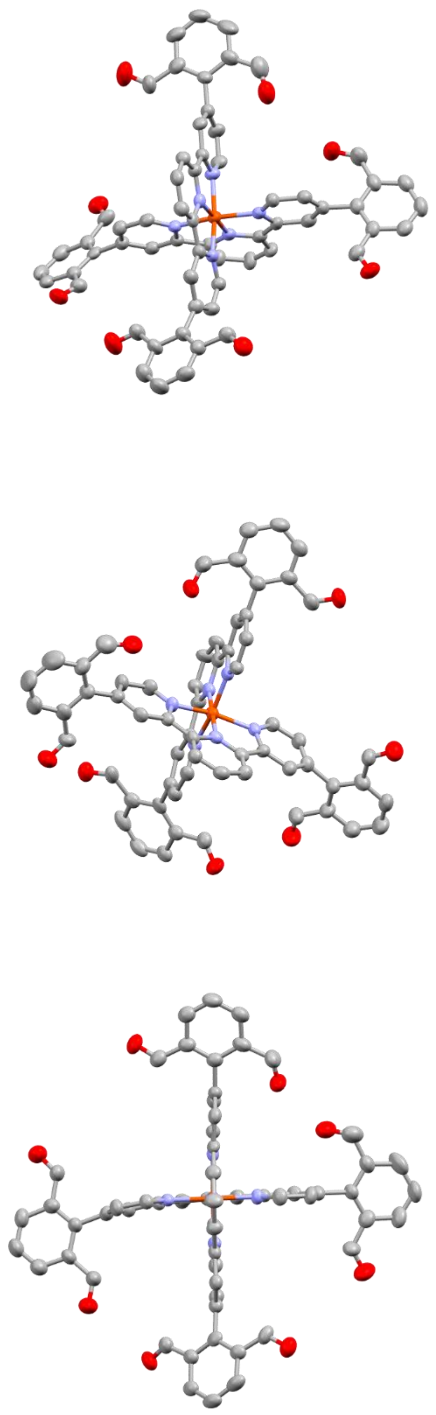

Figure SI7: Solid state structure of the **1** with rotation ellipsoids at 50% probability. Three different illustrations are presented. Hydrogen atoms, solvent molecules and the PF_6^- counter ions are omitted for clarity. Color code: Fe: orange, N: blue, O: red, C: gray.

Crystal data for compound **8**: formula $C_{70}H_{50}F_{12}Fe N_{10}O_8P_2$, M = 1504.99, F(000) = 3072, purple plate, size 0.03 x 0.16 x 0.18 mm^3, space group *P 21/c* , Z = 4, *a* = 16.5679(10) Å, *b* = 33.819(2) Å, *c* = 12.1822(6) Å, *α* = 90°, *β* = 105.135(4)°, *γ* = 90°, V = 6589.0(7) Å^3, $D_{calc.}$ = 1.517 Mg * m^{-3}. The crystal was measured on a Bruker APEX-II CCD diffractometer at 130K using graphite-monochromated CuK_α-radiation with λ = 1.54178 Å, Θ_{max} = 70.216°. Minimal/maximal transmission 0.6021/0.7533, μ = 3.170 mm^{-1}. The Apex2 suite has been used for data collection and integration. From a total of 46856 reflections, 11902 were independent (merging r = 0.1077). From these, 11902 were considered as observed (I > 2σ(I)) and were used to refine 900 parameters. The structure was solved by dual methods using the program SHELXT 2014/5. Least-squares refinement against Fsqd was carried out on all non-hydrogen atoms using the program ShelXL. R = 0.1246 (observed data), wR = 0.3881 (all data), GOF = 1.091. Minimal/maximal residual electron density = -1.123/1.513 e Å^{-3}. Chebychev polynomial weights were used to complete the refinement. Plots were produced using Olex2. Crystallographic data (excluding structure factors) for the structure in this paper have been deposited with the Cambridge Crystallographic Data Center, the deposition number is (1957472). Copies of the data can be obtained, free of charge, on application to the CCDC, 12 Union Road, Cambridge CB2 1EZ, UK [fax: +44-1223-336033 or e-mail: deposit@ccdc.cam.ac.uk].

Crystallization Attempts of 1: In spite of numerous attempts, crystals suitable for x-ray diffraction analysis of complex **1** could not be obtained. Based on our experience with Fe(II)tpy$_2$ model complexes, crystallization attempts of **1** were based on both, vapor diffusion and the solvent layering techniques. In particular the following combinations were investigated repeatedly without success. Solutions of **1** in acetonitrile, acetone, methanol, and dichloromethane were exposed to the “anti-solvents” diethyl ether, diisopropyl ether, heptane, hexane, petroleum ether, water, toluene, tetrahydrofuran, cyclohexane, and isopropanol. Also to vary the initial concentration of **1** in the solvents did not result in crystals of the required quality.

SQUID Measurements

The magnetic measurements were performed on a SQUID magnetometer (Quantum design, model MPMS-XL-5). For standard measurements, the temperature dependent magnetization was recorded at B=0.1 Tesla as external magnetic field. The temperature was sweeped in the range of 5 – 365 K at the rate 3 K min^{-1}. Gelatine capsules were used as sample holders. The diamagnetic corrections of the molar magnetic susceptibilities were applied using Pascal's constants. From these measurements, we can infer that these compounds (**9**, **8** and **1**) showed structurally low-spin state as the high-spin molecules show XT value around 3.5 emu mol^{-1} K.[5] Therefore, we concluded that all the compounds were diamagnetic and no SCO behavior was observed upon heating.

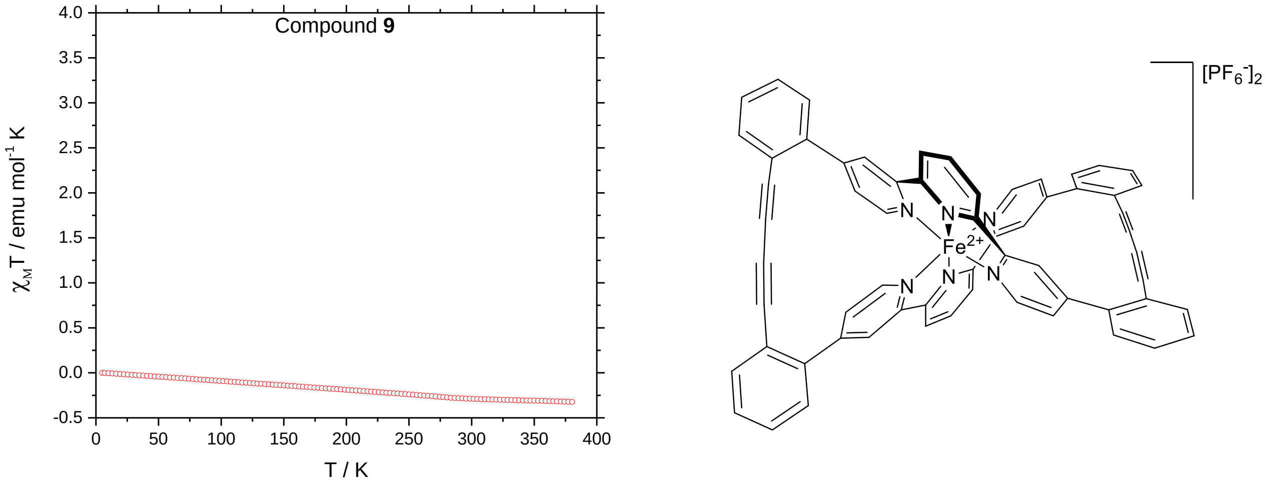

Figure SI8: SQUID measurement of compound **9** (left) and the corresponding structure (right).

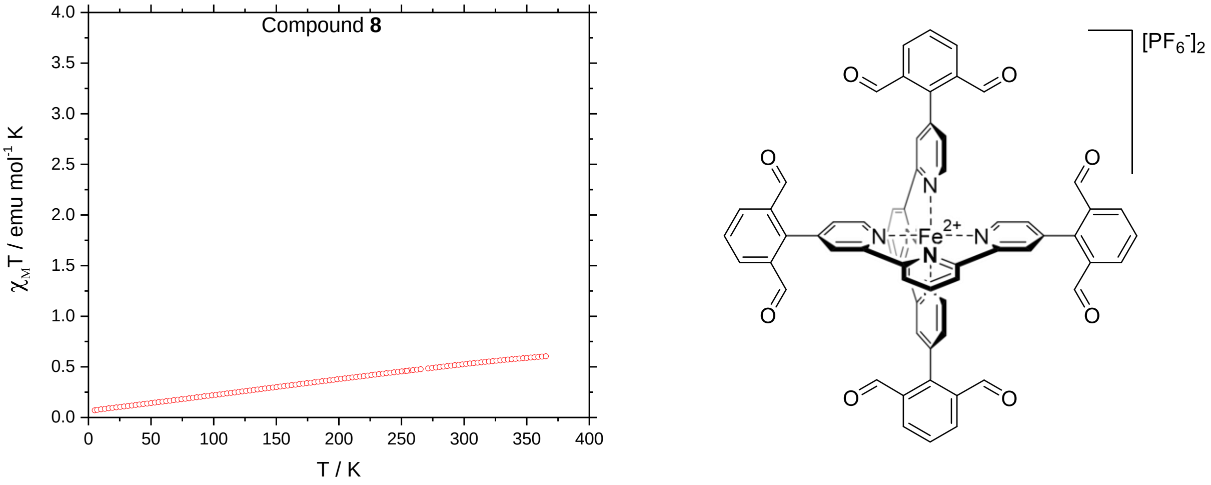

Figure SI9: SQUID measurement of compound **8** (left) and the corresponding structure (right).

[5] Brooker, *Chem. Soc. Rev.*, **2015**, 44, 2880-2892.

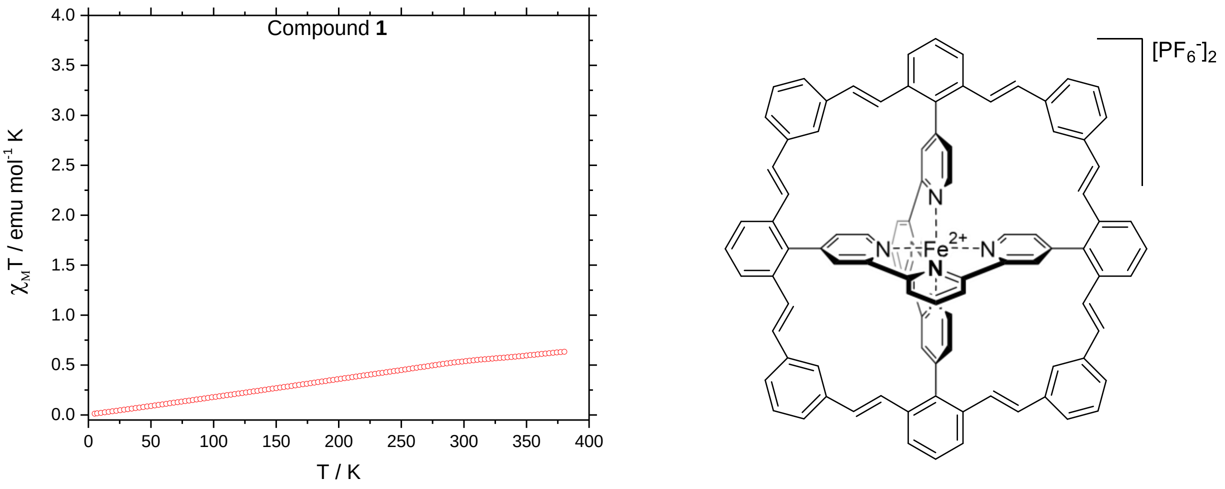

Figure SI10: SQUID measurement of compound **1** (left) and the corresponding structure (right).

IR-Spectra

Infrared spectra were measured with a Bruker Platinum ATR Alpha in the region of 900-200 cm^{-1}. The spectra were recorded at room temperature with 150 scans and with a resolution of 2.0 cm^{-1}. The idea was to identify the Fe-N vibration to confirm the increasing stiffness upon interlinking of the structures. Unfortunately, the vibration could not be identified.

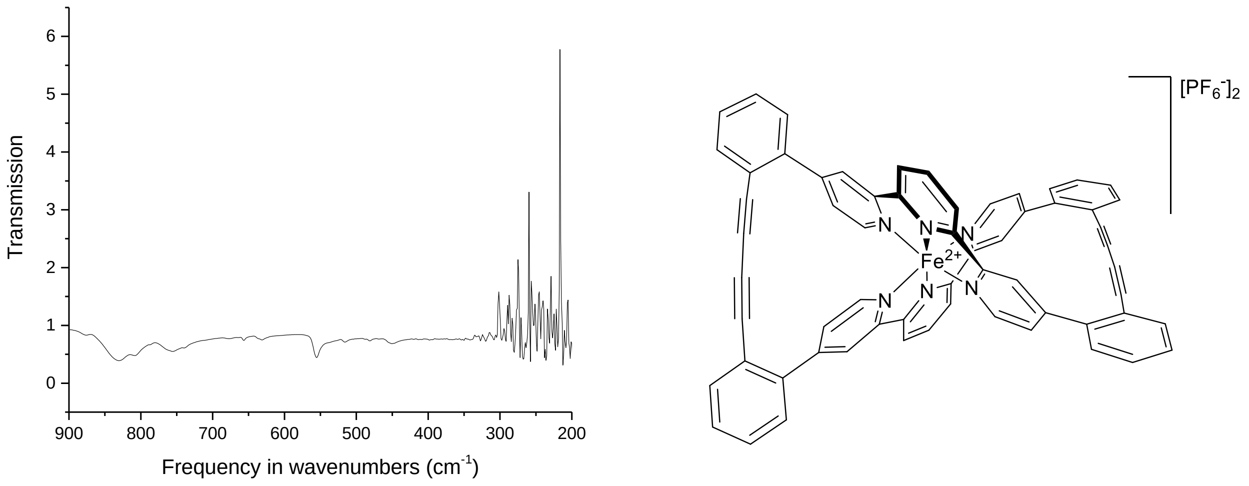

Figure SI11: IR-spectrum of compound **9** (left) and corresponding structure (right).

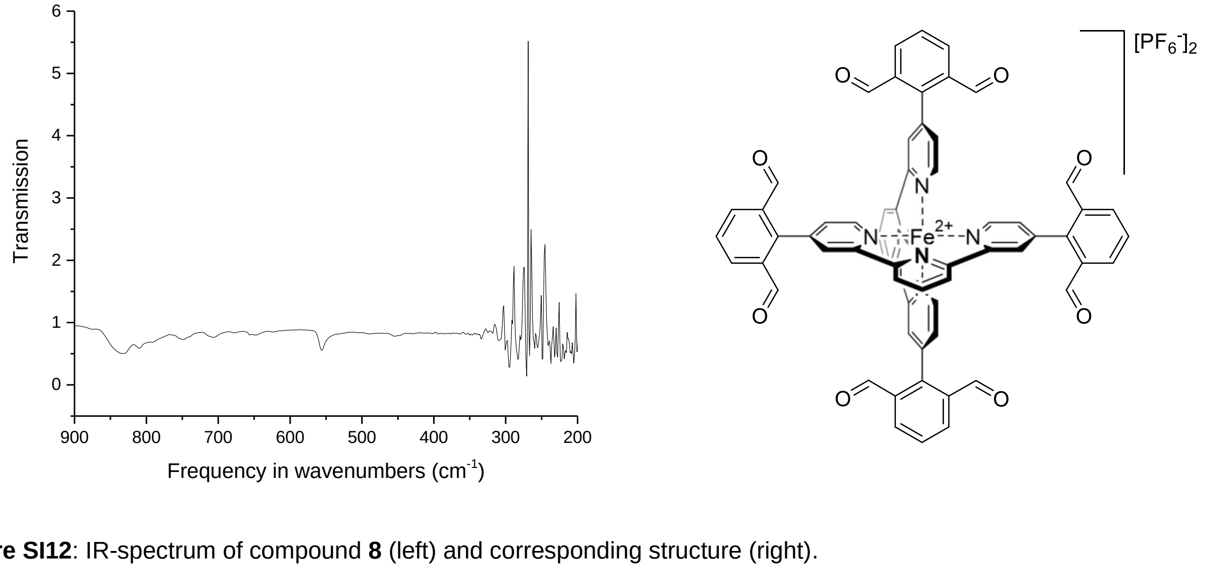

Figure SI12: IR-spectrum of compound **8** (left) and corresponding structure (right).

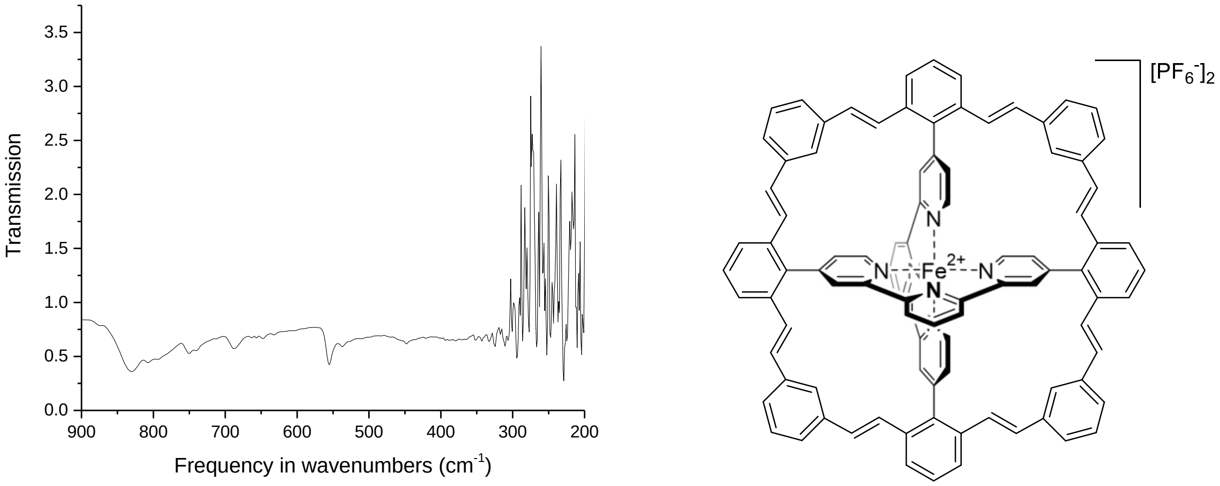

Figure SI13: IR-spectrum of compound **1** (left) and corresponding structure (right).

Determination of the Spin-Spin Relaxation Times T_2 of Compound 8 and all-*trans* Isomer 1 by NMR Spectroscopy

The structures of **1** and **8** (illustrated in figure SI14) suggest an increased rigidity of the macrocyclized complex **1** compared to that of **8** as the isophthalaldehyde groups can freely rotate, which induces motion and flexibility all over the molecule. In order to gain insight into the dynamic behaviour of all-*trans* **1** and **8** we determined the spin-spin relaxation time T_2 for all protons in **1** and **8** using a *Carr-Purcell-Meiboom-Gill* (CPMG) spin echo pulse sequence. An increase in T_2 time at constant molecular weight is a very sensitive probe for the intramolecular mobility of a spin. The protons in the following figures and tables are assigned according to figure SI14.

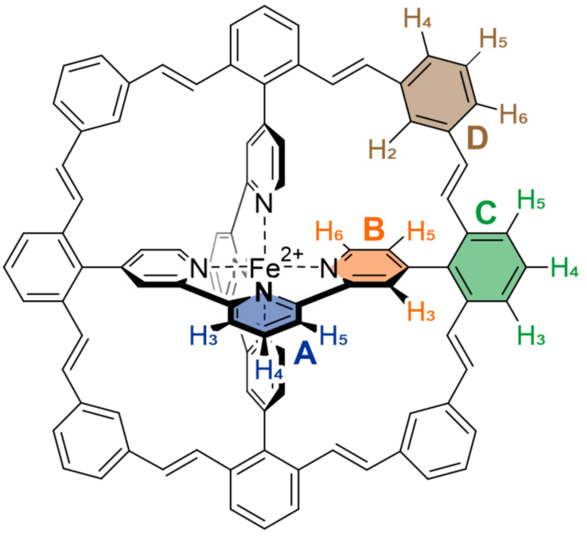

1

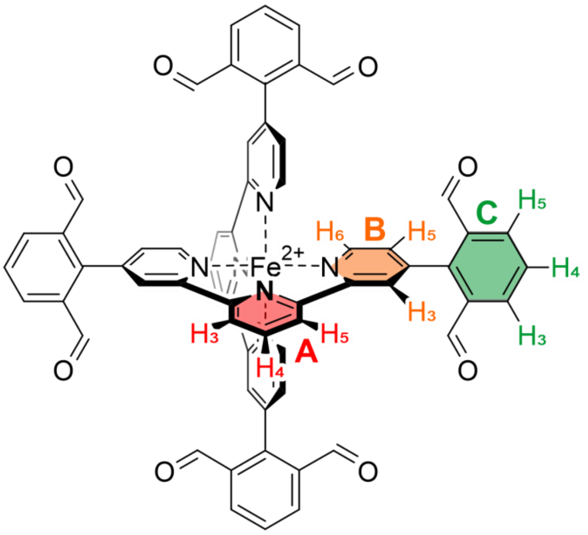

8

Figure SI14: Proton assignment for the complexes **1** and **8**.

The comparison for complex **1** and for complex **8** of the measured T_2 relaxation times is depicted in table SI4**.** A representative plot of the relaxation times for the proton A: H_3/H_5 of both complexes is illustrated in figure SI15.

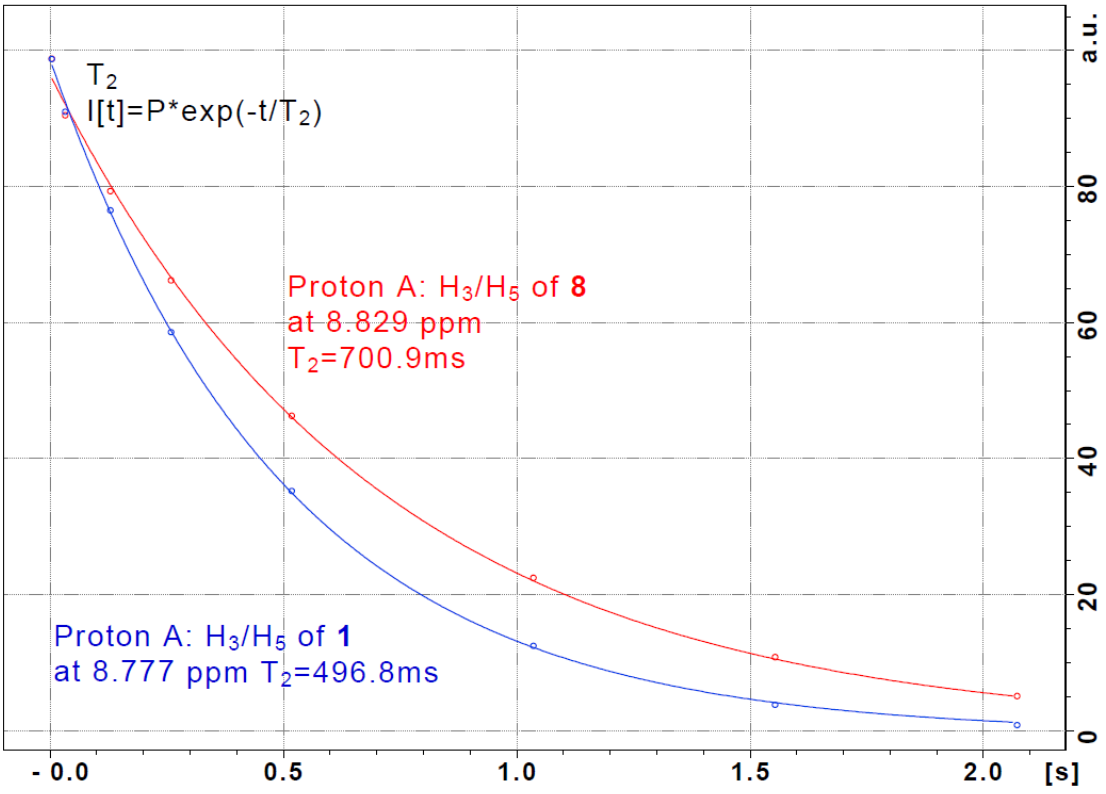

Figure SI15: Representative plot of the spin-spin relaxation time T_2 of Protons A: H_3/H_5 for compound **8** (red) and **1** (blue). The spectrum illustrates the faster relaxation of the more rigid complex **1**.

Table SI4: Proton T_2 relaxation times for compound **8** and compound **1**

Proton	T_2 [s] in **8**	T_2 [s] in **1**
A: H_3/H_5	0.700(10)	0.496(10)
A: H_4	0.884(10)	0.506(10)
B: H_3	0.877(10)	0.729(10)
B: H_5	1.138(10)	0.721(10)
B: H_6	1.128(10)	0.752(10)
C: H_3/H_5	1.319(10)	0.658(10)
C: H_4	1.026(10)	0.629(10)
D: H_2	n/a	0.509(10)
D: H_4/H_6	n/a	0.691(10)
D: H_5	n/a	0.687(10)
CHO	2.126(10)	n/a
C: Olefin H	n/a	0.614(10)
D: Olefin H	n/a	0.645(10)

To our delight, the analysis revealed highly significant differences between the central terpyridine units in **1** (0.496 to 0.752 s) and **8** (0.700 to 1.128 s, Table SI4) where for every single proton the T_2 time of **1** is decreased by a factor of 1.20 to 1.75 with respect to **8**, indicating a drastic rigidification. The data even allow to localize the most mobile regions of the molecules: While the protons on the central pyridine ring **A** are the most static part, the *ortho*-positioned **B** rings are considerably more mobile in both compounds. While the mobility of the

strained linkage of the **C** and **D** rings is intermediate in **1**, the **C** ring and the aldehyde protons in **8** show an up to three-fold T_2 increase, as can be expected due to the higher degree of rotational freedom. Overall the T_2 times of **1** are much more uniform throughout the molecule (max/min: 1.52), whereas the spread is 3.04 in **8**.

The molecular weight increase of 20.9% between **1** and **8** is too small to explain the observed differences in T_2 times, but aggregation of **1** to oligomeric assemblies would lead to effects in the observed order of magnitude. Therefore, also the hydrodynamic radii of both compounds in acetonitrile by diffusion ordered NMR spectroscopy were determined and a difference in diffusion coefficients of 5.2% corresponding to a mass increase for solvated **1** of 16.4% with respect to **8**, thus corroborating the proposed rigidification by the four-fold interlinkage was found.

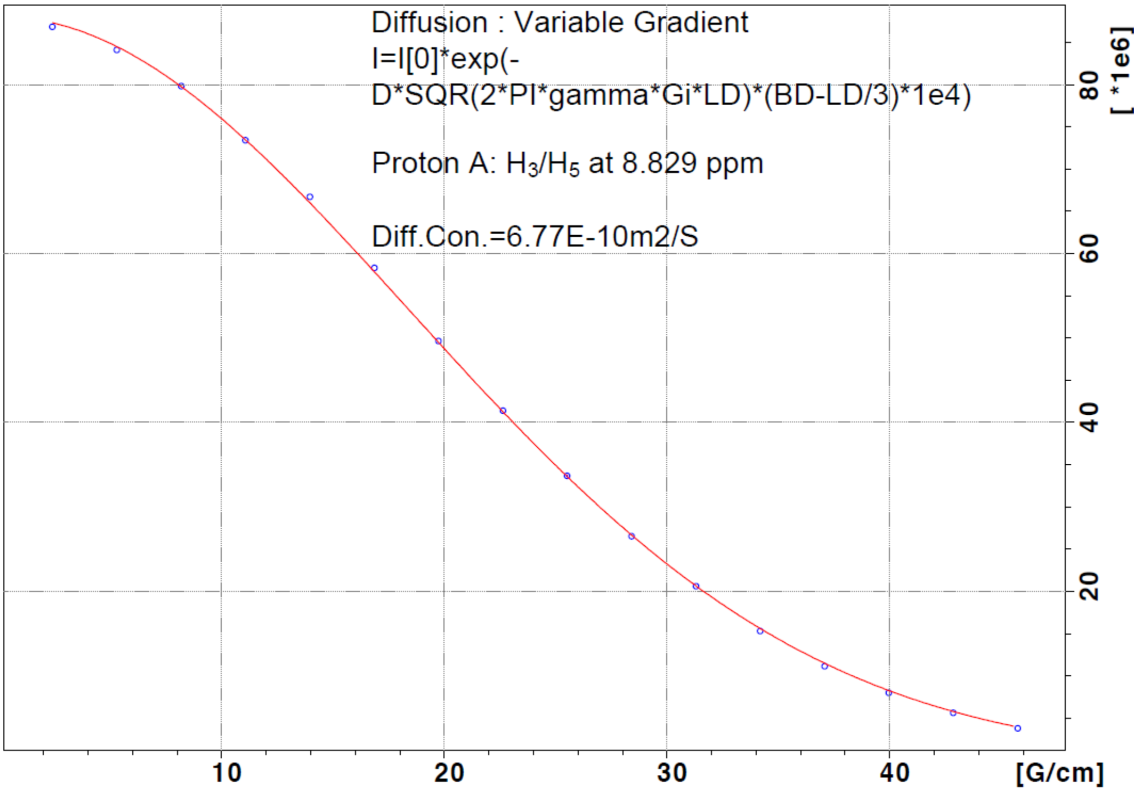

Figure SI16: Diffusion ordered NMR spectroscopy of compound **8**: Experimental intensity (blue circles) and 2-parameter fit (red line).

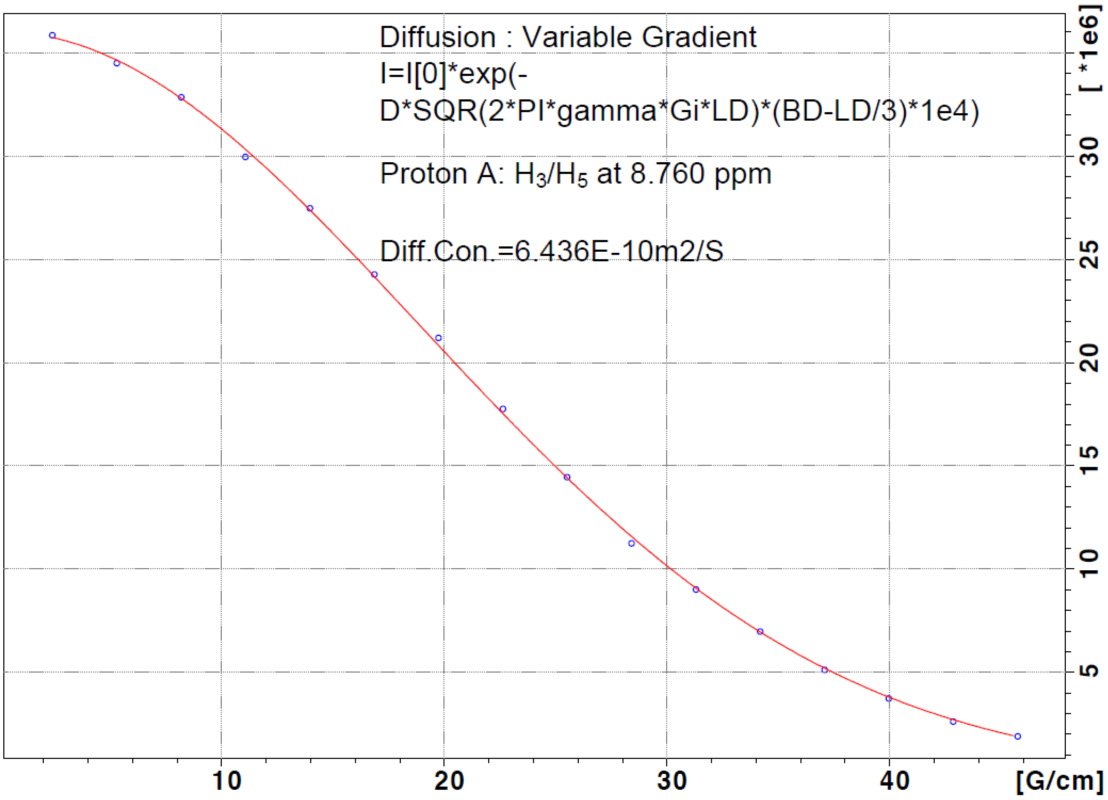

Figure SI17: Diffusion ordered NMR spectroscopy of compound **1**: Experimental intensity (blue circles) and 2-parameter fit (red line).

Monitoring the Imine Condensation towards the Fourfold Interlinked Complex 2 by DI-ESI-MS and NMR Spectroscopy

The course of the eightfold imine condensation forming the fourfold interlinked complex **2** was monitored by direct injection electrospray mass spectrometry (DI-ESI-MS). Representative spectra recorded of the crude reaction mixture are displayed in figure SI18 and figure SI19. In figure SI18 the course of the reaction after several minutes is depicted. The masses of the starting material and different intermediates, namely singly interlinked, twofold interlinked, threefold interlinked and the fourfold interlinked target structure **2** can be identified. Moreover, even an unwanted eightfold imine condensation product comprising five diamine subunits (three closed bridges and two additional bridging units masking both remaining aldehydes) can be observed.

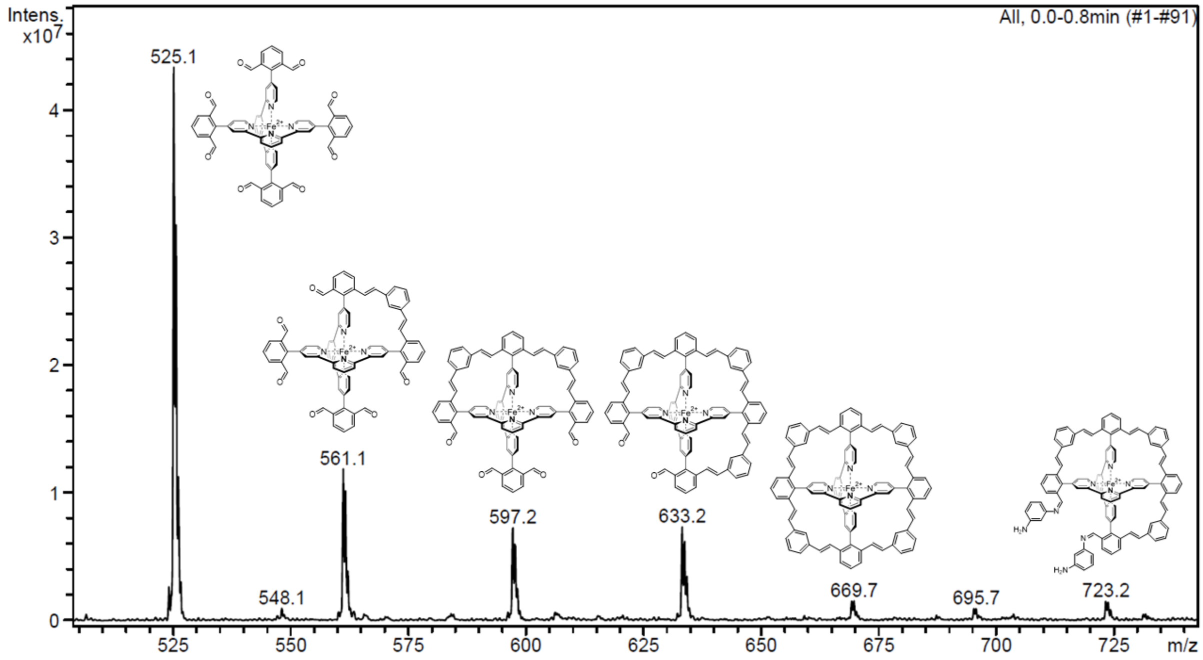

Figure SI18: DI-ESI-MS displaying the composition of the condensation reaction. The structures matching the corresponding mass peaks are displayed.

As described in the main text, performing the condensation reaction with an eightfold excess of *meta*-phenylenediamine in refluxing 1,2-dichloroethane (83.5 °C) for 10 days resulted in the DI-ESI-mass spectrum displayed in Figure SI19, with the mass of 670 m/z corresponding to the desired fourfold interlinked octaimine complex **2** as major peak. However, some of the signals like e.g. the threefold interlinked intermediate (633 m/z) or the overreacted side-product (723 m/z) are less pronounced, but still present.

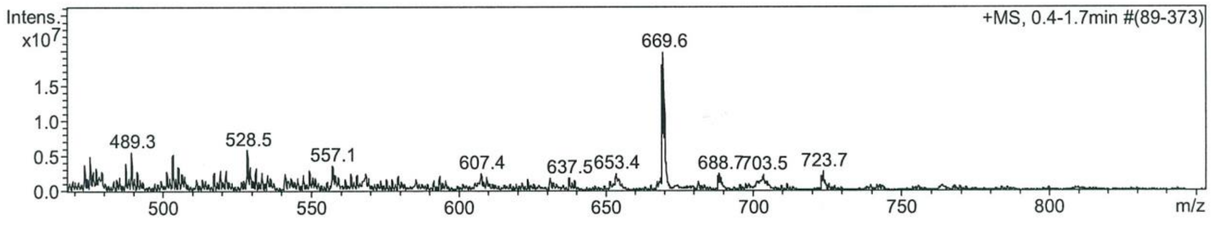

Figure SI19: DI-ESI-MS spectrum of the imine condensation applying the improved reaction conditions favoring the formation of **2** (8 equivalents of the diamine in refluxing 1,2-dichloroethane (83.5 °C) for 10 days).

After 10 days, when the DI-ESI-MS displayed in Figure SI19 with the mass at 670 m/z assigned to the target complex **2** as dominant signal was recorded, the reaction was allowed to cool down. All attempts to isolate the pure imine interlinked complex failed. However, the crude reaction mixture enabled to record a high resolution mass spectrum of the complex **2**.

The analysis of the crude reaction mixture by ^{1}H-NMR spectroscopy in acetone-d_6 is displayed in Figure SI20. The signal at 9.87 ppm indicates the presence of benzaldehyde type structures in the reaction mixture, pointing either at partial incomplete closing of the cage or a reopening of the labile imine bonds. Attempts to increase the concentration of the caged target structure **2** failed. The decomposition of the complex during the purification attempts pointed at too labile imine bonds and triggered the here reported interlinking by *Wittig*-chemistry.

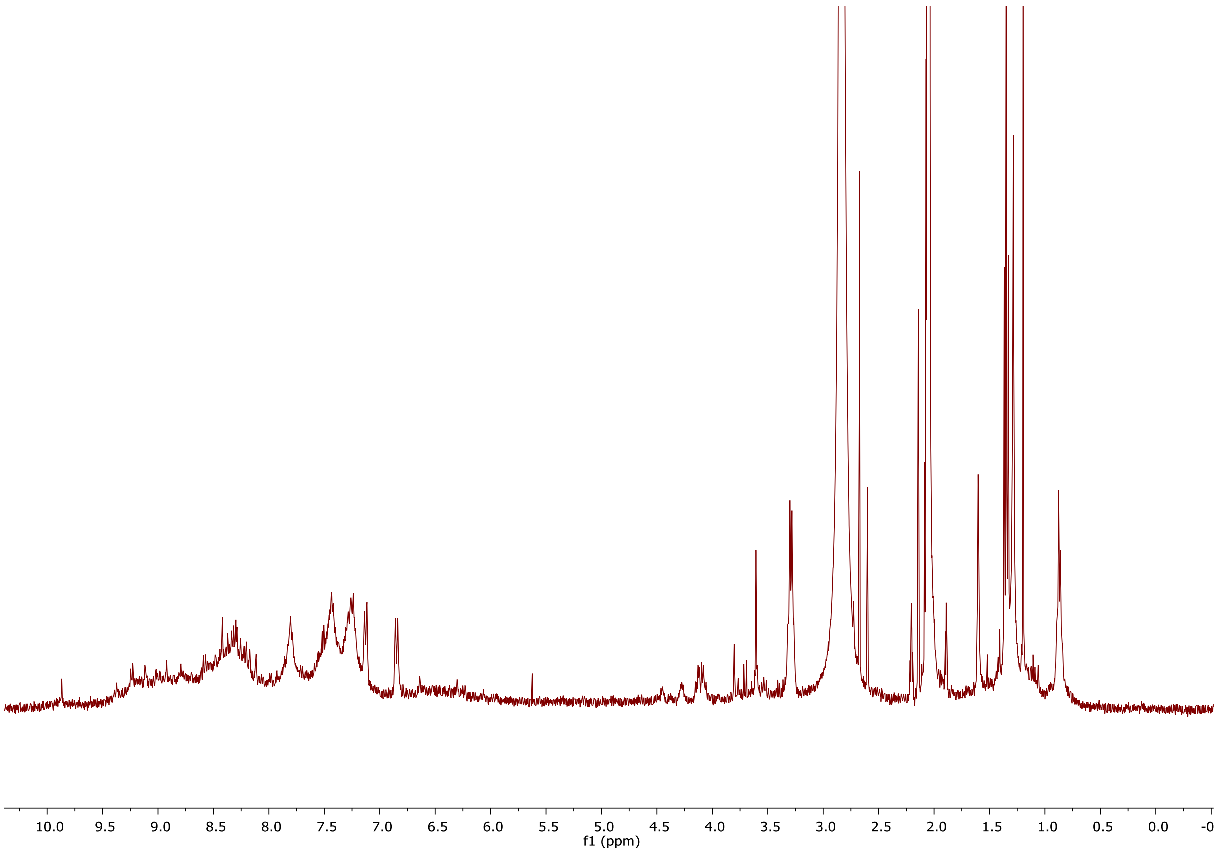

Figure SI20: ^{1}H NMR spectrum of the crude reaction mixture of the imine condensation recorded in acetone-d_6.

Scanning Tunneling Microscope (STM) Experiments of Complex 1

Clean and flat Au(111) surfaces were obtained by repeated cycles of Ar-ion sputtering and subsequent annealing to 500 °C.

The deposition of **1** was performed with a home-built electrospray setup[6] using a solution consisting of **1** dissolved in acetonitrile. The mass spectrum of the solution, acquired prior to deposition, exhibits a single peak with a mass to charge ratio at approximately 660 u/e, which is compatible with the expected mass to charge ratio of 665.7087 u/e within the accuracy of the mass spectrometer used.

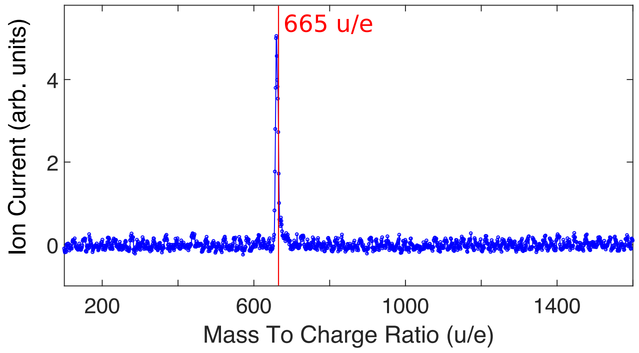

Figure SI21: Mass spectrum of **1** dissolved in acetonitrile prior to deposition.

The measurements were performed with a scanning tunneling microscope operated at low temperature (4.4 K) in ultra-high vacuum. STM tips were electrochemically etched from tungsten wire and further prepared in situ by indentation in the Au(111) substrate.

Fe(II)tpy$_2$ Model Complexes of Varying Rigidity in STM Experiments

The interdependence of the coordination sphere geometry and the spin-state of the central Fe(II) ion makes Fe(II)tpy$_2$ complexes interesting model compounds for manipulating the molecular spin-state in STM experiments. To illustrate the unique stability of the coordination sphere of **1** on Au(111) in low-temperature STM experiments, its behavior is compared to less rigid Fe(II)tpy$_2$ model complexes that were previously analyzed in similar experiments.

Figure SI22 shows the structures together with STM measurements of three Fe(II)tpy$_2$ model complexes with increasing numbers of linkers between the terpyridine ligands. As previously discussed in Ref. [7], the compound without any linkers between both tpy-subunits (Figure SI22 a) was found fragmented on Au(111). A majority of fragments assembles into irregular clusters with varying numbers of ligands and Fe atoms. Occasionally, flat dimers are observed (Figure SI22 d). The apparent height of the ligands is approximately 200 pm. We

[6]Chr. Hamann, R. Woltmann, I.-Po Hong, N. Hauptmann, S. Karan, R. Berndt, *Rev. Sci. Instrum*. **2011**, *82*, 033903.
[7]T. Knaak, C. González, Y. J. Dappe, G. D. Harzmann, T. Brandl, M. Mayor, R. Berndt, M. Gruber, *J. Phys. Chem. C* **2019**, *123*, 4178–4185.

note that apparent height is related to the overlap of wave functions and may therefore differ from the geometric height.

Fragmentation on the surface is prevented by incorporating two linkers between the two tpy-ligands (Figure SI22 b). However, as displayed in Figure SI22 e, the complex is planarized on the surface because of the strong dispersion forces exerted by the metal substrate. The planarization is evident from the unchanged apparent height of the compound on Au(111) relative to that of the bare ligand.

A drastically different result is observed for the fourfold interlinked Fe(II)tpy$_2$ complex **1** (Figures SI22 c and f), where the apparent height is approximately 3 times larger than that of bare ligands. The large height indicates that the structure of **1** remains three-dimensional with limited surface-induced planarization.

In summary, the comparison of the STM results on the three Fe(II)tpy$_2$ model complexes of increasing rigidity suggest that **1** (4 linkers) is least sensitive to dispersion force induced distortion. This corroborates the molecular design hypothesis of **1** being the most rigid compound.

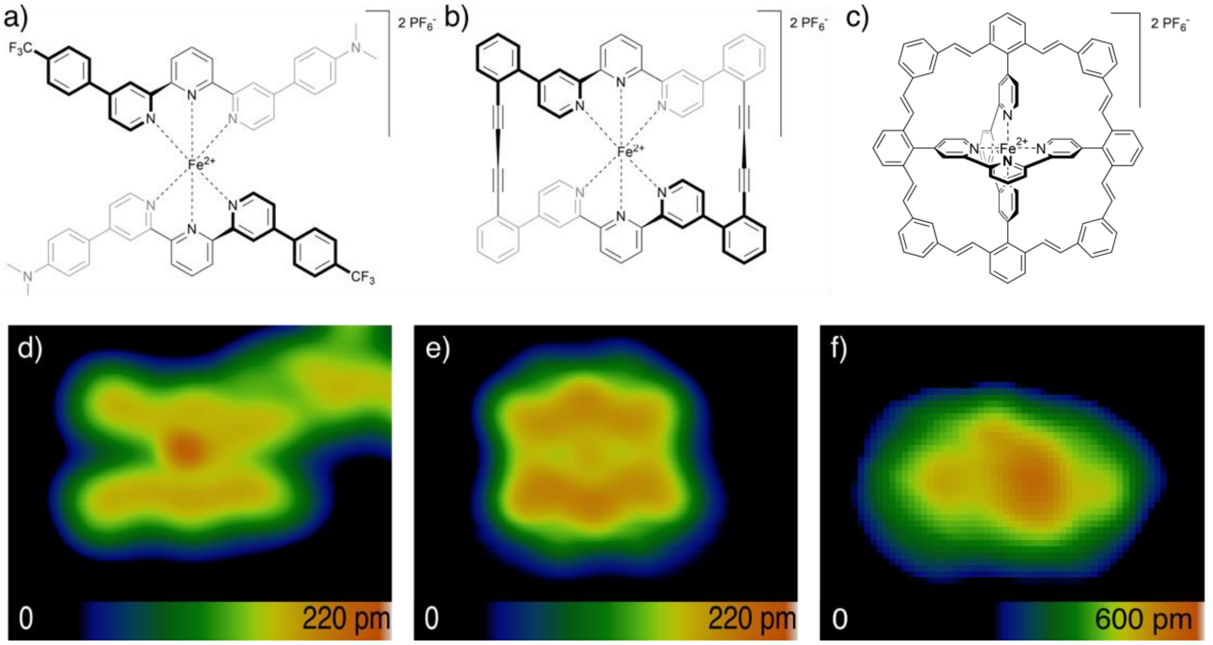

Figure SI22: a−c) Model of complexes with 0, 2 and 4 linkers. d−f) STM topographs (4 nm wide) of the complexes a−c) adsorbed on Au(111). Note the different height scale in (f). Details of the sample preparation and measurements of d) and e) are given in Ref. [7]. Tunneling conditions: d) I = 30 pA, V = 50 mV, e) I = 200 pA, V = 100 mV and f) I = 5 pA, V = -500 mV.

HPLC Chromatograms of Complex 1

In a first HPLC run (for further details see experimental procedures) the threefold interlinked intermediate and the condenstation product with more than four diamines per complex were separated from the product. The all-*trans* isomer was isolated from other isomers by two different applied reversed-phase (C18) HPLC conditions (see figure SI22). Both times a 7:3 mixture of MeCN:H_2O, but once with 10 mM NH_4PF_6 in both solvents and once with 10 mM NH_4PF_6 in water and 0.1% TFA in MeCN. The chromatograms are given at 360 nm and 371 nm, respectively. In both cases the almost perfectly baseline separation of the all-*trans* isomer could be achieved. In the chromatogram a shoulder for both marked peaks can be seen on the right side. Therefore, only the bluely marked part was isolated to prevent the impure isolation of the isomer. However, this and the fact that several different isomers are formed (theoretically 64 different isomer) led to the rather low yield of 4%.

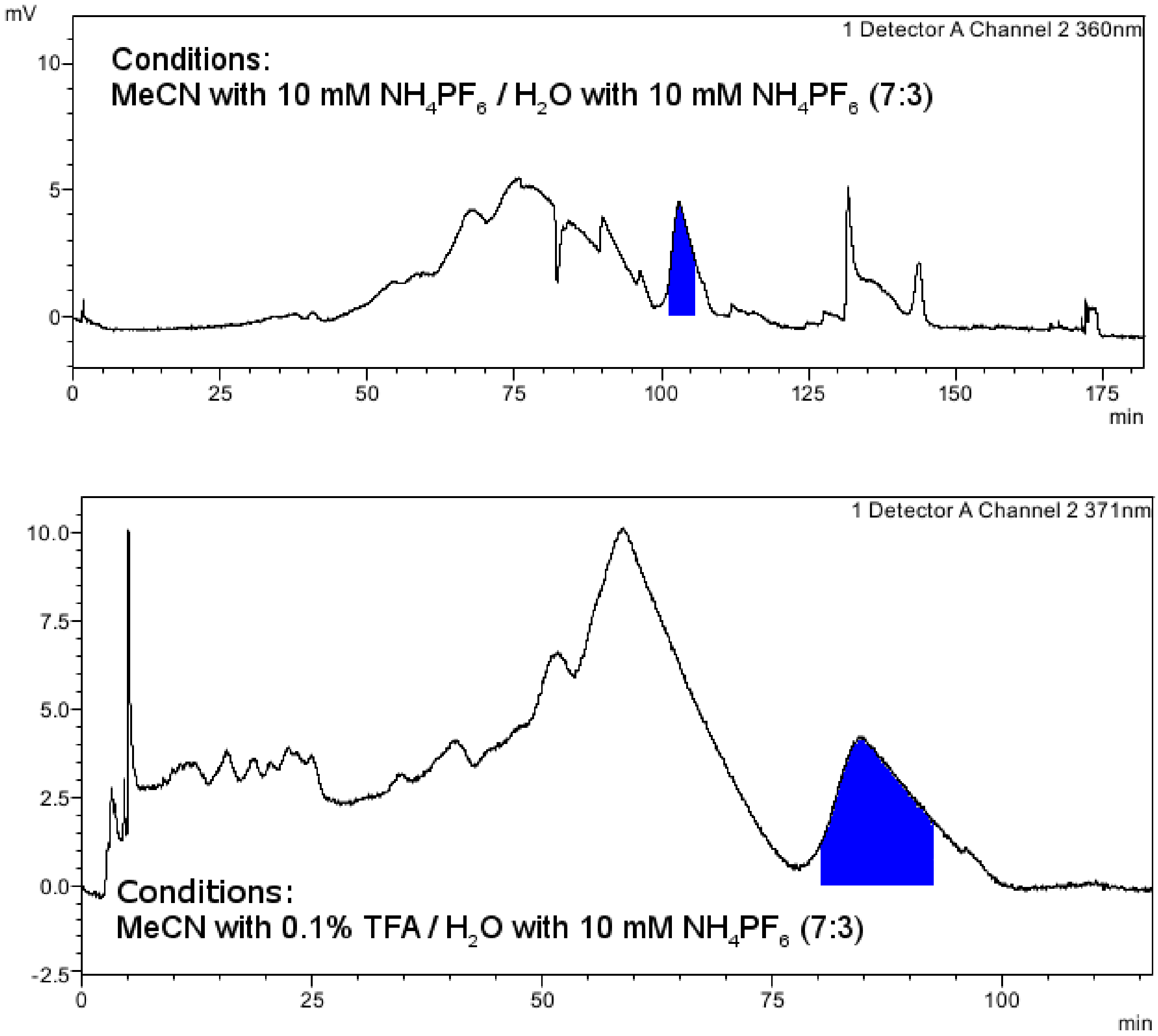

Figure SI23: HPLC Traces of complex **1** with different applied conditions. Both conditions yielded the isolation of the all-*trans* isomer corresponding to the peak marked in blue.

DFT Calculations

An isodesmic reaction, where the type of chemical bonds broken in the reactant are the same as the type of bonds formed in the reaction product, can be used as a theoretical tool to evaluate the strain in a molecule. In the case of **1** it is important to partitioning the molecule carefully to satisfy this requirement. It was achieved by introducing 4 4-phenylpyridine units that then combine with **1** to create a ring system without any possible crowding alongside a bis-[Fe(diphenyl-2,2':6',2"-terpyridine)]$^{2+}$ complex. DFT calculated *Heat of Formation* values were used to estimate the energy increase caused by steric crowding in **1**. It was found that the ring strain energy compares to that of a cyclononane or cyclodecane, which, considering the large ring system of **1**, can be considered as negligible (Figure S124).

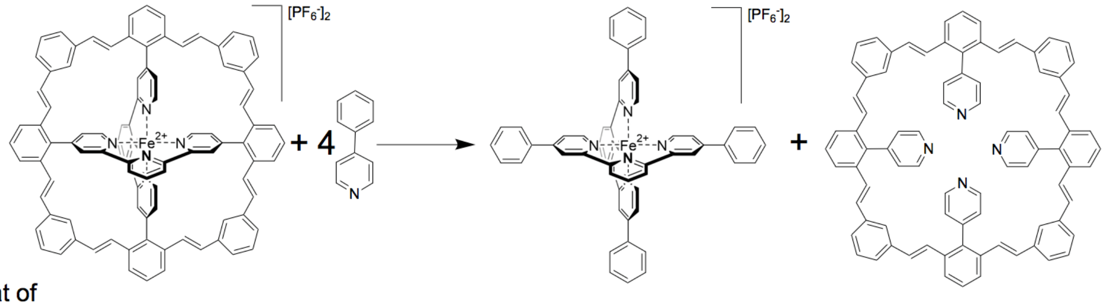

Heat of Formation / Hartrees -5211.2774056 4x -479.3570531 -3672.6006644 -3456.12997797

Total Calculated Ring Strain Energy: 15.7 kcal/mol

Figure SI24: The strain of complex **1** was estimated by investigating the depicted isodesmic reaction. DFT calculations (B3LYP/6-31G (d,p)) were used to access the heat of formation of the 4 species involved in this reaction.
